# Prokaryotic expression, purification and evaluation of anti-cardiac fibrosis activity of recombinant TGF-β latency associated peptide

**DOI:** 10.7717/peerj.12797

**Published:** 2022-01-19

**Authors:** Xudong Song, Yufei Qiu, Jiayi Shi, Luxin Li, Xiaohuan Yuan, Dan Wu, Yanhui Chu

**Affiliations:** 1 Heilongjiang Key Laboratory of Anti-fibrosis Biotherapy, Mudanjiang Medical University, Mudanjiang, Heilongjiang, China; 2 Medical Research Center, Mudanjiang Medical University, Mudanjiang, Heilongjiang, China

**Keywords:** TGF-β1, Latency associated peptide, Cardiac fibrosis, Prokaryotic expression, Protein purification

## Abstract

**Background:**

Cardiac fibrosis refers to the abnormal accumulation of extracellular matrix in the heart, which leads to the formation of cardiac scars. It causes systolic and diastolic dysfunction, and ultimately leads to cardiac dysfunction and arrhythmia. TGF-β1 is an important regulatory factor involved in cardiac fibrosis. Studies have shown that the N-terminal latency associated peptide (LAP) must be removed before TGF-β1 is activated. We hypothesize that recombinant LAP may inhibit cardiac fibrosis induced by TGF-β1. To evaluate anti-cardiac fibrosis activity of recombinant LAP, an experimental study was carried out and is reported here.

**Methods:**

The pET28a-LAP plasmid was constructed and transformed into *E. coli* C43 (DE3) competent cells. The recombinant LAP protein was purified by Ni affinity chromatography. The cells were treated with TGF-β1 at different concentrations for 24 h. The expression of α-SMA was detected by Western blot. RTCA was used to detect the effect of recombinant LAP on the proliferation of H9C2 cells induced by 10 ng/mL TGF-β1. To detect the effect of LAP on the expression of fibrosis-related proteins, H9C2 cells were treated with 10 ng/mL TGF-β1 for 24 h, then added 60 μg/mL recombinant LAP for 48 h. The LAP group was treated with 60 μg/mL recombinant LAP alone. The LAP pre-protection group was treated with 10 ng/mL TGF-β1 and 60 μg/mL recombinant LAP at the same time. Western blot and immunofluorescence were used to detect the expression of α-SMA, collagen I and fibronectin and p-Smad2.

**Results:**

The recombinant LAP was prokaryotic expressed and purified. 10 ng/mL was determined as the optimal working concentration of TGF-β1 to induce H9C2 cells fibrosis. RTCA results showed that 60 μg/mL LAP could effectively inhibit the proliferation of H9C2 cells induced by TGF-β1. Immunofluorescence results showed that compared with the control group, the fluorescence intensities of α-SMA, collagen I and FN increased significantly after TGF-β1 treatment. The fluorescence intensities in the TGF-β1+LAP group decreased significantly. Western blot results showed that 60 μg/mL LAP could inhibit the increase of α-SMA, collagen I and FN expression in H9C2 cells induced by TGF-β1. Compared with the control, the LAP alone group has no significant difference in α-SMA and p-Smad2 expression level. The expression of α-SMA and p-Smad2 in the TGF-β1 model group was significantly increased compared with the control group. Compared with the TGF-β1 group, both TGF-β1+LAP group and LAP pre-protection group significantly reduced the increase in α-SMA and p-Smad2 levels.

**Conclusions:**

Recombinant LAP was prokaryotic expressed and purified. The results showed that recombinant LAP can inhibit the cell proliferation and expression increase of α-SMA, collagen I, fibronectin and p-Smad2 in H9C2 cells induced by TGF-β1. These results suggested that recombinant LAP might inhibit TGF-β1-induced fibrosis of H9C2 cells through the TGF-β/Smad pathway.

## Introduction

Cardiac fibrosis refers to the abnormal deposition of extracellular matrix in the heart caused by myocardial injury, circulatory system disorders, drugs and other factors, which ultimately leads to the formation of cardiac scars (Hinderer & Schenke-Layland, 2019). A lot of evidence shows that cardiac fibrosis increases the risk of arrhythmia and cardiac dysfunction (Nguyen et al., 2017). Under normal circumstances, when the heart is injured or ischemic, it will be reconstructed, at the beginning of injury, the immune cells in the heart proliferate rapidly and begin to clear away the apoptotic cardiomyocytes and necrotic tissues. At the same time, a large number of pro-fibrosis factors are released (Manabe, Shindo & Nagai, 2002). Under the action of these pro-fibrosis factors, fibroblasts in the heart are activated and secrete extracellular matrix including collagen and fibronectin, forming scar tissue to promote heart repair. When the heart suffers from continuous injury, it will lead to the continuous production of extracellular matrix, which will lead to the deposition and bad remodeling of extracellular matrix in the heart. Non-contractile extracellular matrix will also lead to the dysfunction of cardiac contractility and cause arrhythmia (Li, Zhao & Kong, 2018).

TGF-β is an important regulatory factor in cardiac fibrosis. Previous experimental studies have shown that the expression of TGF-β increases during the process of cardiac fibrosis, which promotes the occurrence and development of cardiac fibrosis by binding to the TGF-β receptor to activate the TGF-β/Smad pathway (Kuwahara et al., 2002). In addition, TGF-β can also aggravate the development of fibrosis by activating fibroblasts and promoting the production of extracellular matrix. At present, three subtypes of TGF-β (TGF-β1, TGF-β2, TGF-β3) have been found in mammals, which are encoded by three different genes (Morikawa, Derynck & Miyazono, 2016). TGF-β needs to undergo intracellular modification before secretion. The most important modification process is to remove the LAP at its N-terminus. LAP and TGF-β are often connected by non-covalent bonds to form latent TGF-β (L-TGF-β). At this time, TGF-β has no activity and cannot bind to the receptor to play its role (Robertson et al., 2015). Therefore, removal of LAP is essential for the activation of TGF-β. Inhibiting TGF-β through LAP may be an effective approach to treat fibrotic diseases.

To investigate the effect of LAP on cardiac fibrosis induced by TGF-β1, a prokaryotic expression vector of pET28a-LAP was constructed based on the human full-length LAP sequence and transformed into *E. coli* C43 (DE3) cells. The recombinant LAP protein was purified by Ni-affinity chromatography. Then, the effects of recombinant LAP protein on the proliferation and the expression of fibrosis-related proteins of H9C2 cells induced by TGF-β1 were detected by RTCA, Western blot and Immunofluorescence. We hope to provide new ideas for the treatment of cardiac fibrosis and provide experimental basis for the development of anti-fibrosis drugs through this study.

## Materials and Methods

### pET28a-LAP expression plasmid construction

The sequence of human full-length LAP (NM_000660.7, 966-1712) was used to design primers. The primers were synthesized by Jin Weizhi Biotechnology Co., Ltd. The target fragment was obtained by PCR and digested by EcoRI and XhoI restriction endonucleases (Thermo Scientific, Waltham, MA, USA). The digested product was ligated with the pET-28a vector at 4 °C for 12 h. The ligation products were transformed into *E. coli* C43 (DE3) competent cells (Novagen, Vadodara, Gujarat). Positive clones were screened by PCR and cultured to extract plasmids. The plasmids were sequenced by Jin Weizhi Biotechnology Co., Ltd. The sequencing results were aligned by MegAlign.

### Prokaryotic expression and purification of recombinant LAP

Positive colonies were cultured and 0.2 mM isopropyl-β-D-1-thiogalactopyranoside (IPTG, BioFroxx, Germany) was added to induce protein expression at 16 °C. As it carries the His tag, the LAP protein was purified by Nickel affinity chromatography. The eluted protein was detected by SDS-PAGE and western blot. The elute protein was purified by Superdex 200 10/300 filter chromatography column (GE Healthcare, Chicago, IL, USA). The protein purification and molecular weight determination methods were as previously reported (Wu et al., 2021a). The calculation formula of the partition coefficient (Kav) was: Kav = (VE−V0)/(VT−V0). The standard curve was established by Kav and the logarithm of molecular weight.

### Pull down assay

A total of 20 μg recombinant LAP protein and 50 μL Ni-NTA agarose were incubated at 4 °C for 1 h. The Ni-NTA agarose was washed with 500 μL Buffer B for 3 times. Then 50μL TGF-β1 (10 μg/mL) was added into the LAP-Ni-NTA agarose and incubated at 4 °C for 1 h. The protein-free Ni-NTA agarose and the same amount of TGF-β1 were incubated at 4 °C for 1 h as control. After washing with 500 μL Buffer B for 3 times, Ni-NTA agarose was collected and identified by SDS-PAGE and Western blot by using anti-His Tag antibody (ABclonal, Woburn, MA, USA, 1:5,000 dilution), anti-TGF-β1 antibody (Abcam, Waltham, MA, USA, 1:1,000 dilution) and goat anti-mouse second antibodies labeled with biotin (Zhongshan-golden bridge, China, 1:10,000 dilution).

### Cells and culture conditions

The rat H9C2 cells (iCell Bioscience Inc, Shanghai, China) were cultured in DMEM/F-12 medium (Logan, Utah, MA, USA), which was supplemented with 1% penicillin/streptomycin (Gibco, BRL, Waltham, MA, USA) and 10% fetal bovine serum (FBS, Gibco, Waltham, MA, USA). The cells were cultured in an incubator containing 5% CO_2_, 37 °C and saturated humidity.

### Working concentration screening of TGF-β1 induced H9C2 cell fibrosis

H9C2 cells in the logarithmic growth phase were selected, and the cell density was adjusted to 1 × 10^5^/mL. The cells were seeded in a six-well plate with 2 mL medium per well. After the cells completely adhered to the wall, the cells were treated with TGF-β1 (PeproTech, Cranbury, NJ, USA) at different concentrations (5, 10, 20, and 30 ng/mL) for 24 h. Then, the expression level of α-SMA in H9C2 cells was detected by Western blot.

### Detection of cell index in H9C2 cells by RTCA

To detect the effect of recombinant LAP protein on the proliferation of H9C2 cells induced by TGF-β1, 100 μL H9C2 cell suspension grown in the logarithmic phase was inoculated into an E-plate16 (5,000 cells/well). After the cells were completely attached to the wall, 10 ng/mL of TGF-β1 and different concentrations (30 and 60 μg/mL) of the recombinant LAP protein were added. The cell index (CI) was detected by a real-time cell analysis (RTCA) system, and there was a positive correlation between the cell index and the cell survival rate.

### Detection of fibrosis-related proteins expression in H9C2 cells by Western blot

H9C2 cells in the logarithmic growth phase were selected, and the cell density was adjusted to 1 × 10^5^/mL. The cells were seeded in a six-well plate. After the cells completely adhered to the wall, the model group and the treatment group were treated with TGF-β1 (PeproTech, Cranbury, NJ, USA) at 10 ng/mL for 24 h. After changing the culture medium, 60 μg/mL of the recombinant LAP protein was added into the treatment group, and the cells were collected after 48 h. The LAP group was treated with 60 μg/mL recombinant LAP protein. The pre-protection group was treated with 10 ng/mL TGF-β1 and 60 μg/mL recombinant LAP protein at the same time.

The H9C2 cells were lysed with RIPA buffer (Solarbio, Beijing, China) at 4 °C for 30 min. Then, the supernatant was collected by centrifugation at 12,000 g for 10 min. The protein concentration was determined by Nanodrop 2,000 (Thermo Scientific, Waltham, MA, USA). SDS-PAGE electrophoresis was carried out using 50 μg of protein per well. Then, the proteins were transferred to a PVDF membrane and were blocked with 5% skimmed milk powder for 1 h at room temperature. Then, a rabbit anti-mouse collagen I monoclonal antibody (Affinity Biosciences, Cincinnati, OH, USA, 1:1,000 dilution), a rabbit anti-mouse α-SMA monoclonal antibody (Affinity Biosciences, Cincinnati, OH, USA, 1:1,000 dilution), a rabbit anti-mouse FN monoclonal antibody (Affinity Biosciences, Cincinnati, OH, USA, 1:1,000 dilution), a rabbit anti-mouse Smad2 monoclonal antibody (CST, USA, 1:1,000 dilution) and a rabbit anti-mouse p-Smad2 monoclonal antibody (CST, USA, 1:1,000 dilution) were added separately overnight at 4 °C. After TBST washing, the membranes were incubated with goat anti-rabbit second antibodies labeled with biotin (Zhongshan-golden bridge, China, 1:10,000 dilution) at room temperature for 1 h. Then, the membranes were washed in TBST 3 times, each time for 10 min. ECL chemiluminescence reagent (Solarbio, Beijing, China) was added, and an Amersham Imager 600 ultra-sensitive multi-function imager was used to calculate and analyze each band.

### Detection of fibrosis-related proteins expression in H9C2 cells by Immunofluorescence

Approximately 30,000 cells were added to each well of the six-well plate with cover slides. After the cells attached, TGF-β1 (10 ng/mL) was added into each well. After 24 h, the recombinant LAP protein (60 μg/mL) was added into the cells for 48 h. The cells were fixed with 4% paraformaldehyde, permeabilized with 0.1% TritionX-100 for 15 min, and blocked with 5% BSA at room temperature for 1 h. Then, a rabbit anti-mouse collagen I monoclonal antibody (Affinity Biosciences, Cincinnati, OH, USA, 1:200 dilution), a rabbit anti-mouse α-SMA monoclonal antibody (1:200 dilution) and a rabbit anti-mouse FN monoclonal antibody (1:200 dilution) was added overnight at 4 °C. The cells were washed with PBS 3 times for 10 min each time. Then, a Cy3 labeled donkey anti-goat second antibody (1: 500 dilution) was added at room temperature and in the dark for 1 h. After washing with PBS 3 times, the cells were stained with DAPI for 20 min, fixed with neutral resin, and observed by confocal microscopy.

### Statistical analysis

The experimental data obtained in this study were statistically analyzed by GraphPad Prism8.0 software. The data was expressed as the average and standard deviations. The differences among the groups were analyzed by a single factor analysis of variance (ANOVA). When *P* < 0.05, the data was considered statistically significant.

## Results

### Cloning, expression and purification of the recombinant LAP

**Figure 1:**
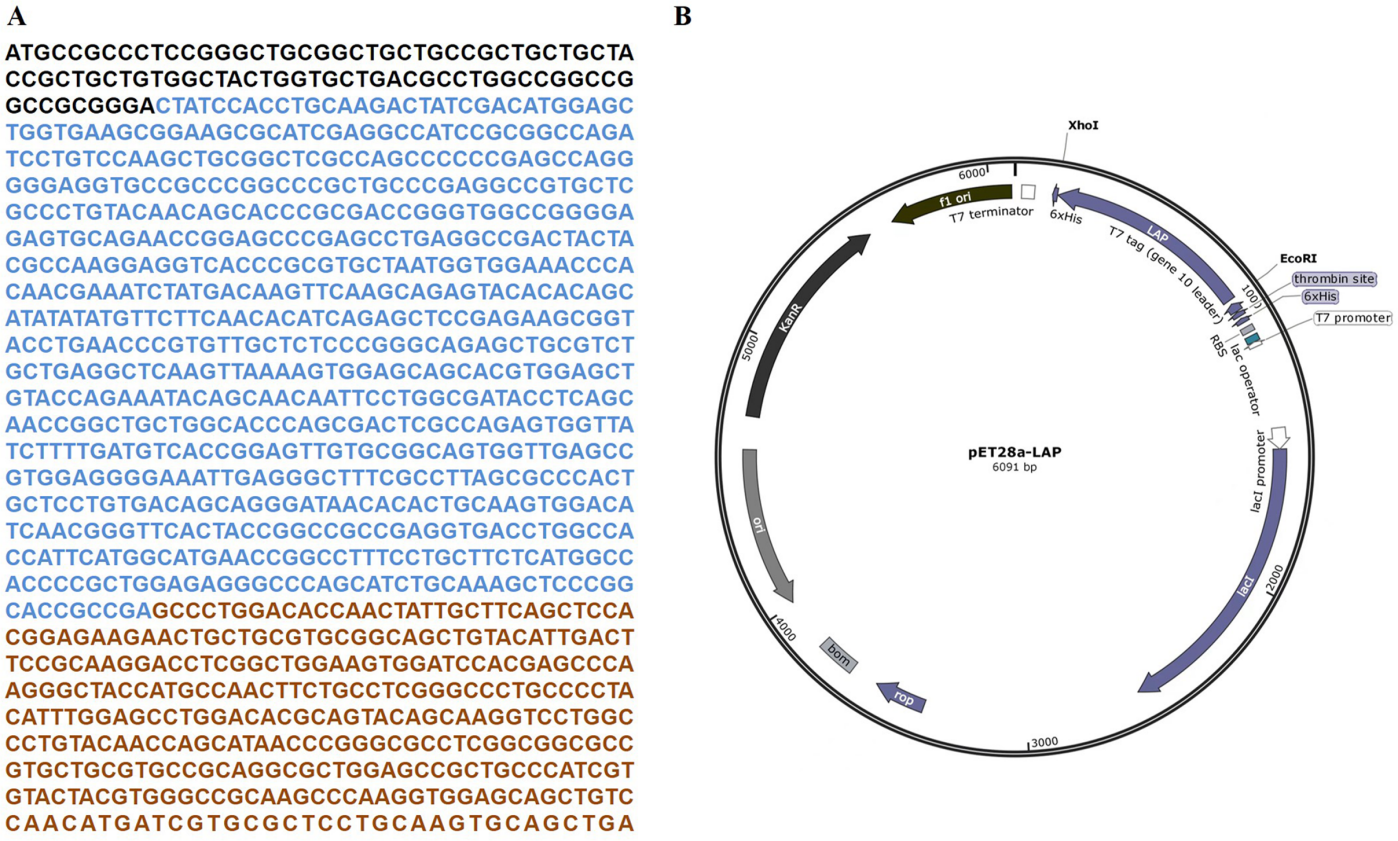
LAP coding sequence and pET28a-LAP plasmid construction. (A) The coding sequence of TGF-β 1 preproprotein. LAP: Blue font; TGF-β: Orange font. (B) Schematic diagram of pET28a-LAP plasmid.

The target LAP fragment of 747bp was designed according to the human LAP gene sequence (Fig. 1A). The LAP fragment was ligated with the vector pET28a by the restriction endonucleases Xhol I and *Eco*R. and successfully constructed the pET28a-LAP plasmid (Fig. 1B).

**Figure 2:**
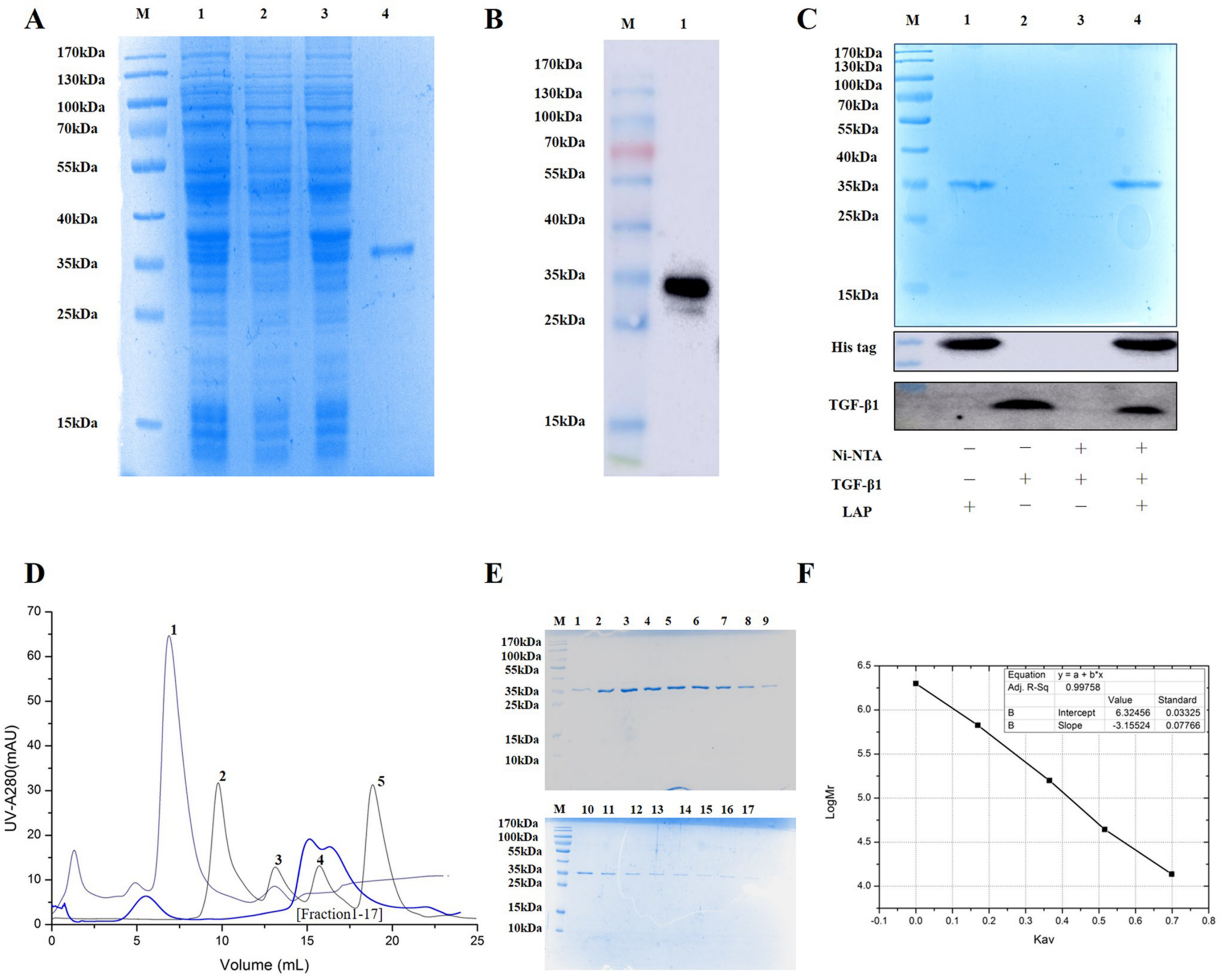
Purification and identification of Recombinant LAP. (A) Purification of recombinant LAP. Lane M, protein marker; lane 1, Crushing bacterium fluid; lane 2, Supernatant; lane 3, Precipitation after crushing; lane 4, elution protein. (B) Identification of recombinant LAP by Western blot. Lane M, protein marker; lane 1, Ni NTA affinity purified protein. (C) Identification of recombinant LAP by pull down assay. M, protein marker; lane 1, LAP protein; lane 2, TGF-β1; lane 3, TGF-β1 + Ni-NTA agarose; lane 4, TGF-β1 +Lap-Ni-NTA agarose. (D) Gel filtration chromatography profile of LAP protein. The thick blue lines are LAP. The thin lines are protein standards, 1. Blue dextran (2000 kDa), 2. Thyroglobulin (669 kDa), 3. Aldolase (158 kDa), 4. Ovalbumin (44 kDa), and 5. Ribose Nuclease A (13.7 kDa). (E) SDS-PAGE (12%) analysis of LAP protein purified by Superdex 200 10/300. M, protein marker; 1–17, fractions1–17 of Peak LAP. (F) The standard curve was established by Kav and the logarithm of protein molecular weight. The analysis result showed that y = −3.15524x + 6.32456, R2 = 0.99758.

The experimental analysis showed that the target protein had a good expression level with 0.2 mM IPTG at 16 °C for 14 h. Since the recombinant LAP carries histidine (His) tag, the Recombinant LAP protein was purified by Ni-NTA agarose (Spriestersbach et al., 2015). The SDS-PAGE analysis showed that the recombinant protein was mainly expressed at 35 kDa, which was consistent with the molecular weight of the target LAP protein (Fig. 2A). The recombinant protein was verified by Western blot. It was identified with mouse anti-His Tag monoclonal antibody, and there was a band at 35kDa, which corresponds to the target LAP protein (Fig. 2B). These results indicated that the target lap protein was successfully obtained through prokaryotic expression and Ni-affinity chromatography. The recombinant protein was identified by using pull down assay (Fig. 2C). Since TGF-β1 protein does not carry His tag, it cannot bond to Ni-NTA agarose (lane 3). When TGF-β1 was added into LAP-Ni-NTA agarose, western blotting analysis showed the presence of TGF-β1 on Ni-NTA agarose (lane 4), suggesting that TGF-β1 may be loaded onto Ni-NTA agarose by binding to LAP protein.

The eluted LAP protein was purified and measured by using gel filtration chromatography. The result showed that LAP exhibited two peaks (Figs. 2D, 2E). The molecular weight was determined by using gel filtration calibration kits. The standard curve was established by Kav and the logarithm of protein molecular weight. The analysis result showed that y = −3.15524x + 6.32456, R2 = 0.99758 (Fig. 2F). Calculated according to the retention volume of the LAP peaks, the molecular weights are approximate 64 kDa and 32 kDa, respectively. The results suggest that the recombinant LAP may be composed of dimers and a part of monomers.

### Working concentration determination of TGF-β1 induced H9C2 cell fibrosis

**Figure 3:**
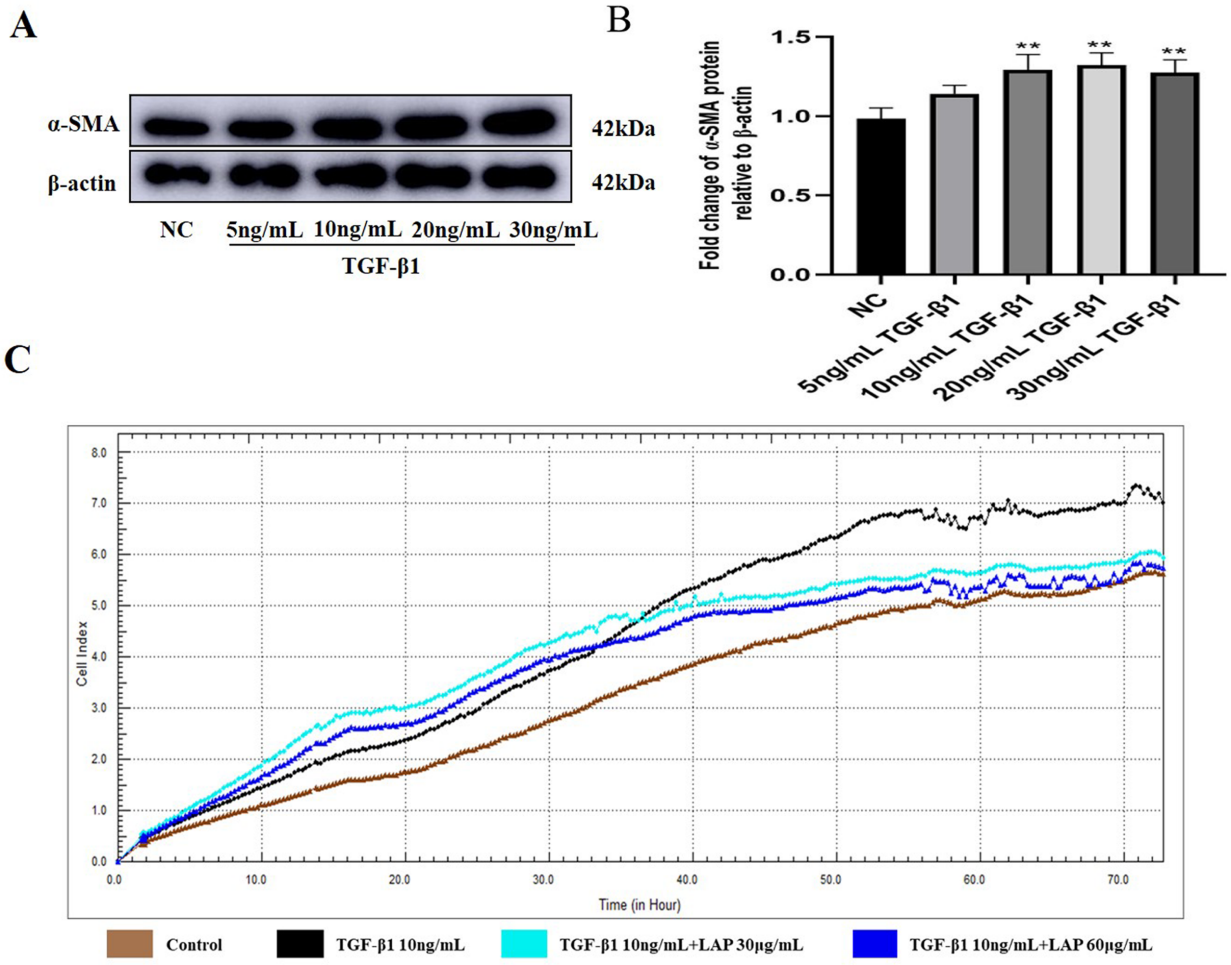
The recombinant LAP inhibits the proliferation of H9C2 cells induced by TGF-β1. (A) Western blot of α-SMA expression in H92C cells treated with different concentrations of TGF-β1. (B) Semi-quantitative analysis of α-SMA expression presented as the relative ratio to β-actin. NC *vs* 10 ng/mL TGF-β1, *P* = 0.0083; NC *vs* 20 ng/mL TGF-β1, *P* = 0.0038; NC *vs* 30 ng/mL TGF-β1, *P* = 0.006; 10 ng/mL *vs* 20 ng/mL TGF-β1, *P* = 0.9821; 10 ng/mL *vs* 30 ng/mL TGF-β1, *P* = 0.9994, *n* = 3, **P < 0.01 vs NC group. (C) Detection of H9c2 cell proliferation by RTCA system.

To determine the optimal working concentration of TGF-β1 in H9C2 cells, the cells were treated with TGF-β1 at different concentrations (5, 10, 20, and 30 ng/mL) for 24 h. Then, the expression levels of fibrosis marker α-SMA were detected by Western blot. As shown in Figs. 3A, 3B, the expression levels of α-SMA treated with 10, 20 and 30 ng/mL of TGF-β1 were significantly increased compared with the control group (NC *vs* 10ng/mL TGF-β1, *P* = 0.0083; NC *vs* 20 ng/mL TGF-β1, *P* = 0.0038; NC *vs* 30 ng/mL TGF-β1, *P* = 0.006, *n* = 3). Compared with the 10 ng/mL TGF-β1treatment group, there was no significant difference in the α-SMA expression levels of the 20 ng/mL and 30 ng/mL TGF-β1 treatment groups (10 ng/mL *vs* 20 ng/mL TGF-β1, *P* = 0.9821; 10 ng/mL *vs* 30 ng/mL TGF-β1, *P* = 0.9994, *n* = 3). Thus, 10 ng/mL was determined as the optimal working concentration of TGF-β1 for subsequent experiments.

### The recombinant LAP inhibits the proliferation of H9C2 cells induced by TGF-β1

In order to explore the effect of recombinant LAP on the proliferation of H9C2 cells, the H9C2 cells were treated with 10 ng/mL TGF-β1 and added into different concentrations (30 and 60 μg/mL) of recombinant LAP protein for intervention. The RTCA system was used to detect the proliferation of H9C2 cells. The cell index (CI) was detected every 1 min for a total of 72 h. 24 h after adding TGF-β1, the CI value of the TGF-β1 group began to gradually increase compared with the CI value of the control group. Since the CI value is positively correlated with cell viability, it proved that the proliferation of H9C2 cells was accelerated. Compared with the TGF-β1 group, the CI value of the recombinant LAP treatment groups increased slowly, and the CI value of 60 μg/mL LAP treatment group was close to that of the control group at 72 h, indicating that the proliferation rate of H9C2 cells in the two groups was close (Fig. 3C). These results showed that the recombinant LAP protein could be effectively inhibit the proliferation of H9C2 cells induced by TGF-β1.

### The recombinant LAP inhibits fibrosis-related proteins expression of H9C2 cells induced by TGF-β1

**Figure 4:**
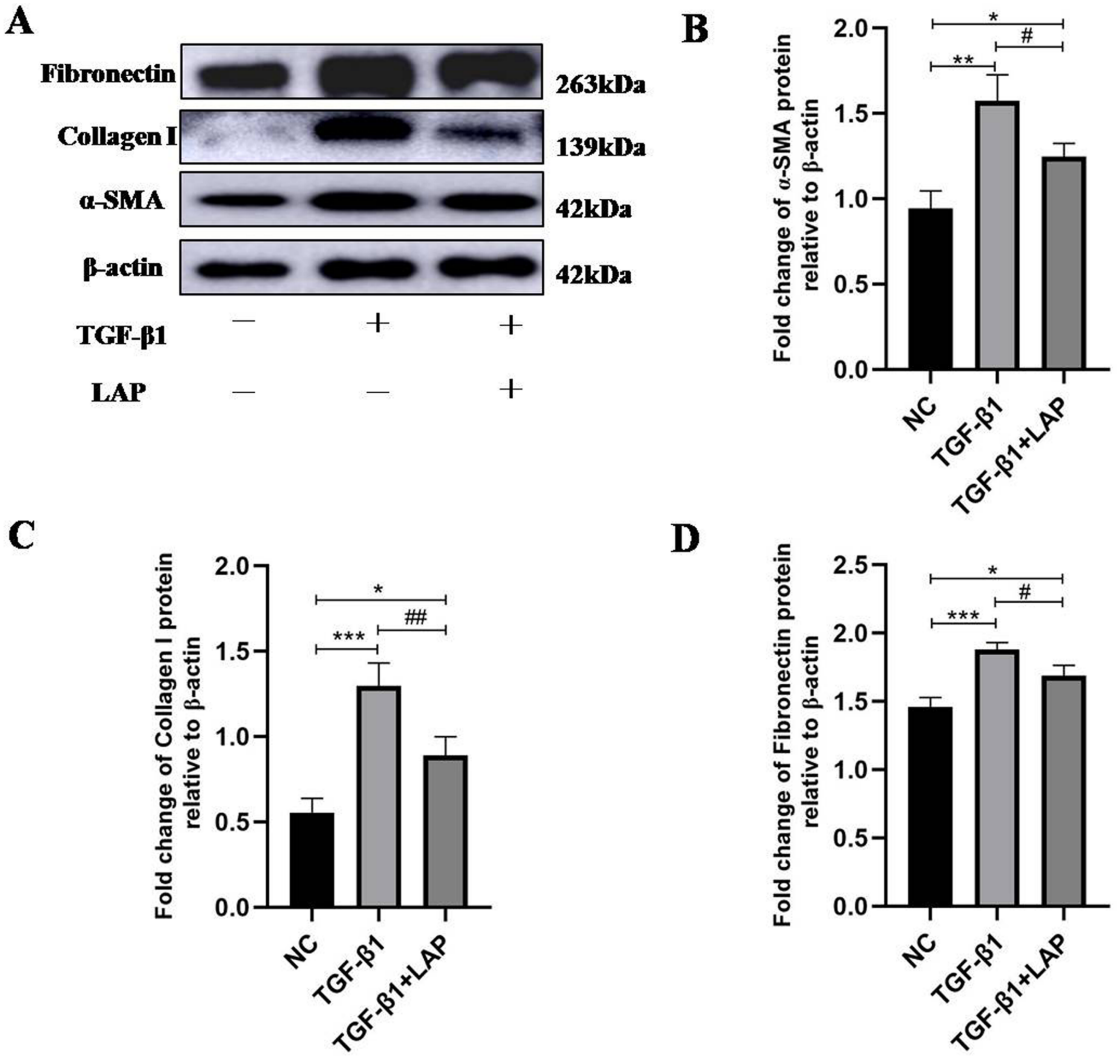
Western blot analysis of the effect of recombinant LAP on fibrosis-related proteins expression in H9C2 cells. (A) Western blot of the effect of recombinant LAP on α-SMA, collagen I and FN expression in H9C2 cells induced by TGF-β1; (B–D) Semi-quantitative analysis of α-SMA, collagen I and FN expression presented as the relative ratio to β-actin. The expression levels of α-SMA, collagen I and FN in H9C2 cells treated with TGF-β1 were significantly higher than the control group (α-SMA, NC *vs* TGF-β1, *P* = 0.0013; collagen I, NC *vs* TGF-β1, *P* = 0.0004; FN, NC *vs* TGF-β1, *P* = 0.0005). Compared with the TGF-β1 group, the expressions of α-SMA, collagen I and FN in the LAP group were significantly reduced (α-SMA, TGF-β1 *vs* TGF-β1+LAP, *P* = 0.0311; collagen I, TGF-β1 *vs* TGF-β1+LAP, *P* = 0.0096; FN, TGF-β1 *vs* TGF-β1+LAP, *P* = 0.0276). NC: control group; TGF-β1: 10 ng/mL TGF-β1 group; TGF-β1+LAP: 10 ng/mL TGF-β1+60 μg/mL LAP group. *n* = 3, *P < 0.05 *vs* NC group; **P < 0.01 *vs* NC group; ***P < 0.001 *vs* NC group; ^#^*P* < 0.05 *vs* TGF-β1 group; ^##^*P* < 0.01 *vs* TGF-β1 group.

To detect the effect of recombinant LAP on the expression of fibrosis-related proteins, H9C2 cells were treated with 10 ng/mL TGF-β1 for 24 h, then changed the culture medium and added 60 μg/mL recombinant LAP for 48 h. As shown in Fig. 4, the expression levels of α-SMA, collagen I and FN in H9C2 cells treated with TGF-β1 were significantly higher than the control group, which confirmed that H92C cells had fibrosis induced by TGF-β1 (α-SMA, NC *vs* TGF-β1, *P* = 0.0013; collagen I, NC *vs* TGF-β1, *P* = 0.0004; FN, NC *vs* TGF-β1, *P* = 0.0005, *n* = 3). Compared with the TGF-β1 group, the expressions of α-SMA, collagen I and FN in the LAP treatment group were significantly reduced (α-SMA, TGF-β1 *vs* TGF-β1+LAP, *P* = 0.0311; collagen I, TGF-β1 *vs* TGF-β1+LAP, *P* = 0.0096; FN, TGF-β1 *vs* TGF-β1+LAP, *P* = 0.0276, *n* = 3).

**Figure 5:**
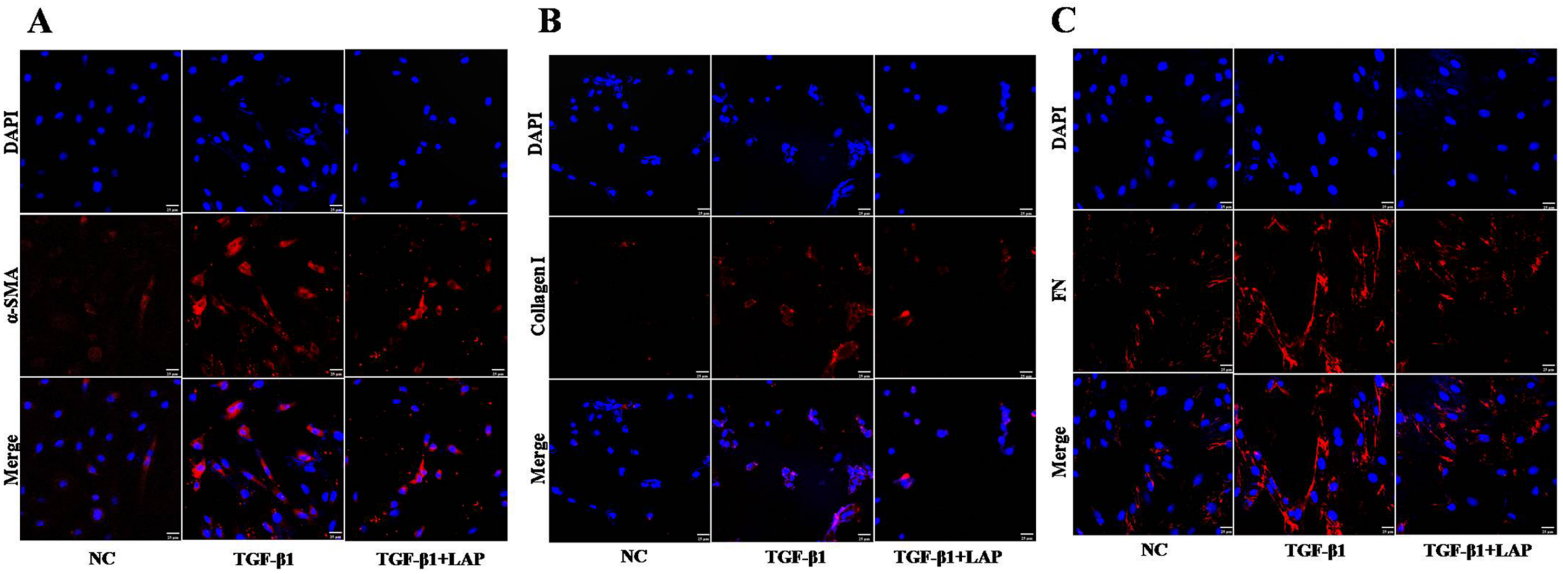
Detection of fibrosis-related proteins expression in H9C2 cells by Immunofluorescence. (A) Immunofluorescence detection of α‑SMA; (B) Immunofluorescence detection of collagen I; (C) Immunofluorescence detection of FN; NC: control group; TGF-β1: 10 ng/mL TGF-β1 group; TGF-β1+LAP: 10 ng/mL TGF-β1+60 μg/mL LAP group. DAPI (blue, nuclear stain) and antibodies to α‑SMA, FN or collagen I (red), immunofluorescence staining (magnification × 400), Scale bars = 25 μm.

To further confirm this result, the fibrosis-related proteins expression in H9C2 cells were detected by immunofluorescence. Compared with the control group, the fluorescence intensity of α-SMA, collagen I and FN increased significantly after the TGF-β1 treatment. In contrast, the fluorescence intensities of these proteins in the LAP treatment group decreased significantly (Fig. 5). The RTCA, Western blot and immunofluorescence results showed that the recombinant LAP protein could be effectively inhibit the proliferation and the expression of fibrosis-related proteins of TGF-β1-induced H9C2 cells. These results indicated that the recombinant LAP had potent anti-cardiac fibrosis activity.

### Recombinant LAP protein inhibited TGF-β1-induced fibrosis of H9C2 cells through TGF-β/Smad pathway

**Figure 6:**
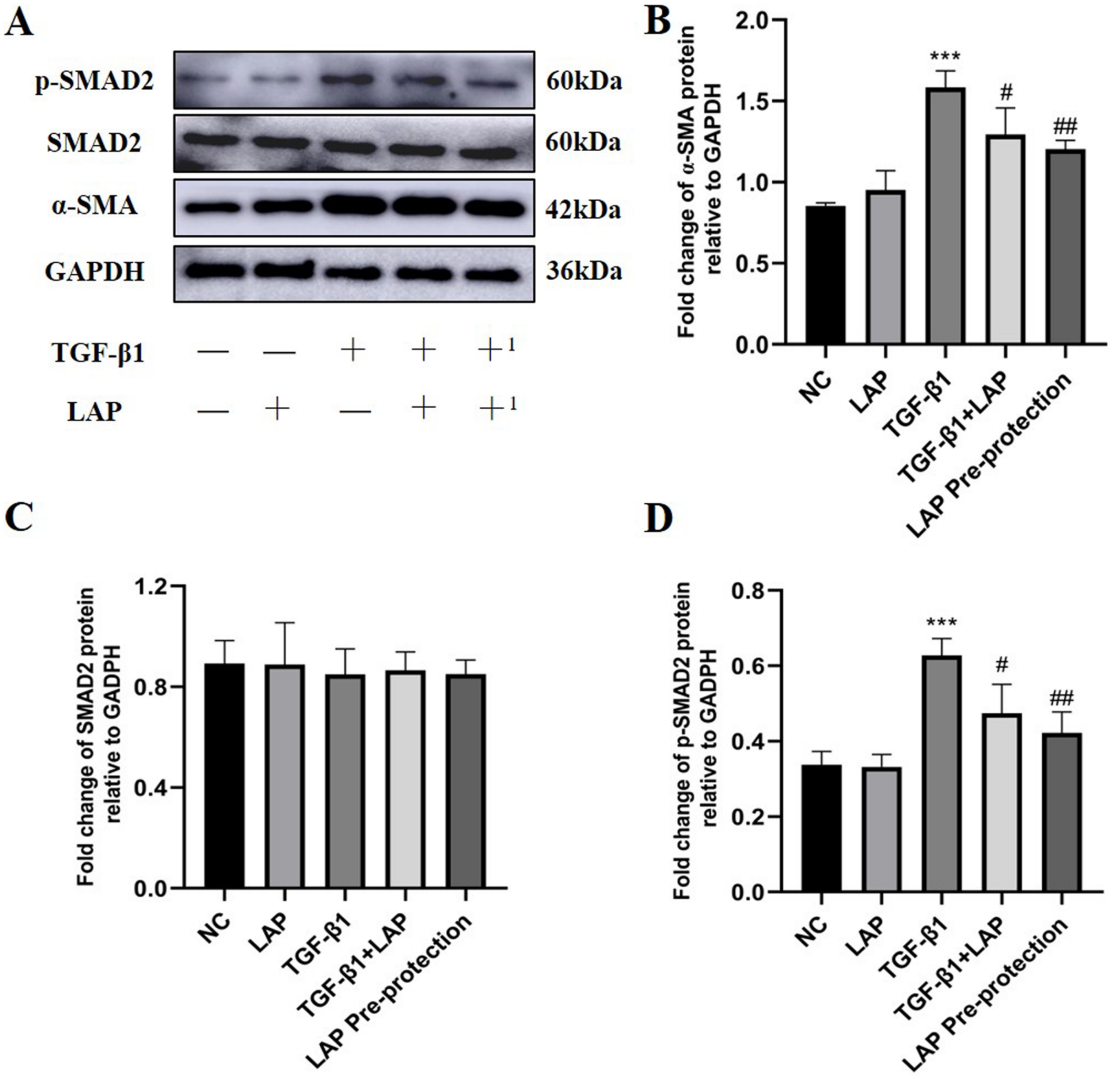
Western blot analysis of the effect of LAP on TGF-β1-induced fibrosis of H9C2 cells through TGF-β/Smad pathway. (A) Western blot of the effect of recombinant LAP on α-SMA, Smad2 and p-Smad2 expression in H9C2 cells induced by TGF-β1; +: TGF-β1+LAP group; +^1^: LAP pre-protection group. (B)–(D) Semi-quantitative analysis of α-SMA, Smad2 and p-Smad2 expression presented as the relative ratio to GAPDH. The expression of α-SMA (*P* < 0.0001) and p-Smad2 (*P* = 0.0003) in the TGF-β1 model group was significantly increased compared with the control group. Compared with the TGF-β1 model group, both TGF-β1+LAP group and LAP pre-protection group significantly reduced the increase of α-SMA (TGF-β1 *vs* TGF-β1+LAP, *P* = 0.0409; TGF-β1 *vs* LAP Pre-protection, *P* = 0.008) and P-Smad2 (TGF-β1 *vs* TGF-β1+LAP, *P* = 0.0285; TGF-β1 *vs* LAP Pre-protection, *P* = 0.0044) levels. NC: control group; LAP: 60 μg/mL LAP group; TGF-β1: 10 ng/mL TGF-β1 group; TGF-β1+LAP: Add 10 ng/mL TGF-β1 first and then 60 μg/mL LAP treatment group; LAP pre-protection group: 10ng/mL TGF-β1 and 60μg/mL LAP at the same time. *n* = 3, ***P < 0.001 *vs* NC group; ^#^*P* < 0.05 *vs* TGF-β1 group; ^##^*P* < 0.01 *vs* TGF-β1 group.

In order to further study the effect of recombinant LAP protein, the LAP group and the LAP pre-protection group were added based on the previous experiment. Then Western Blot was used to detect the expression levels of α-SMA, Smad2 and p-Smad2 in H9C2 cells (Fig. 6A). Compared with the control group, the LAP group with only LAP treatment has no significant difference in the protein expression level. The expression of α-SMA (*P* < 0.0001) and p-Smad2 (*P* = 0.0003) in the TGF-β1 model group was significantly increased compared with the control group. Compared with the TGF-β1 model group, both TGF-β1+LAP group and LAP pre-protection group significantly reduced the increase of α-SMA (TGF-β1 *vs* TGF-β1+LAP, *P* = 0.0409; TGF-β1 *vs* LAP Pre-protection, *P* = 0.008) and P-Smad2 (TGF-β1 *vs* TGF-β1+LAP, *P* = 0.0285; TGF-β1 *vs* LAP Pre-protection, *P* = 0.0044) levels (Figs. 6B–6D). There is no significant difference between TGF-β1+LAP group and LAP pre-protection group. These results suggested that the recombinant LAP protein might inhibit TGF-β1-induced fibrosis of H9C2 cells through the TGF-β/Smad pathway.

## Discussion

Previous experiments have found that regardless of the cause, a continuous fibrotic reaction will occur after heart injury. Abnormal deposition of extracellular matrix is an important cause of cardiac fibrosis, and leads to changes in cardiac structure and cardiac dysfunction (Kong, Christia & Frangogiannis, 2014). Cardiac fibrosis will not only cause the disorder of ventricular diastolic function, but also damage the electromechanical coupling and cause arrhythmia (Chilton, Giles & Smith, 2010). H9C2 is the main effector cell in the process of cardiac remodeling, and the expression level of TGF-β1 in H9C2 cells increases during the process of cardiac fibrosis (Kang et al., 2019). As an essential growth factor for the body, TGF-β1 is one of the most important regulators in the process of cardiac fibrosis. After TGF-β binds to its receptor, it activates the TGF-β/Smad pathway to regulate the production of fibrosis-related proteins (Hu et al., 2018). In addition, TGF-β can also inhibit the activity of matrix metalloproteinases by promoting the production of matrix metalloproteinase inhibitors, leading to reduced ECM degradation and excessive deposition, thereby accelerating the development of fibrosis. Therefore, inhibition of TGF-β and its downstream pathways is an effective approach to treat cardiac fibrosis.

TGF-β1 is widely involved in the pathogenesis of fibrosis. TGF-β1 is often secreted in a latent form and can only be activated by removing its N-terminal LAP protein (Frangogiannis, 2020). At present, LAP protein is considered to be an inhibitor of active TGF-β1(Craven et al., 1997), (Zhang, McCormick & Gilliam, 2003). These results suggested that LAP protein may be a potential therapeutic drug for fibrotic diseases. Therefore, we hypothesized that recombinant LAP protein could inhibit H9C2 cell fibrosis induced by TGF-β1 stimulation. In this study, we constructed pET28a-LAP vector and expressed LAP protein in *E. coli* with fast growth and low cost. A recent experimental study showed that the natural LAP protein was glycosylated, but solution scattering analysis showed that the folding and flexibility of the unbound LAP protein were not affected by glycan modification, so the dimer protein LAP can be effectively expressed in a simple system that does not support post-translational modification (Stachowski, Snell & Snell, 2020). The recombinant LAP protein expressed well at 16 °C with 0.2 mM IPTG for 14 h, and then the recombinant LAP protein was successfully purified by Ni affinity chromatography and gel filtration chromatography. The results suggested that the recombinant Lap may be composed of dimers and a part of monomers. This result was consistent with a previous study about the prokaryotic expression of LAP (Yang, Dignam & Gentry, 1997).

α-SMA is an important marker of fibrosis (Ding et al., 2019). In this study, in order to determine the concentration of TGF-β1, H9C2 cells were treated with different concentrations of TGF-β1 and the level of α-SMA was detected by western blot, The results showed that the optimal working concentration of TGF-β1 to induce fibrosis of H9C2 cells was 10 ng/mL. Then the effect of recombinant LAP protein on the proliferation and fibrosis of H9C2 cells stimulated by TGF-β1 were detected. The results showed that the recombinant LAP protein could effectively inhibit the acceleration of H9C2 cell proliferation and the increase of α-SMA, collagen I and FN levels induced by TGF-β1 stimulation.

Previous studies have shown that αvβ6 has a high affinity for TGF-β1 latency related peptide and is involved in the activation of TGF-β1 latency complex (Annes et al., 2004), soluble LAP protein inhibited the adhesion and migration of oral squamous cell carcinoma mediated by αvβ6 (Thomas et al., 2002). In addition, one study found that TGF-β, erythropoietin, IL-1RA, IL-10, IL-4 and BMP-7 fusion proteins can bind to LAP proteins and become latent, requiring cleavage in their respective bioassages to become active (Mullen et al., 2014). These studies demonstrated the versatility of the LAP protein and demonstrates the feasibility of potential cytokine technologies for disease treatment. In order to further explore how the recombinant LAP protein inhibits fibrosis, we applied LAP protein to H9C2 cells alone and detected fibrosis related markers. It is found that LAP protein alone had no effect on the expression of fibrosis related proteins in H9C2 cells. It is well known that TGF-β1 can activate the TGF-β/Smad pathway by binding to its receptors to promote the occurrence and development of fibrosis. A recent experimental study found that latent TGF-β1 may protect the kidney from TGF-β/smad mediated renal fibrosis by inhibiting arkadia-mediated Smad7 ubiquitin degradation (Wu et al., 2021b). In this study, the expression levels of smad2 and p-Smad2 in H9C2 cells after LAP pre-protection or LAP treatment were detected, and it was found that LAP significantly inhibited the phosphorylation of smad2 induced by TGF-β1. These results suggested that recombinant LAP protein inhibits TGF-β 1-induced fibrosis of H9C2 cells through the TGF-β/Smad pathway.

## Conclusions

In this study, the recombinant LAP was cloned, prokaryotic expressed and purified. The recombinant LAP effectively inhibited TGF-β1-induced proliferation and expression of α-SMA, collagen I, fibronectin and P-Smad2 in H9C2 cells. These results suggested that the recombinant LAP protein might inhibit TGF-β1-induced fibrosis of H9C2 cells through the TGF-β/Smad pathway. This study will provide new ideas for the treatment of cardiac fibrosis and provide experimental basis for the development of anti-fibrosis drugs.

## Supplemental Information

10.7717/peerj.12797/supp-1 Supplemental Information 1 LAP coding sequence information and pET28a-LAP plasmid construction.

10.7717/peerj.12797/supp-2 Supplemental Information 2 Recombinant LAP purification and identification.

10.7717/peerj.12797/supp-3 Supplemental Information 3 TGF-β1 concentration screening by Western blot and RTCA detection of recombinant LAP inhibit the proliferation of H9C2 cells induced by TGF-β1.

10.7717/peerj.12797/supp-4 Supplemental Information 4 Western blot analysis of the effect of recombinant LAP on α-SMA, collagen I and FN expression in H9C2 cells.

10.7717/peerj.12797/supp-5 Supplemental Information 5 Raw data of Immunofluorescence of fibrosis-related protein expression in H9C2 cells.

10.7717/peerj.12797/supp-6 Supplemental Information 6 Raw data of Western blot of the effect of LAP on TGF-β1-induced fibrosis of H9C2 cells through TGF-β/Smad pathway.

## Additional Information and Declarations

### Data Availability

The following information was supplied regarding data availability: The raw data, including SDS-PAGE, RTCA, Western blot and Immunofluorescence results, are available in the Supplemental Files.
