# Supplementary figures and images for: Prokaryotic expression, purification and evaluation of anti-cardiac fibrosis activity of recombinant TGF-β latency associated peptide
Source: PeerJ. 2022 Jan 19;10:e12797. doi: 10.7717/peerj.12797 (PMC8783559; doi:10.7717/peerj.12797)

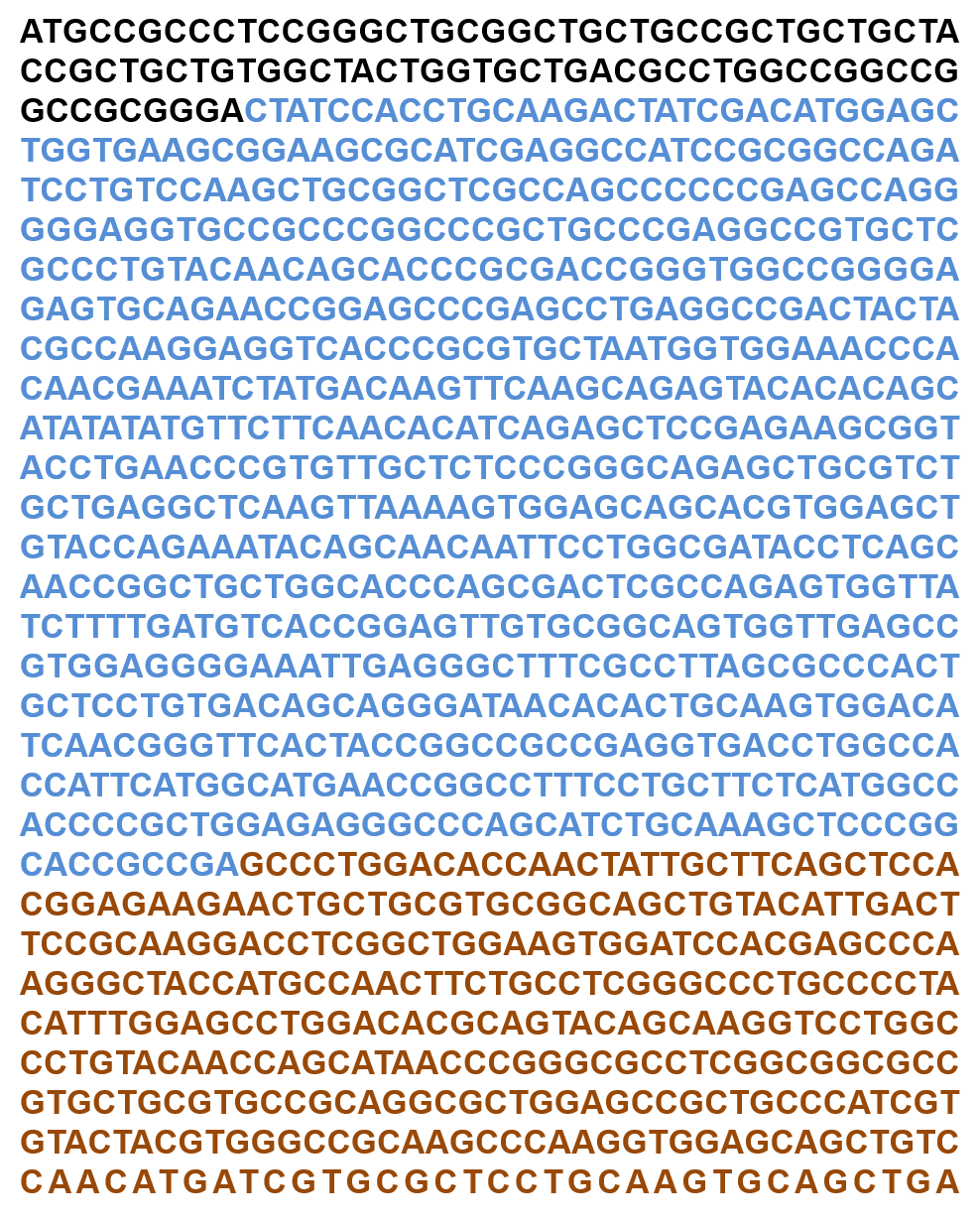

Supplement: Supplemental Information 1 [file peerj-10-12797-s001.zip › Figure-1/1A.png]

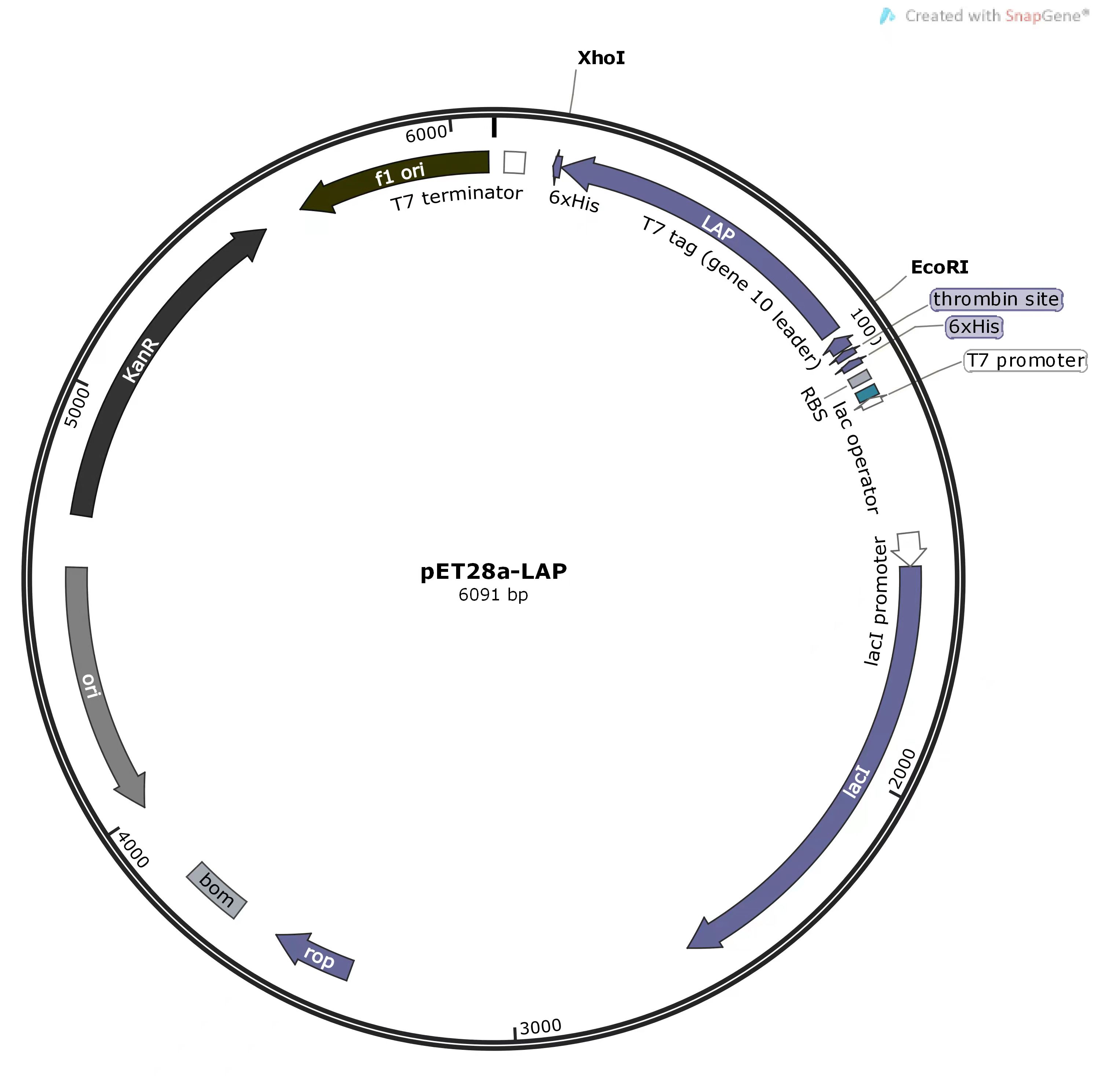

Supplement: Supplemental Information 1 [file peerj-10-12797-s001.zip › Figure-1/1B.jpg]

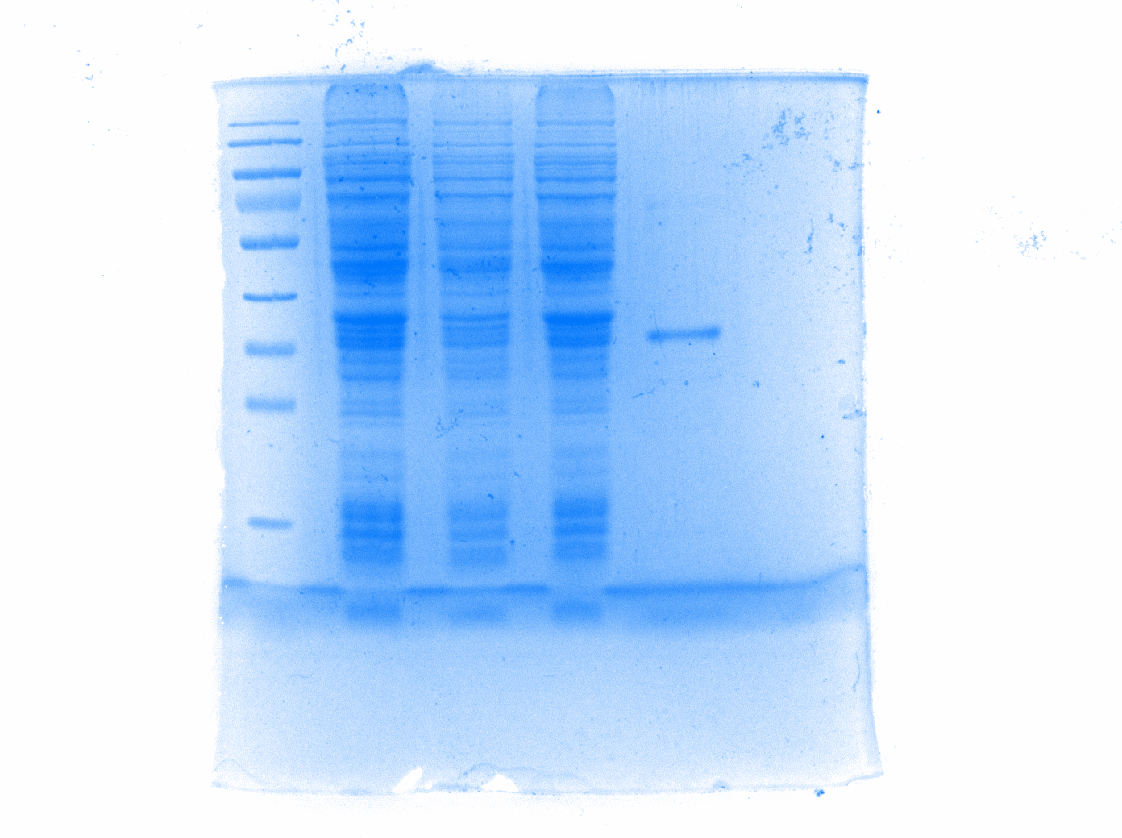

Supplement: Supplemental Information 2 [file peerj-10-12797-s002.zip › Figure-2/A-s1LAP.tif]

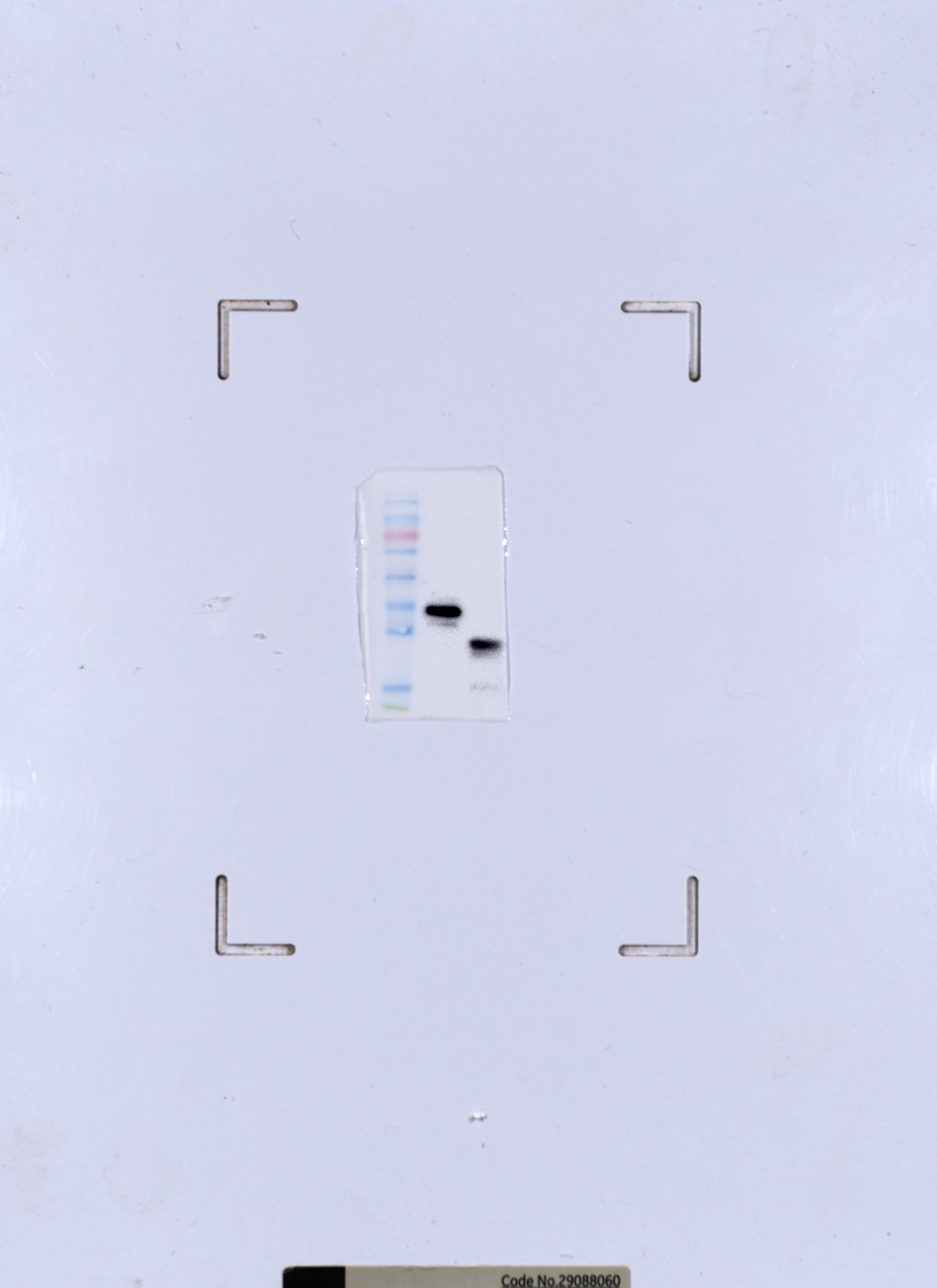

Supplement: Supplemental Information 2 [file peerj-10-12797-s002.zip › Figure-2/B-WBLAP.jpg]

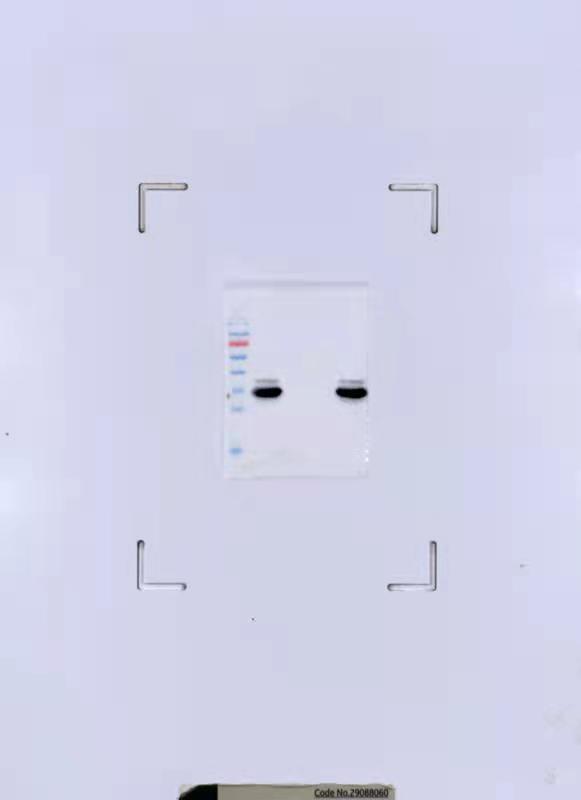

Supplement: Supplemental Information 2 [file peerj-10-12797-s002.zip › Figure-2/C-hiswb.jpg]

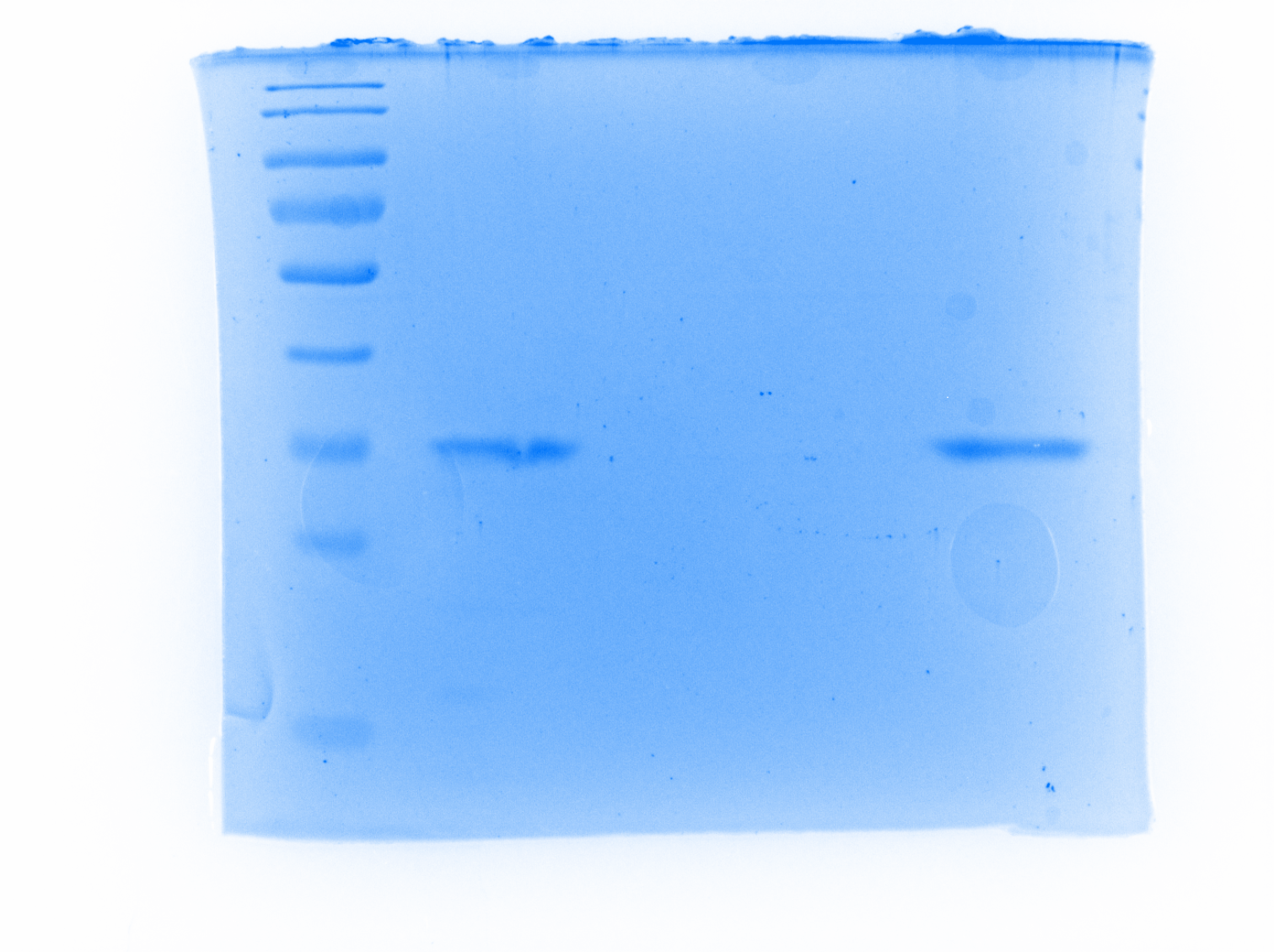

Supplement: Supplemental Information 2 [file peerj-10-12797-s002.zip › Figure-2/C-pull down lapf.tif]

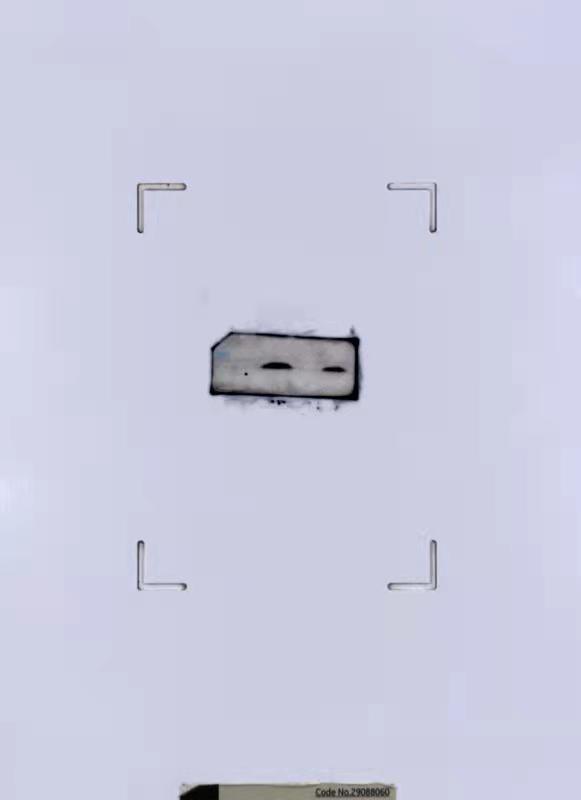

Supplement: Supplemental Information 2 [file peerj-10-12797-s002.zip › Figure-2/C-tgfwb.jpg]

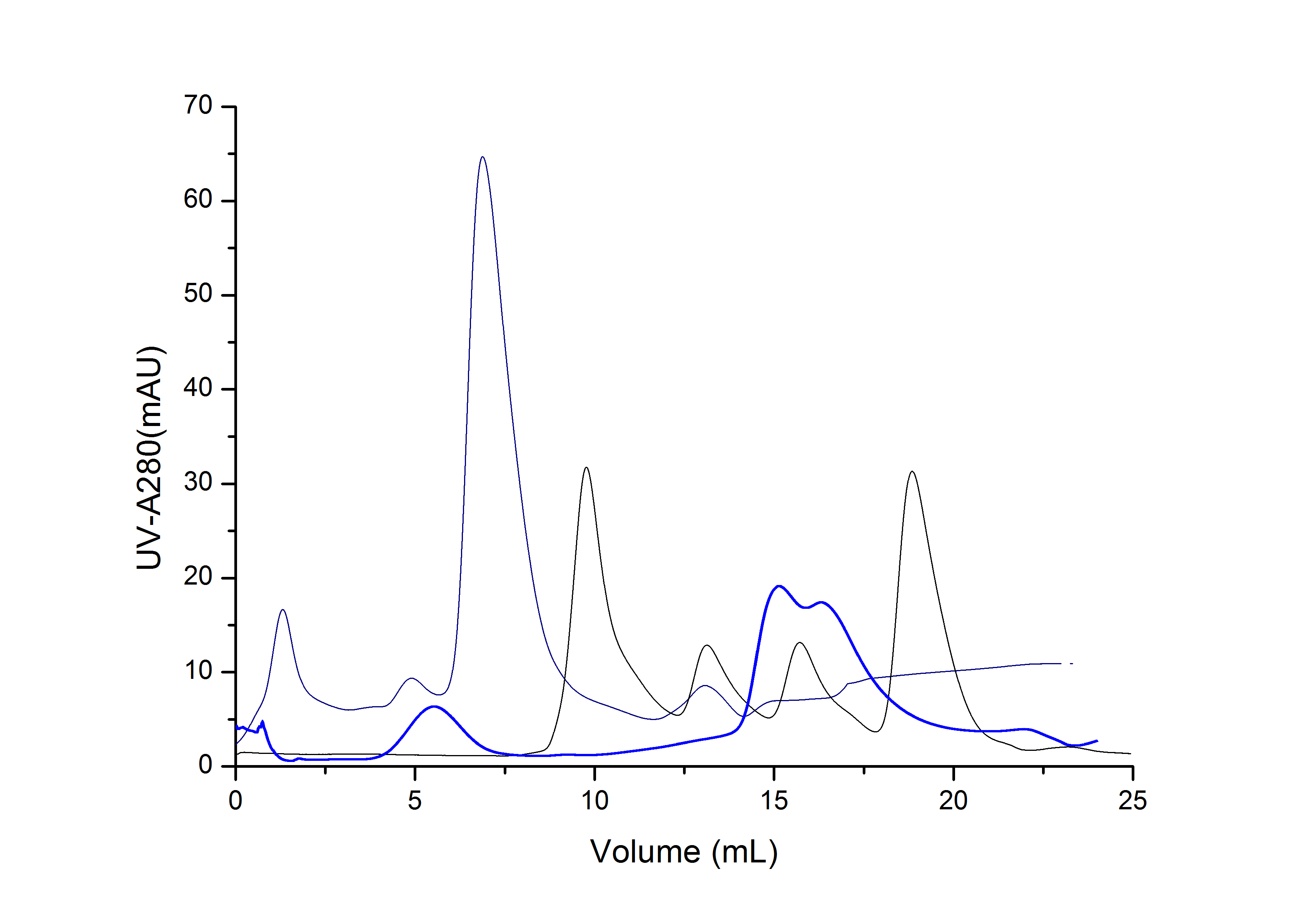

Supplement: Supplemental Information 2 [file peerj-10-12797-s002.zip › Figure-2/D-lap.jpg]

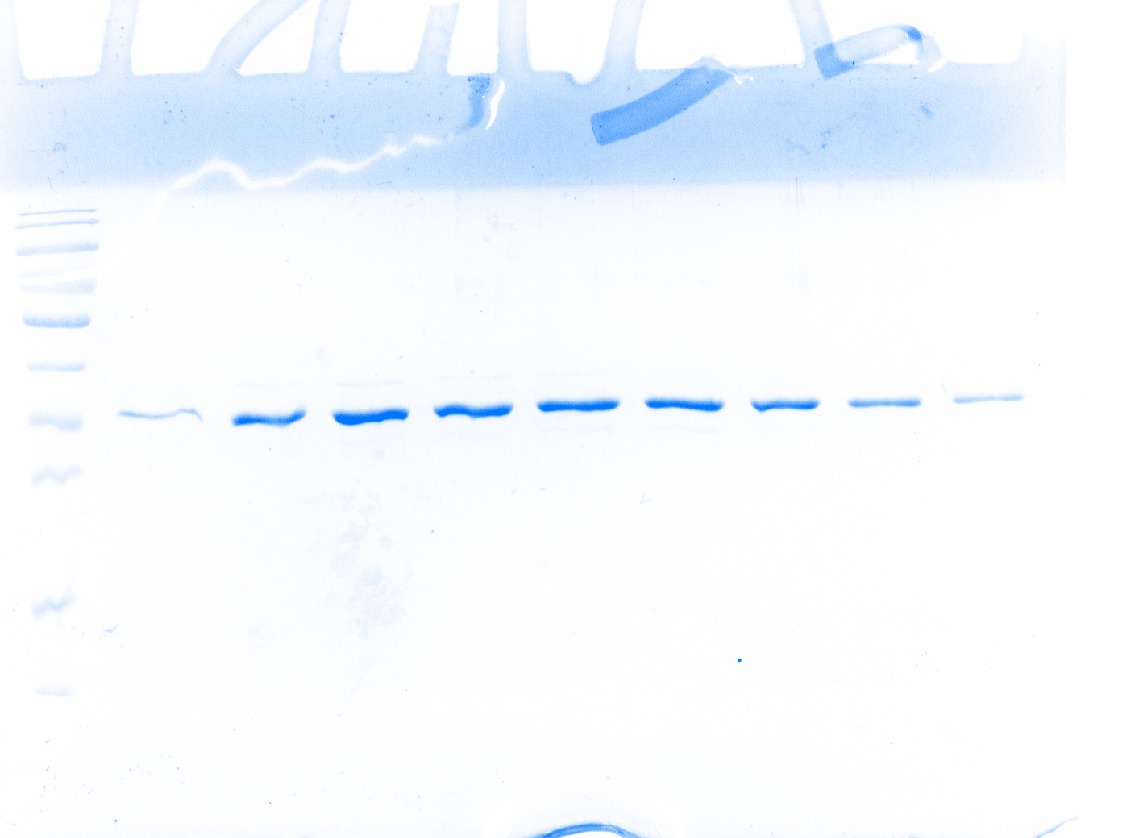

Supplement: Supplemental Information 2 [file peerj-10-12797-s002.zip › Figure-2/E1.tif]

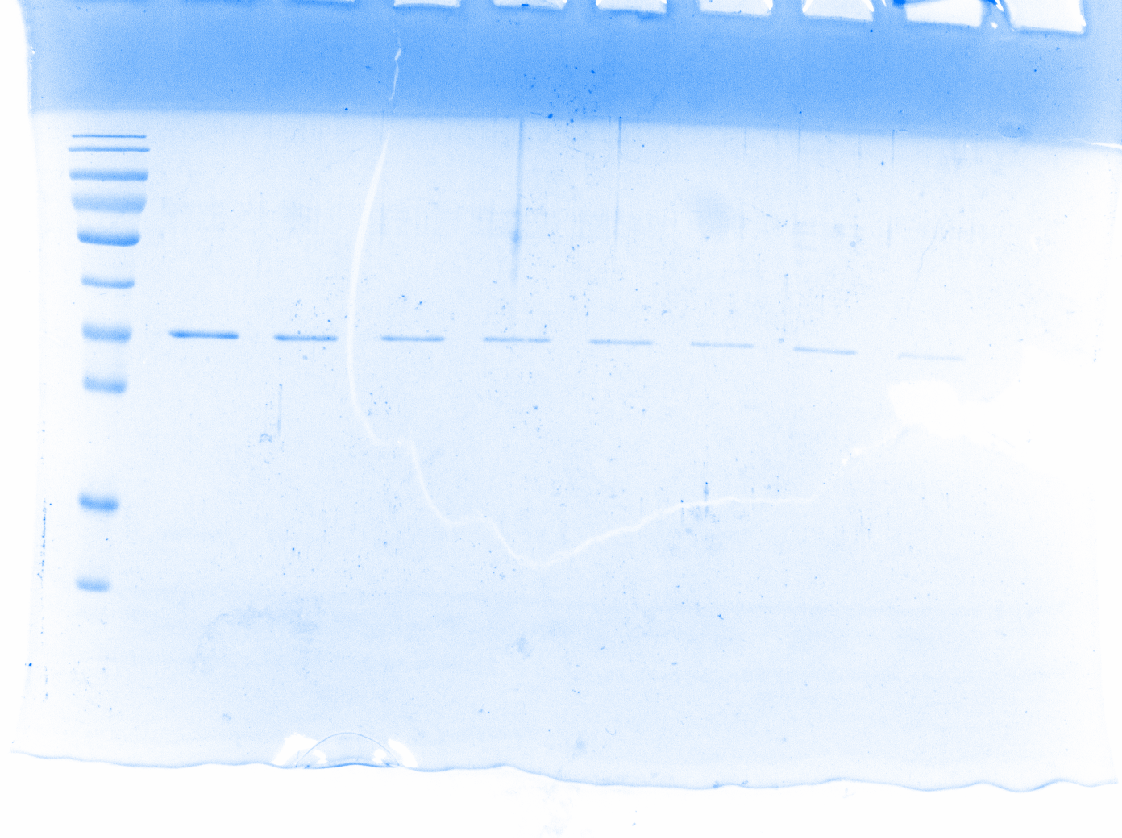

Supplement: Supplemental Information 2 [file peerj-10-12797-s002.zip › Figure-2/E2.tif]

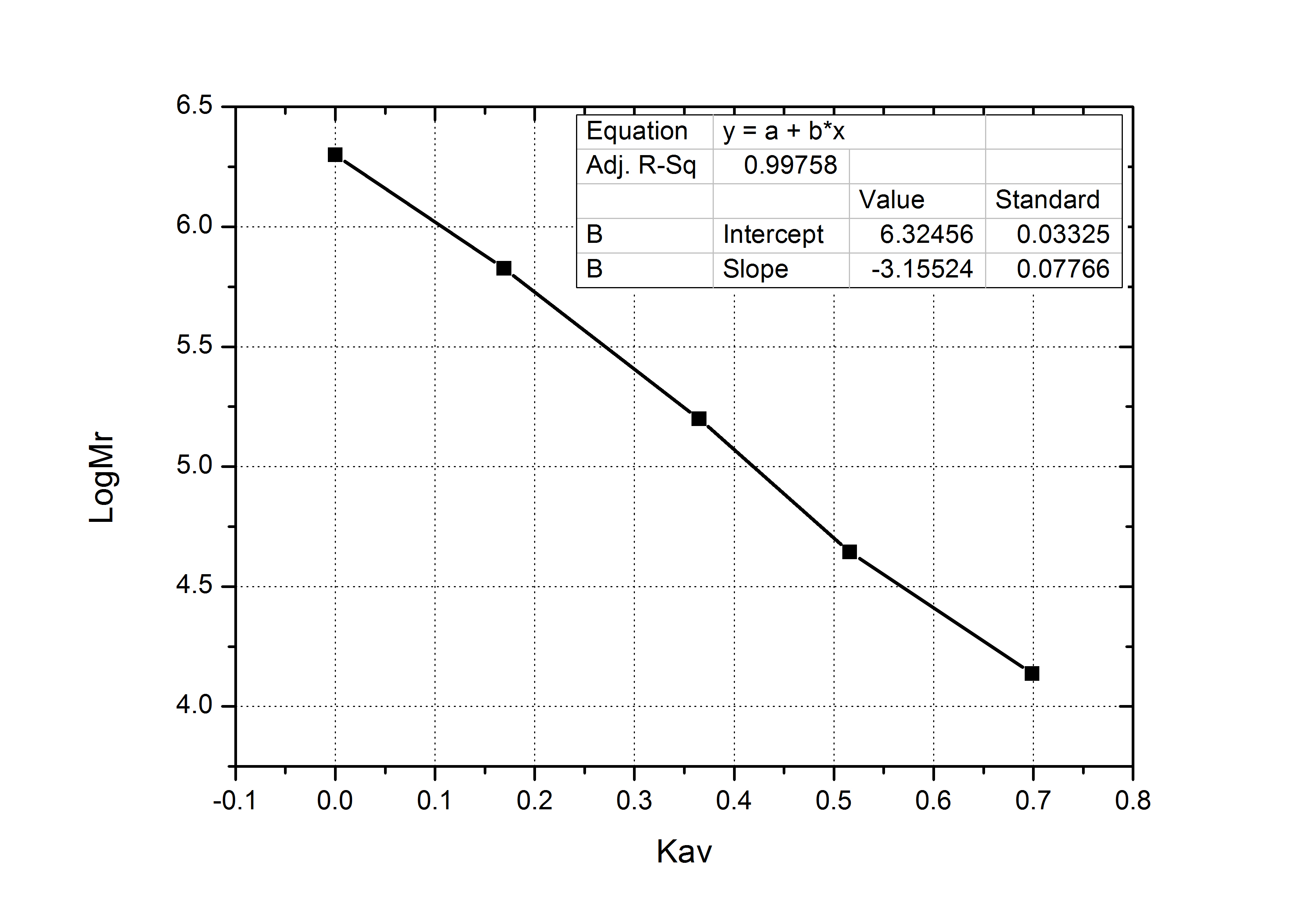

Supplement: Supplemental Information 2 [file peerj-10-12797-s002.zip › Figure-2/F.jpg]

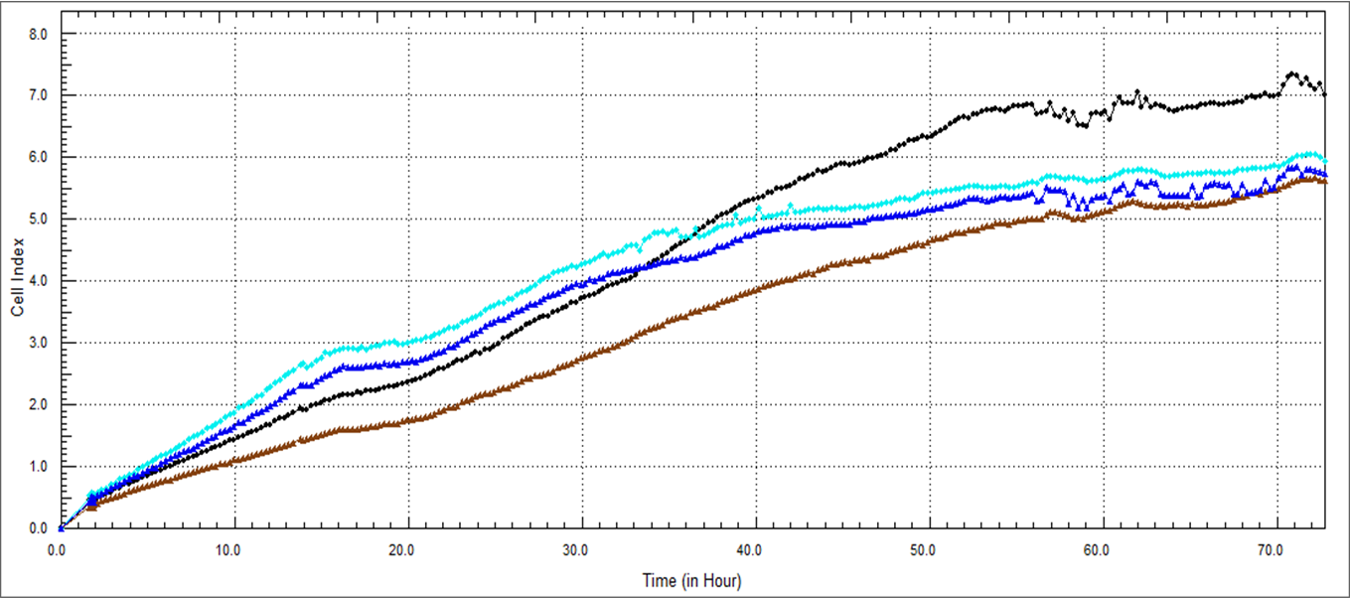

Supplement: Supplemental Information 3 [file peerj-10-12797-s003.zip › Figure 3/RTCA.png]

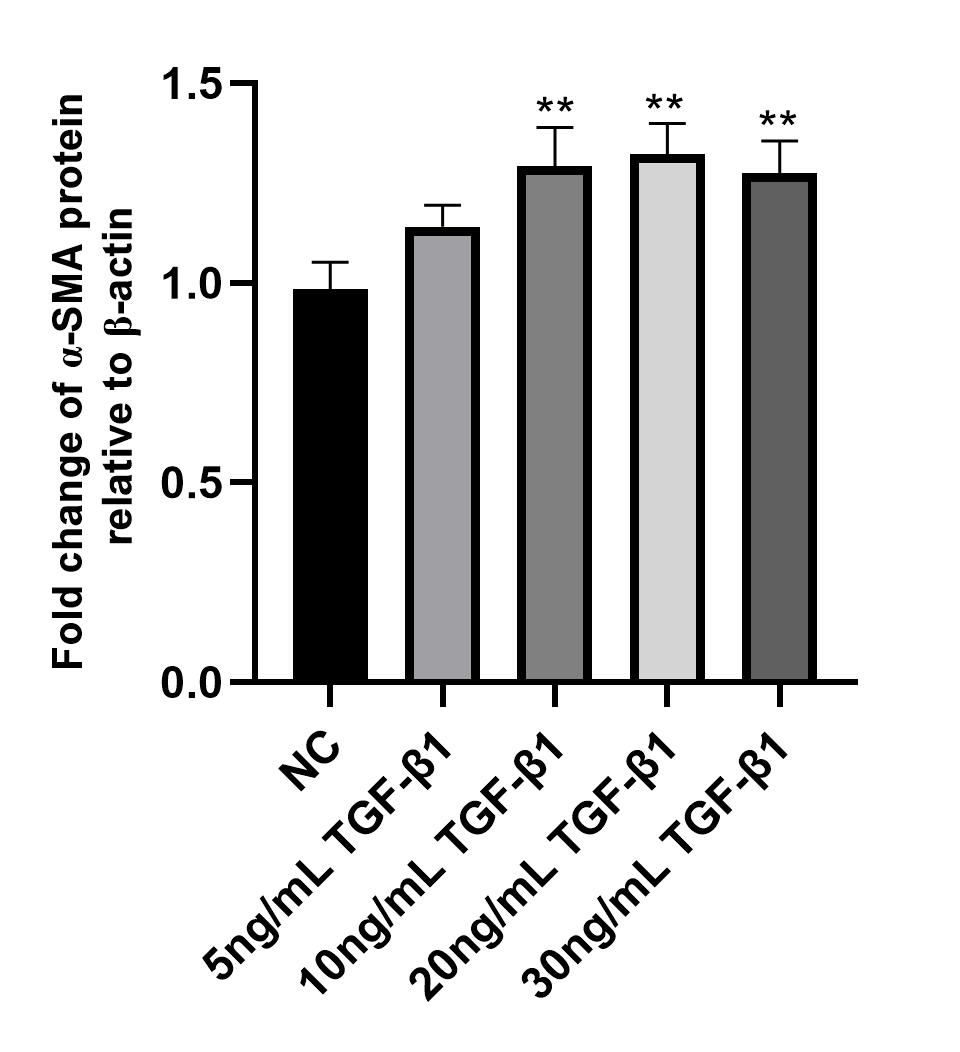

Supplement: Supplemental Information 3 [file peerj-10-12797-s003.zip › Figure 3/a-SMA.jpg]

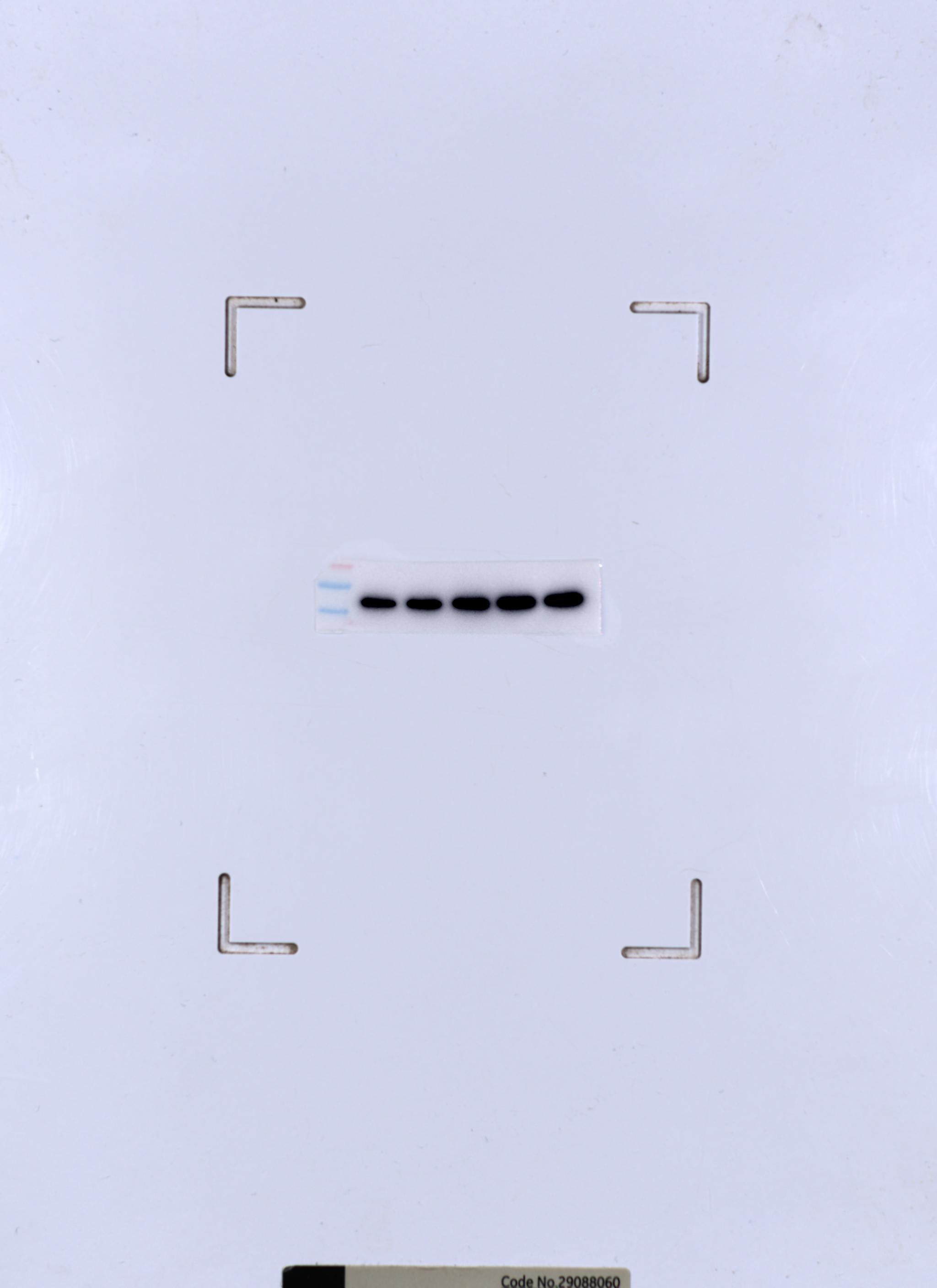

Supplement: Supplemental Information 3 [file peerj-10-12797-s003.zip › Figure 3/a┴-SMA/a┴-SMA #.jpg]

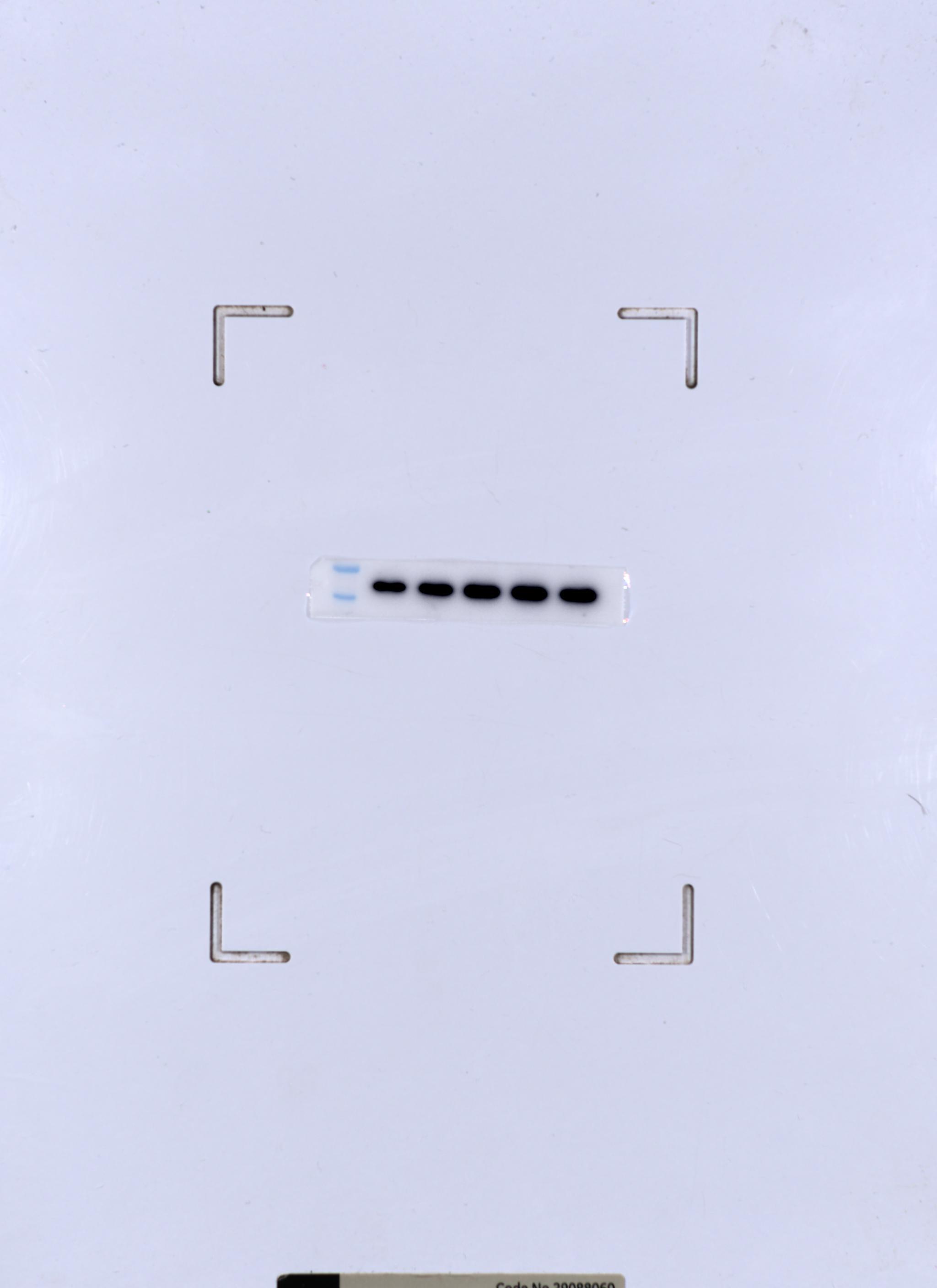

Supplement: Supplemental Information 3 [file peerj-10-12797-s003.zip › Figure 3/a┴-SMA/a┴-SMA 1.jpg]

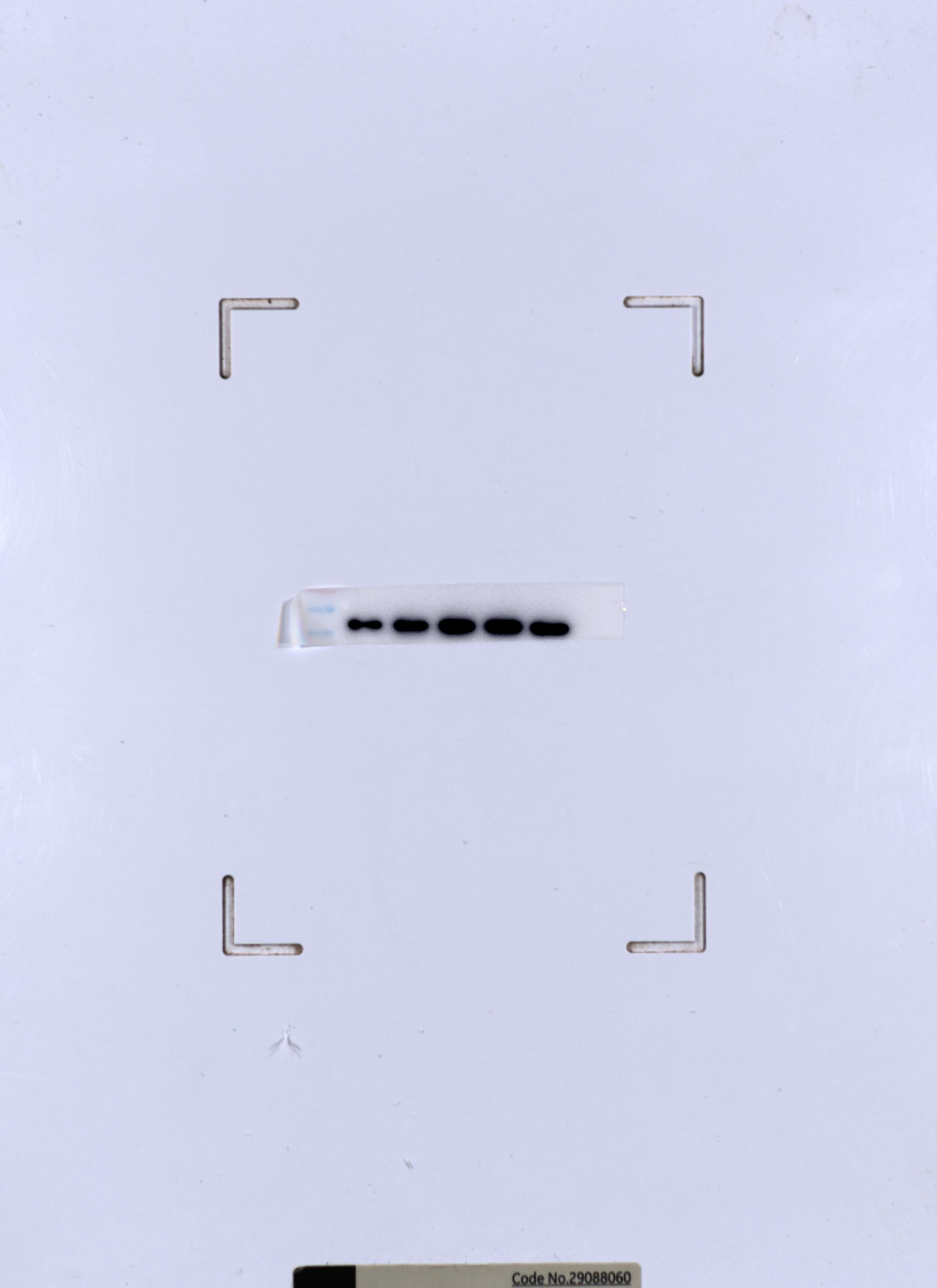

Supplement: Supplemental Information 3 [file peerj-10-12797-s003.zip › Figure 3/a┴-SMA/a┴-SMA 2.jpg]

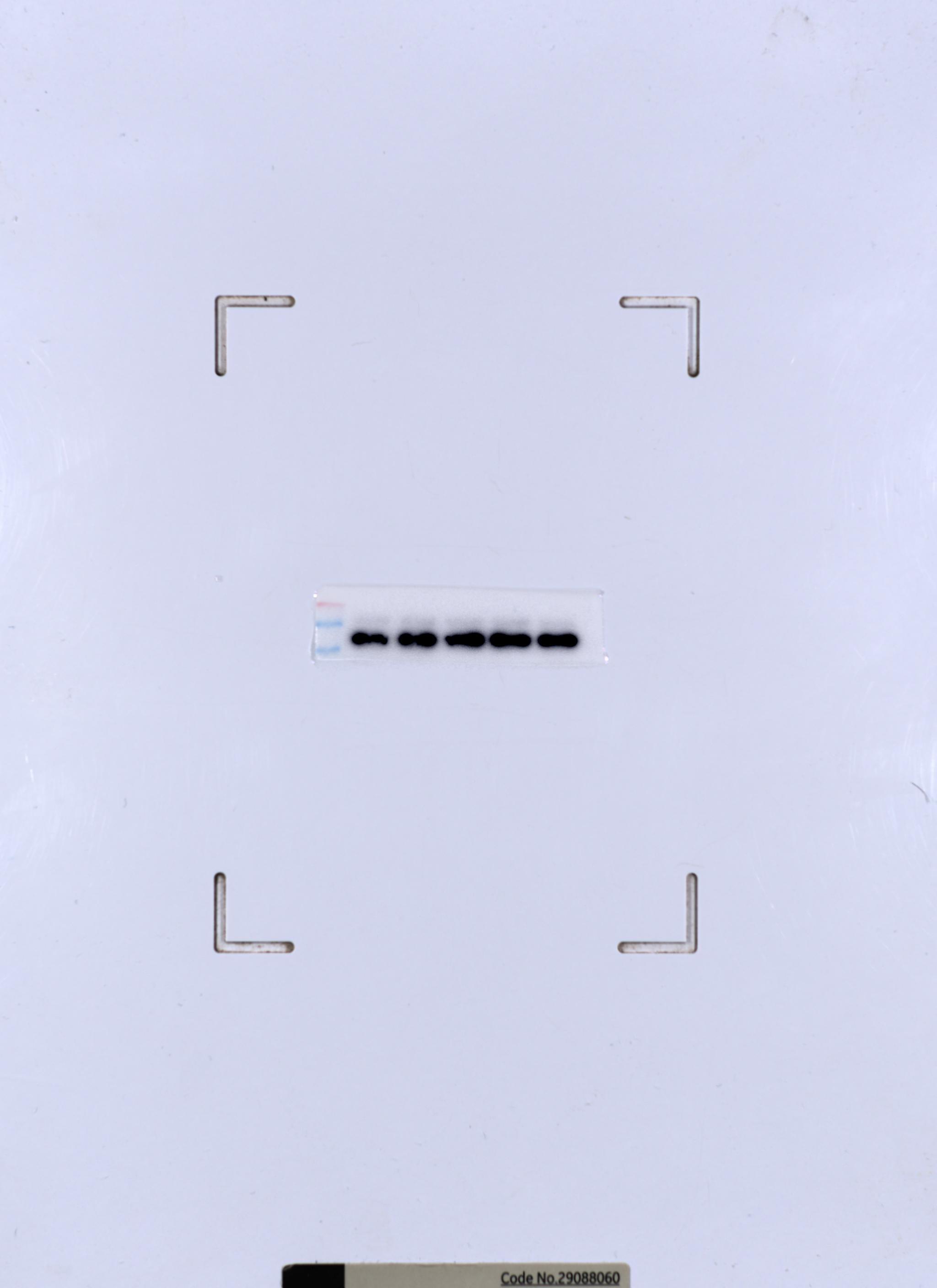

Supplement: Supplemental Information 3 [file peerj-10-12797-s003.zip › Figure 3/a┬-actin/a┬-actin #.jpg]

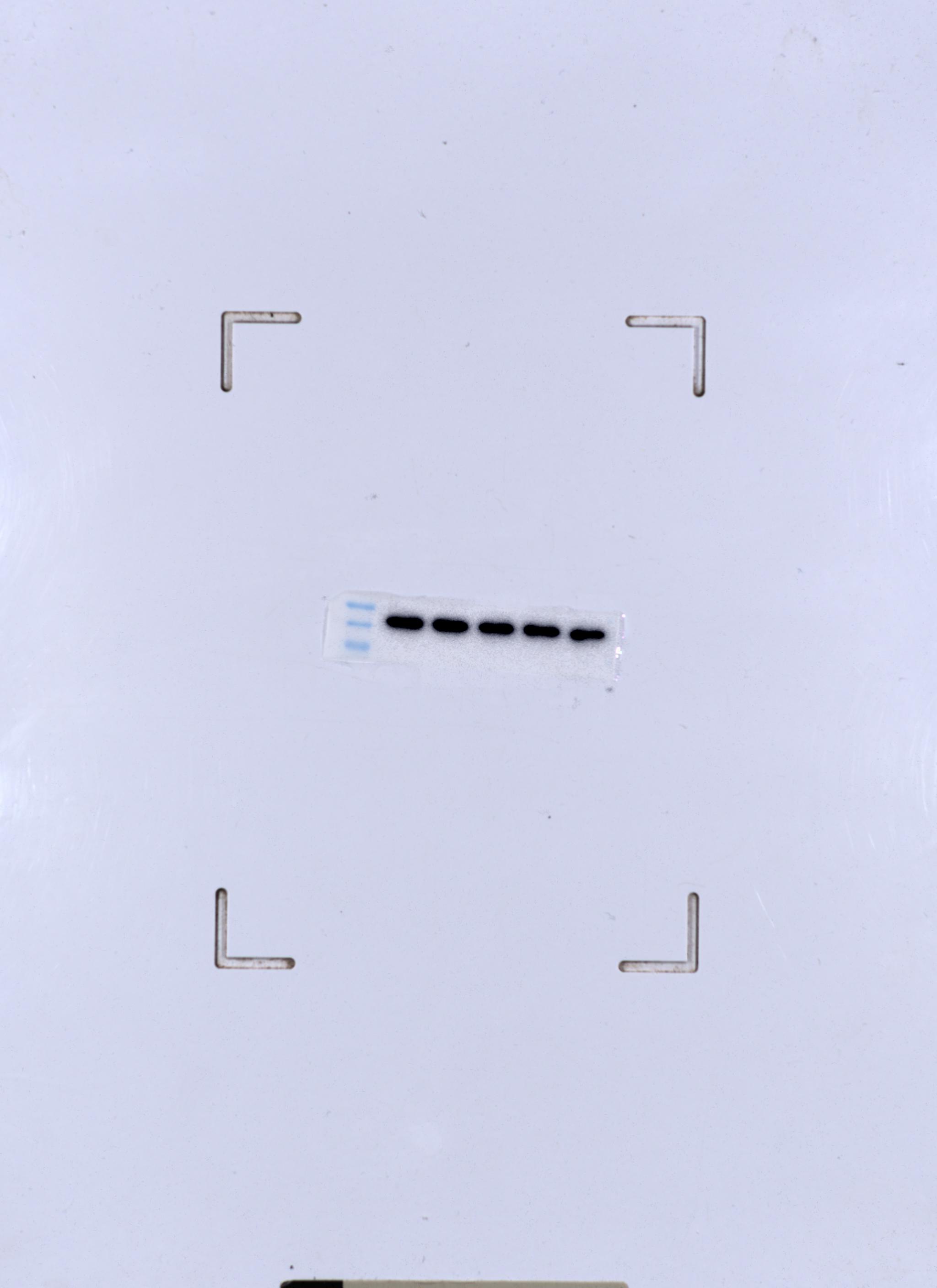

Supplement: Supplemental Information 3 [file peerj-10-12797-s003.zip › Figure 3/a┬-actin/a┬-actin 1.jpg]

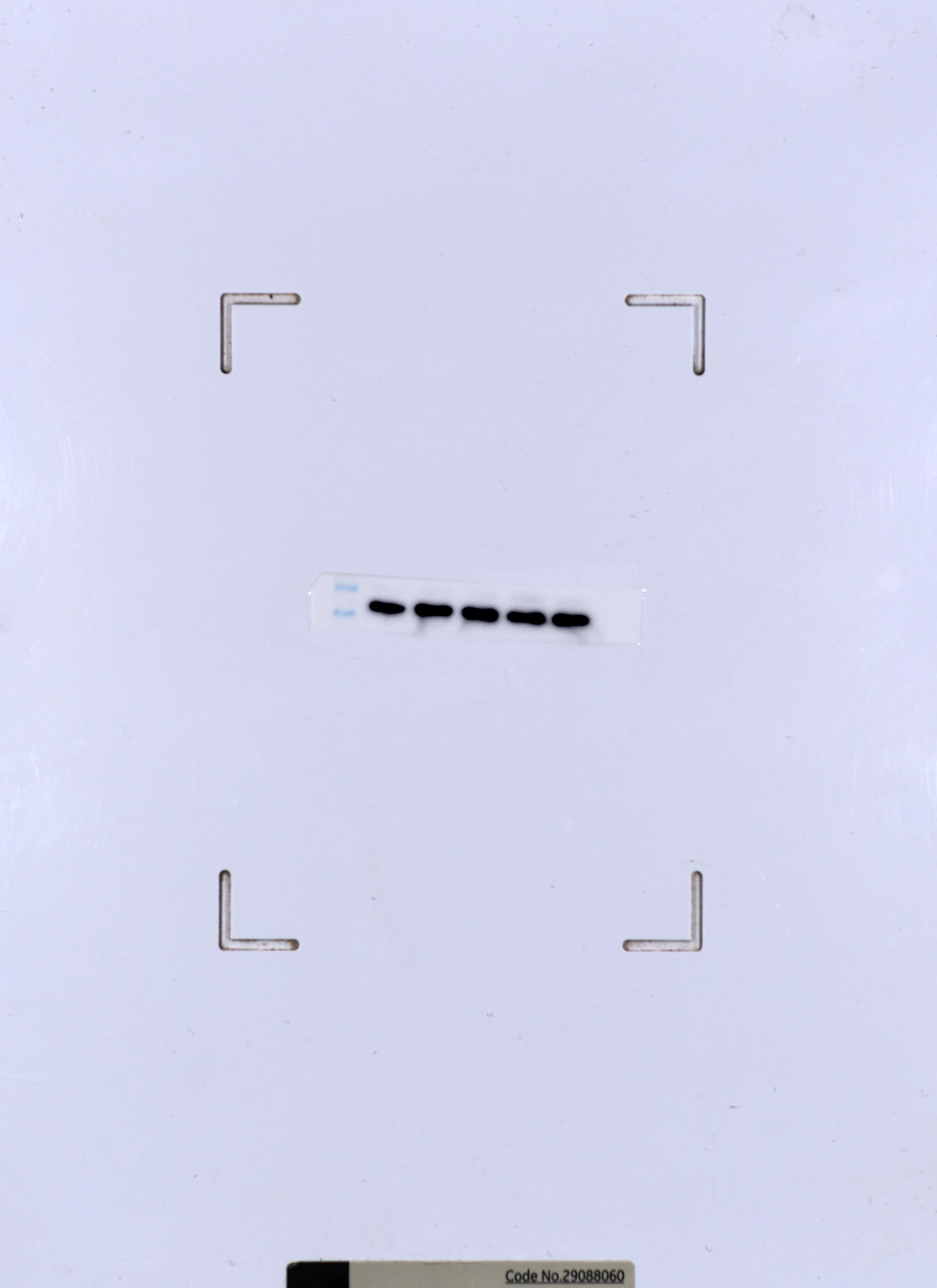

Supplement: Supplemental Information 3 [file peerj-10-12797-s003.zip › Figure 3/a┬-actin/a┬-actin 2.jpg]

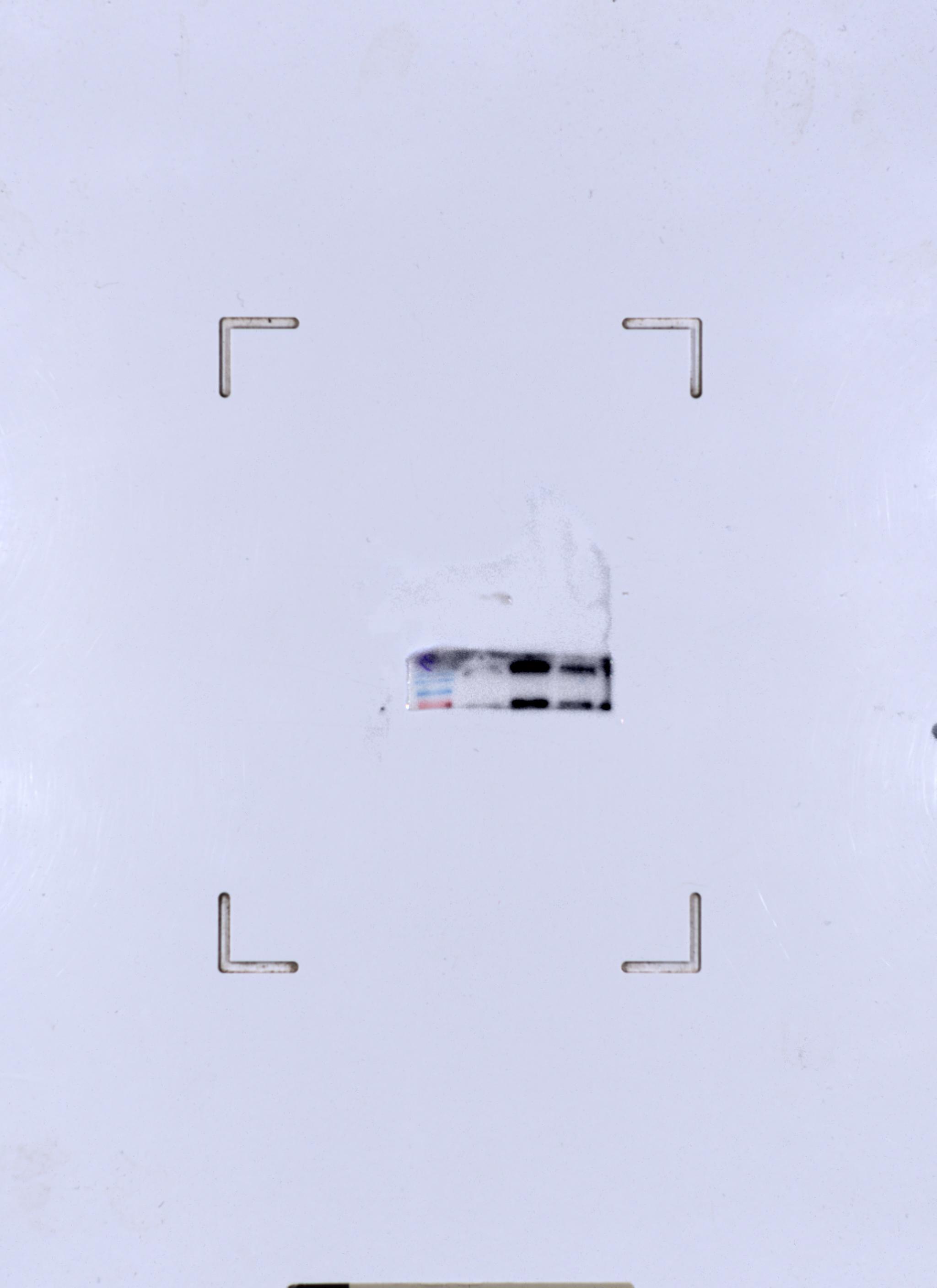

Supplement: Supplemental Information 4 [file peerj-10-12797-s004.zip › Figure 4/COL 1/COL1 #.jpg]

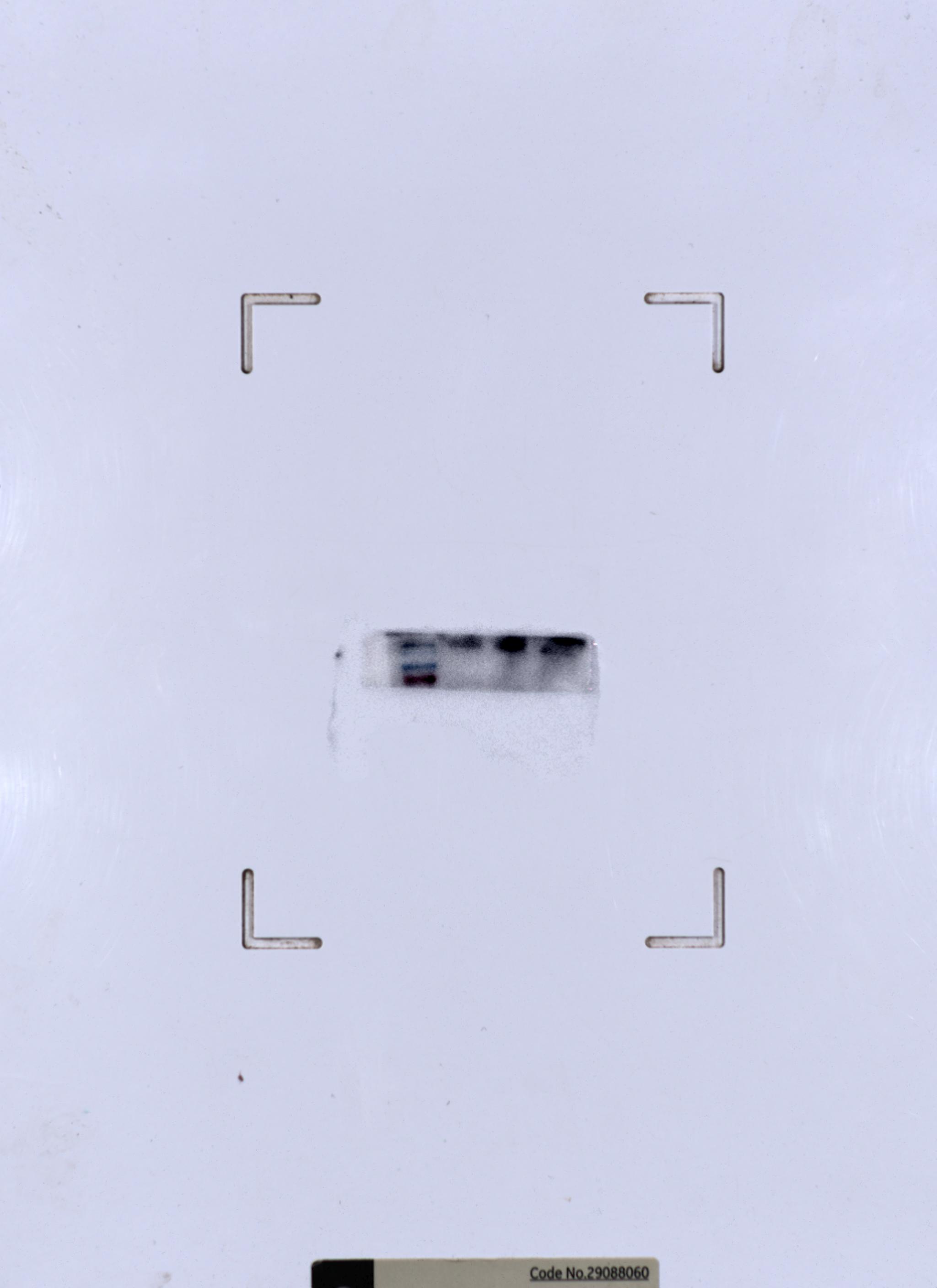

Supplement: Supplemental Information 4 [file peerj-10-12797-s004.zip › Figure 4/COL 1/COL1 1.jpg]

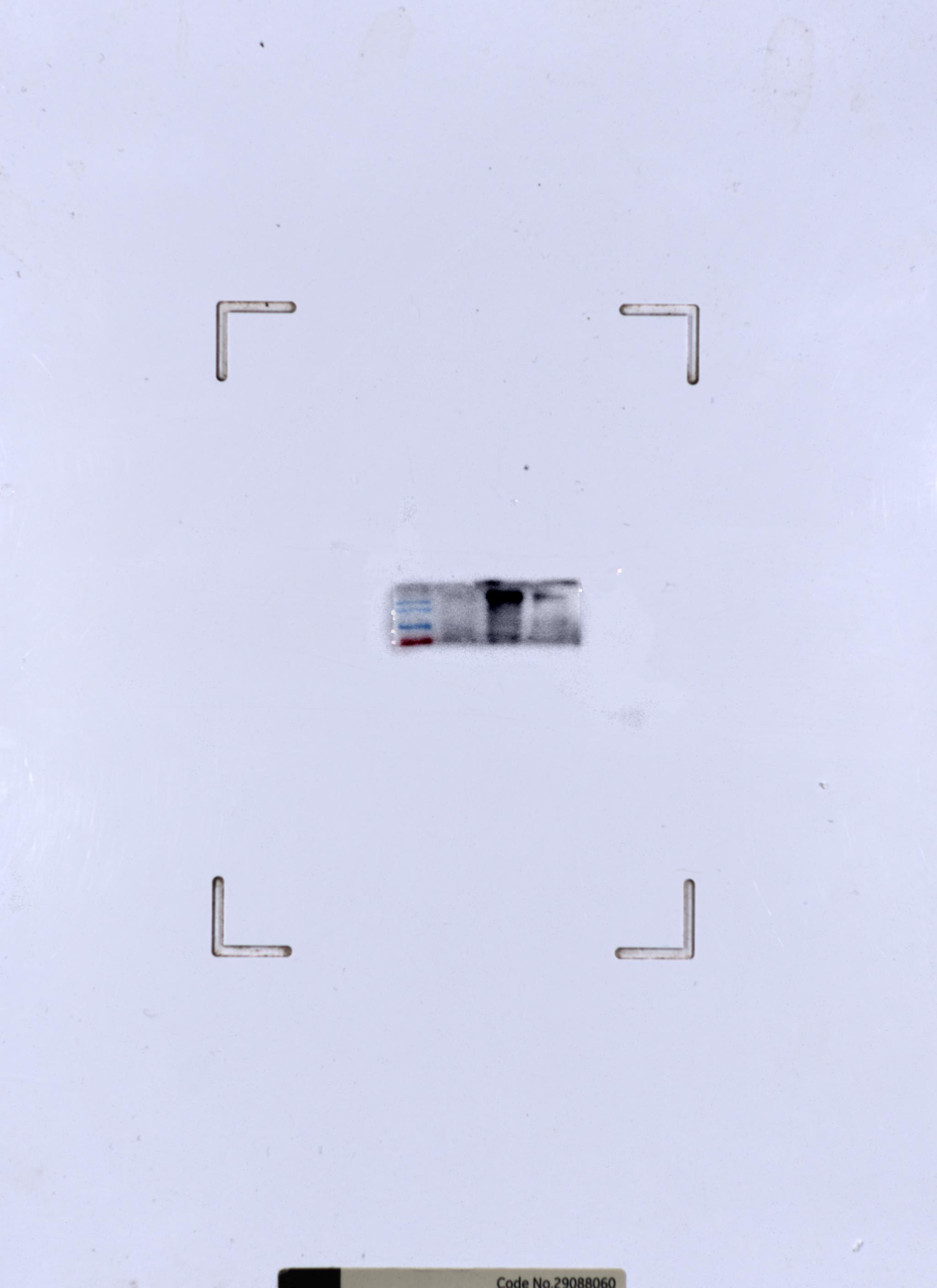

Supplement: Supplemental Information 4 [file peerj-10-12797-s004.zip › Figure 4/COL 1/COL1 2.jpg]

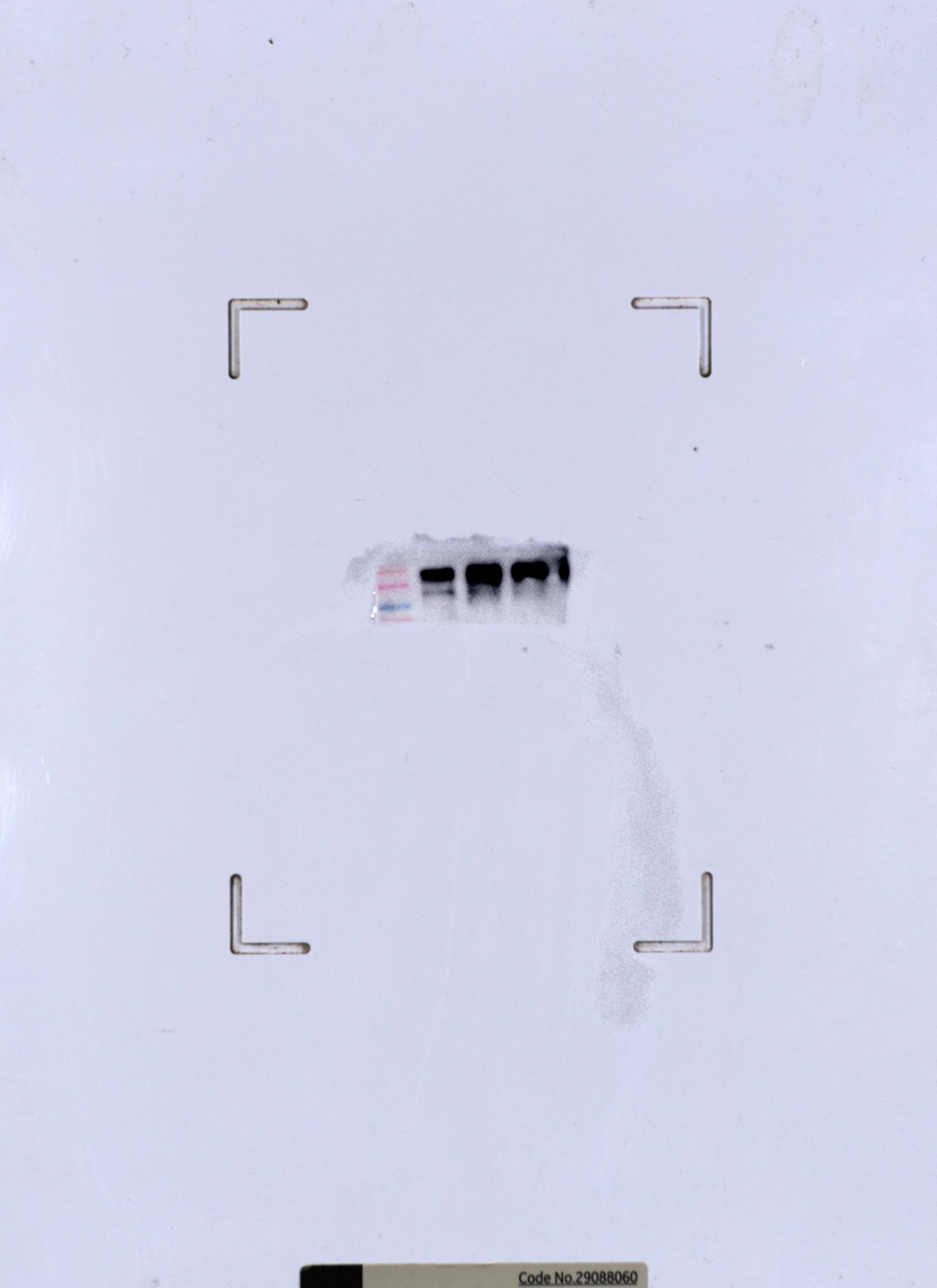

Supplement: Supplemental Information 4 [file peerj-10-12797-s004.zip › Figure 4/FN/FN #.jpg]

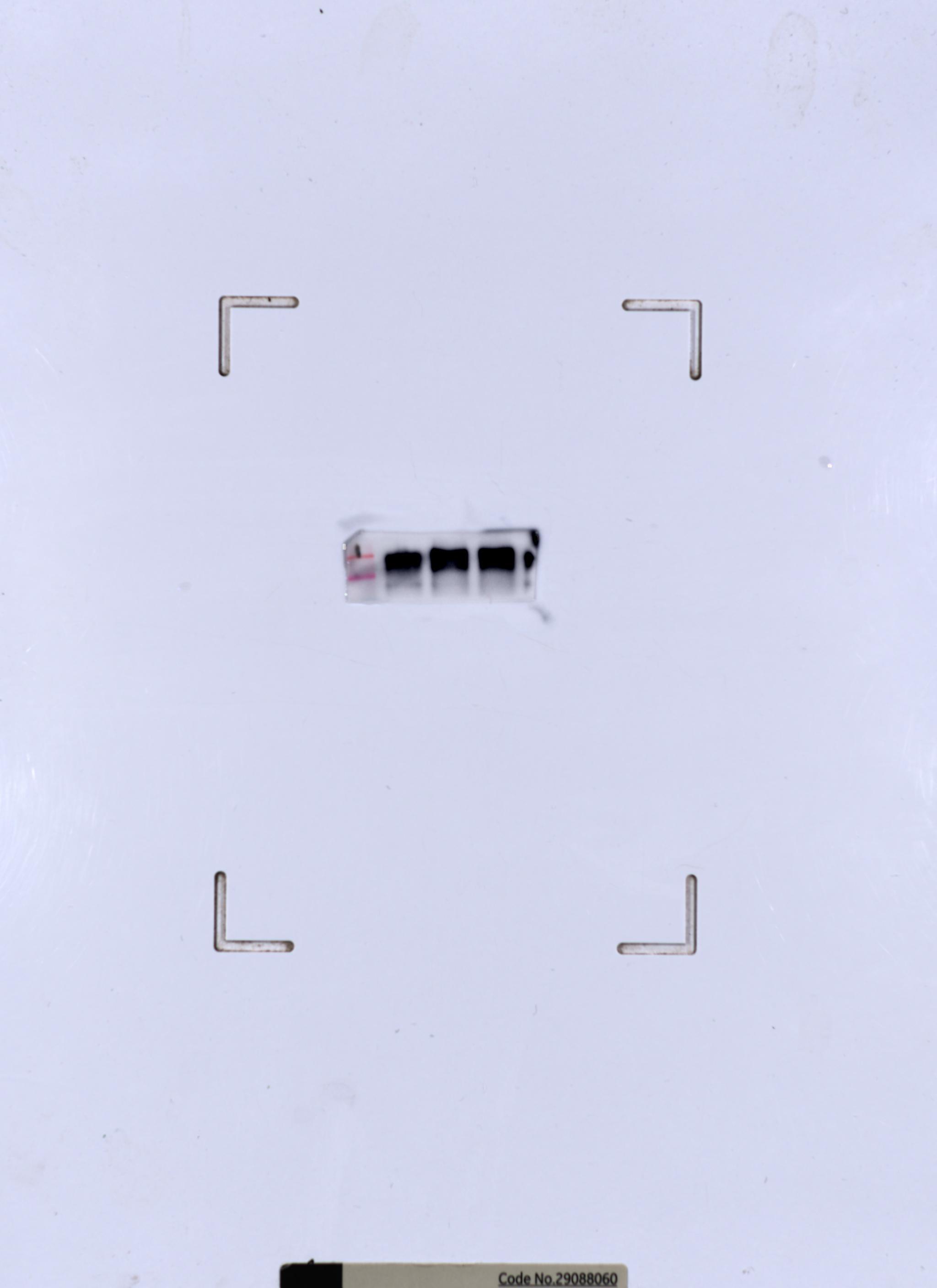

Supplement: Supplemental Information 4 [file peerj-10-12797-s004.zip › Figure 4/FN/FN 1.jpg]

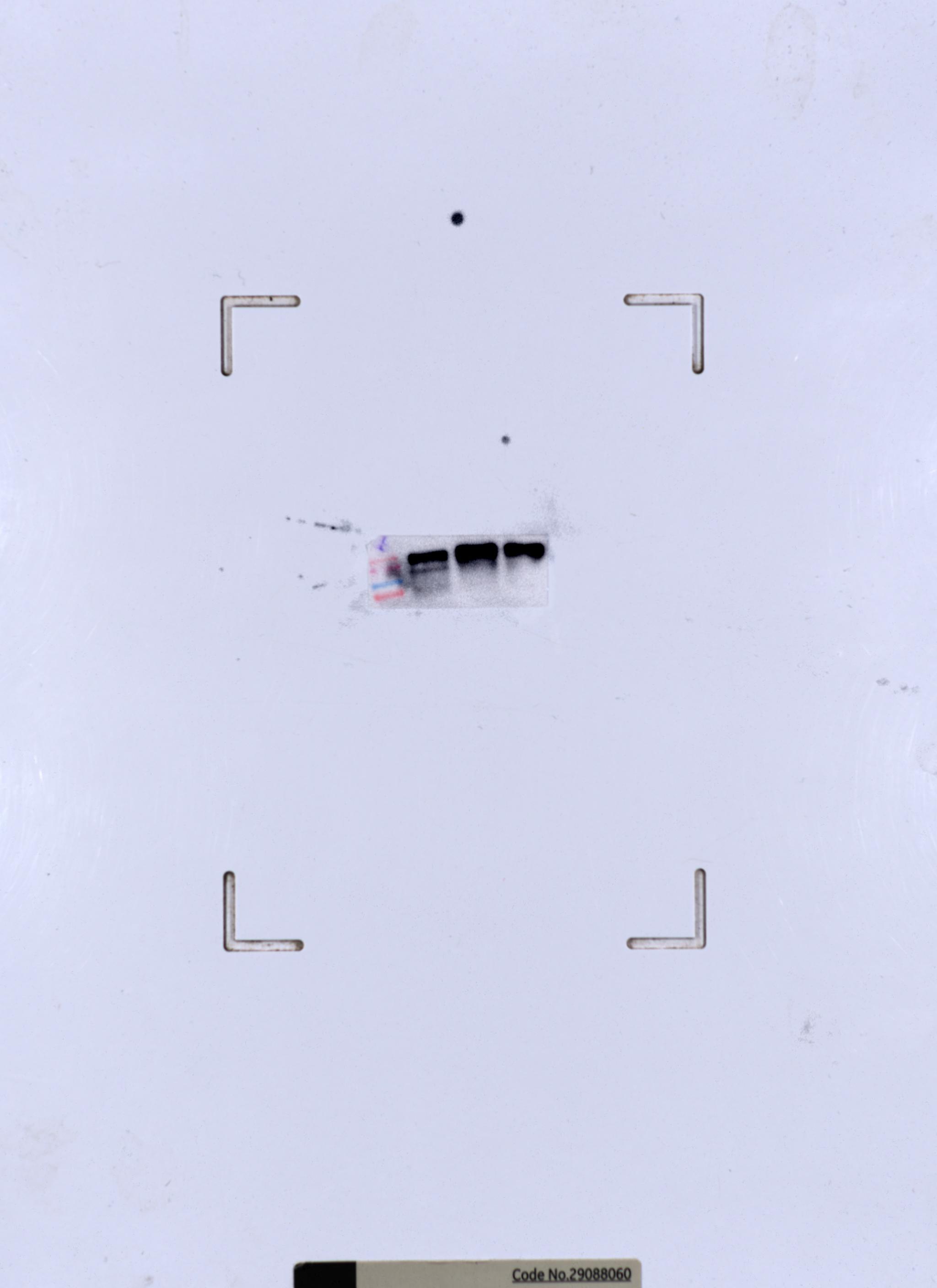

Supplement: Supplemental Information 4 [file peerj-10-12797-s004.zip › Figure 4/FN/FN 2.jpg]

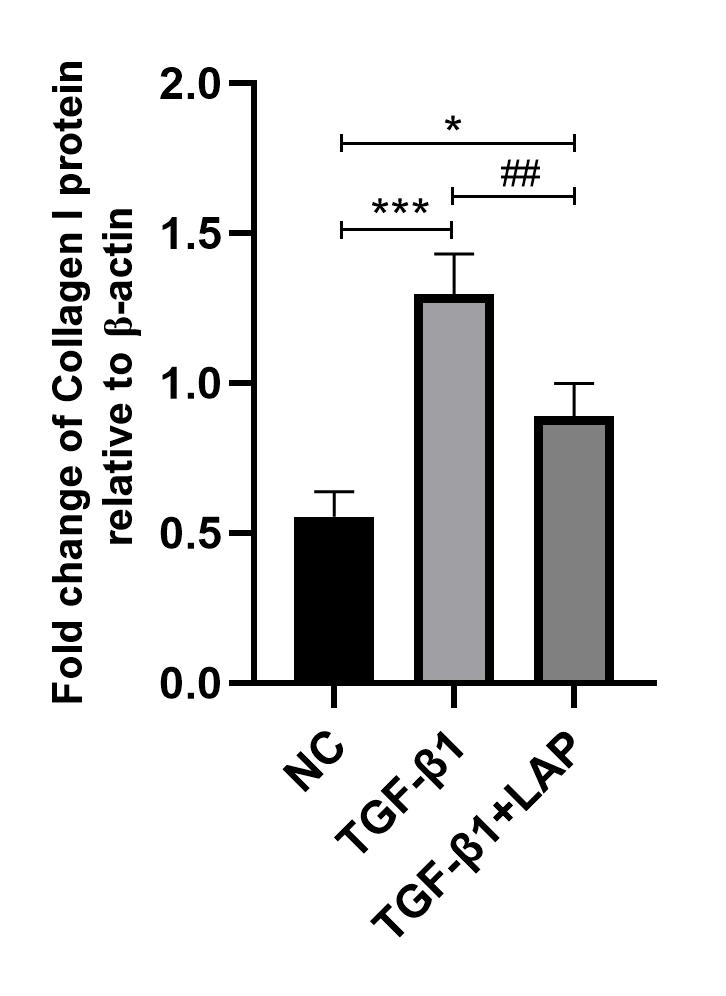

Supplement: Supplemental Information 4 [file peerj-10-12797-s004.zip › Figure 4/Fig 4 COL 1.jpg]

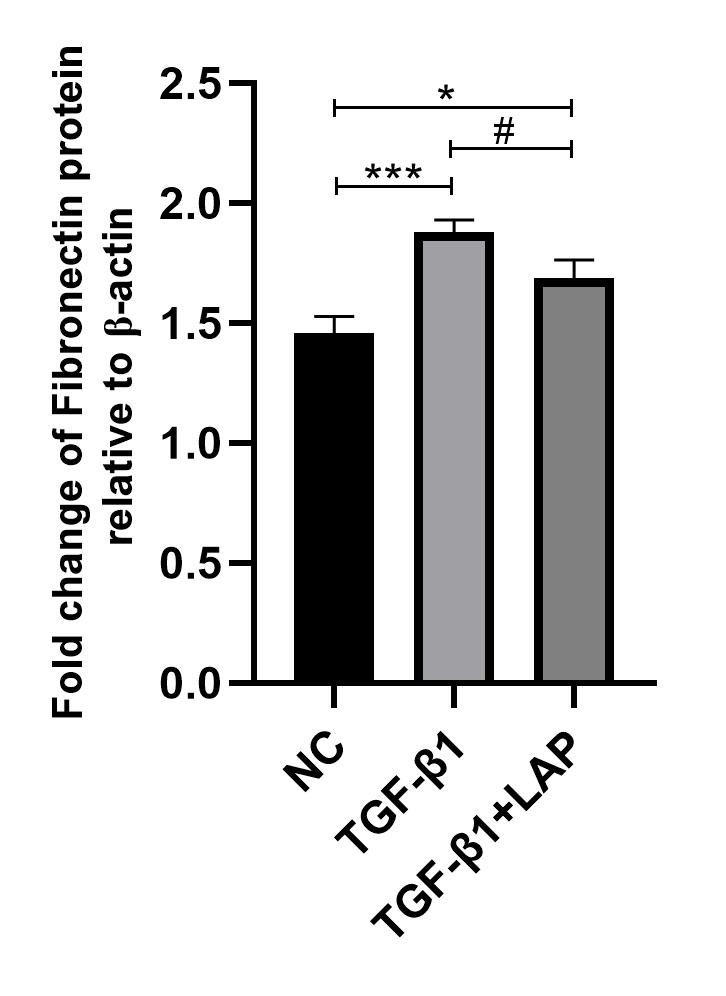

Supplement: Supplemental Information 4 [file peerj-10-12797-s004.zip › Figure 4/Fig 4 FN.jpg]

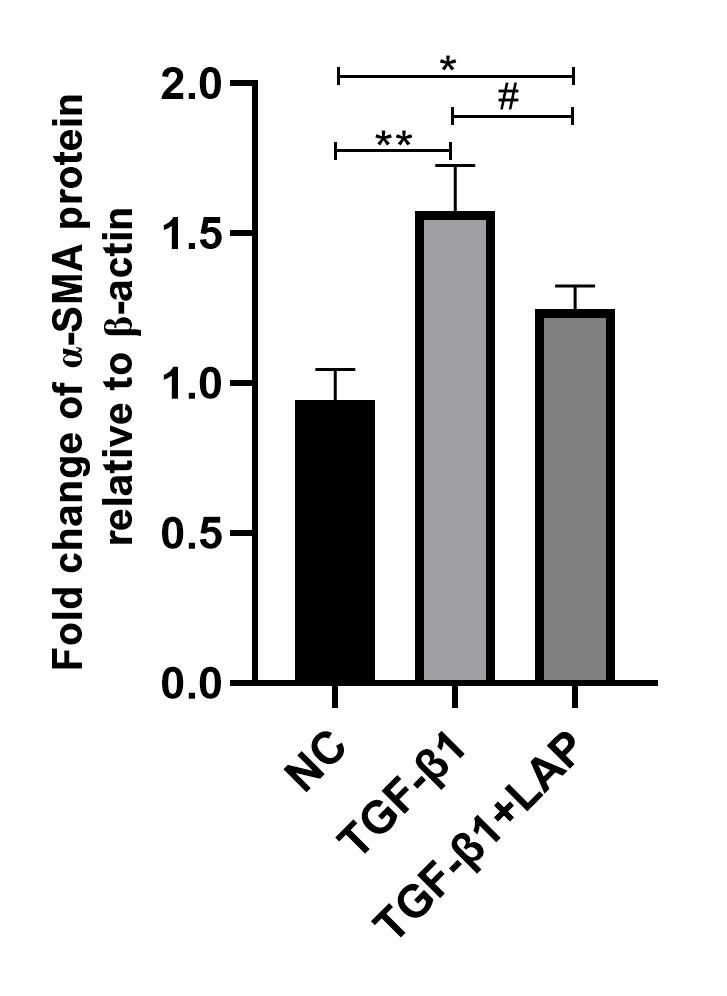

Supplement: Supplemental Information 4 [file peerj-10-12797-s004.zip › Figure 4/Fig 4 a┴-SMA.jpg]

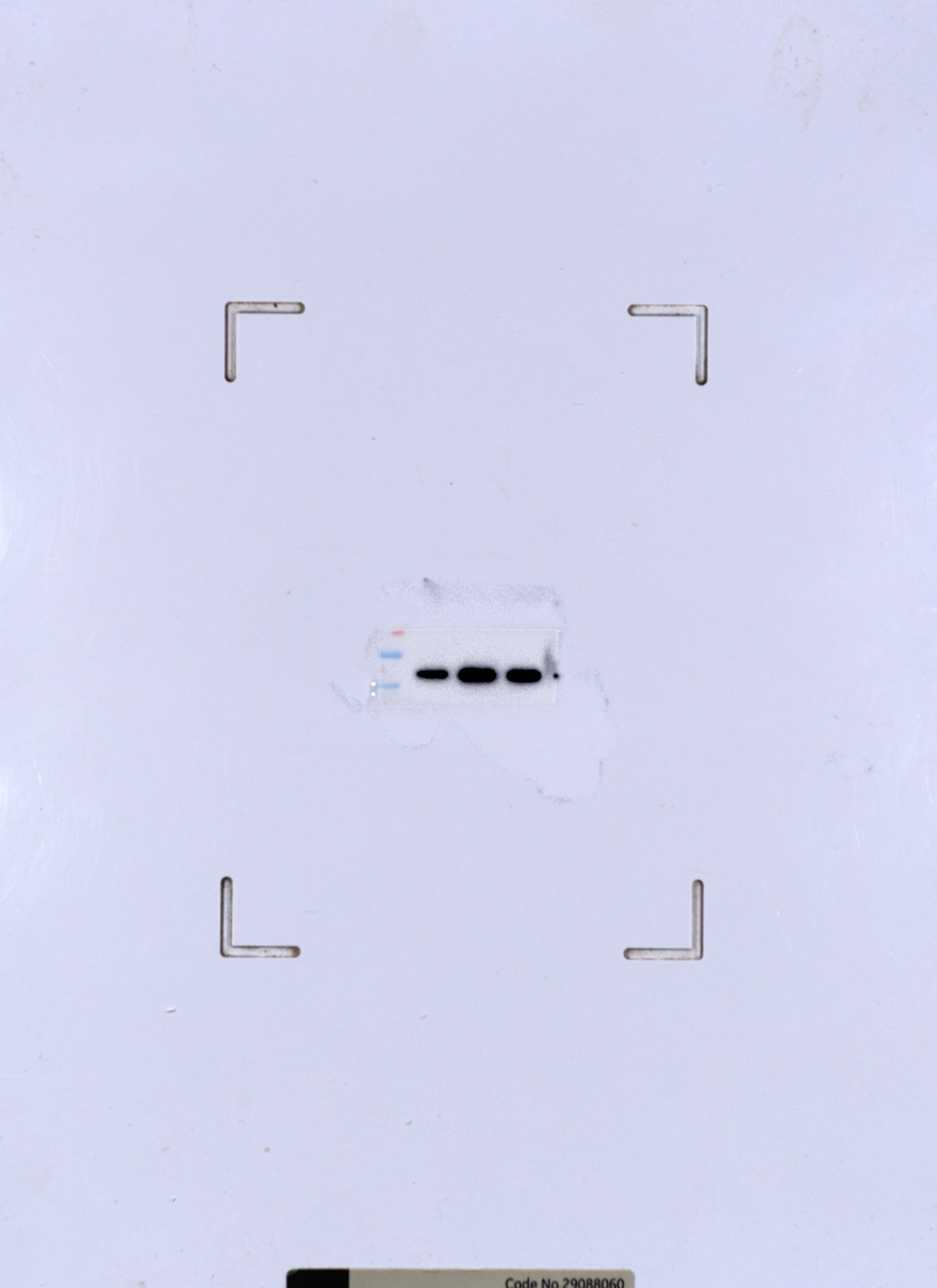

Supplement: Supplemental Information 4 [file peerj-10-12797-s004.zip › Figure 4/a┴-SMA/a┴-SMA #.jpg]

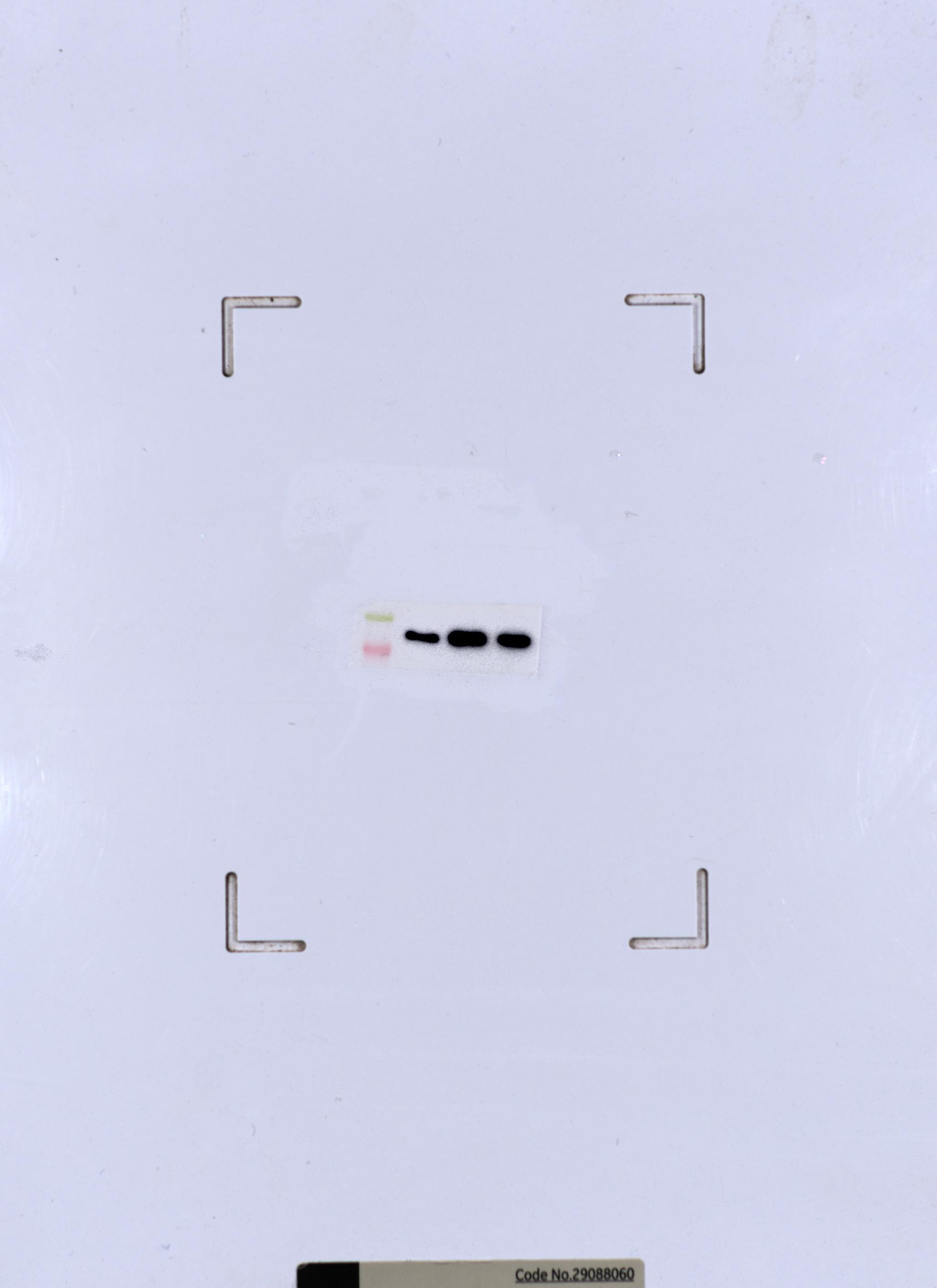

Supplement: Supplemental Information 4 [file peerj-10-12797-s004.zip › Figure 4/a┴-SMA/a┴-SMA1.jpg]

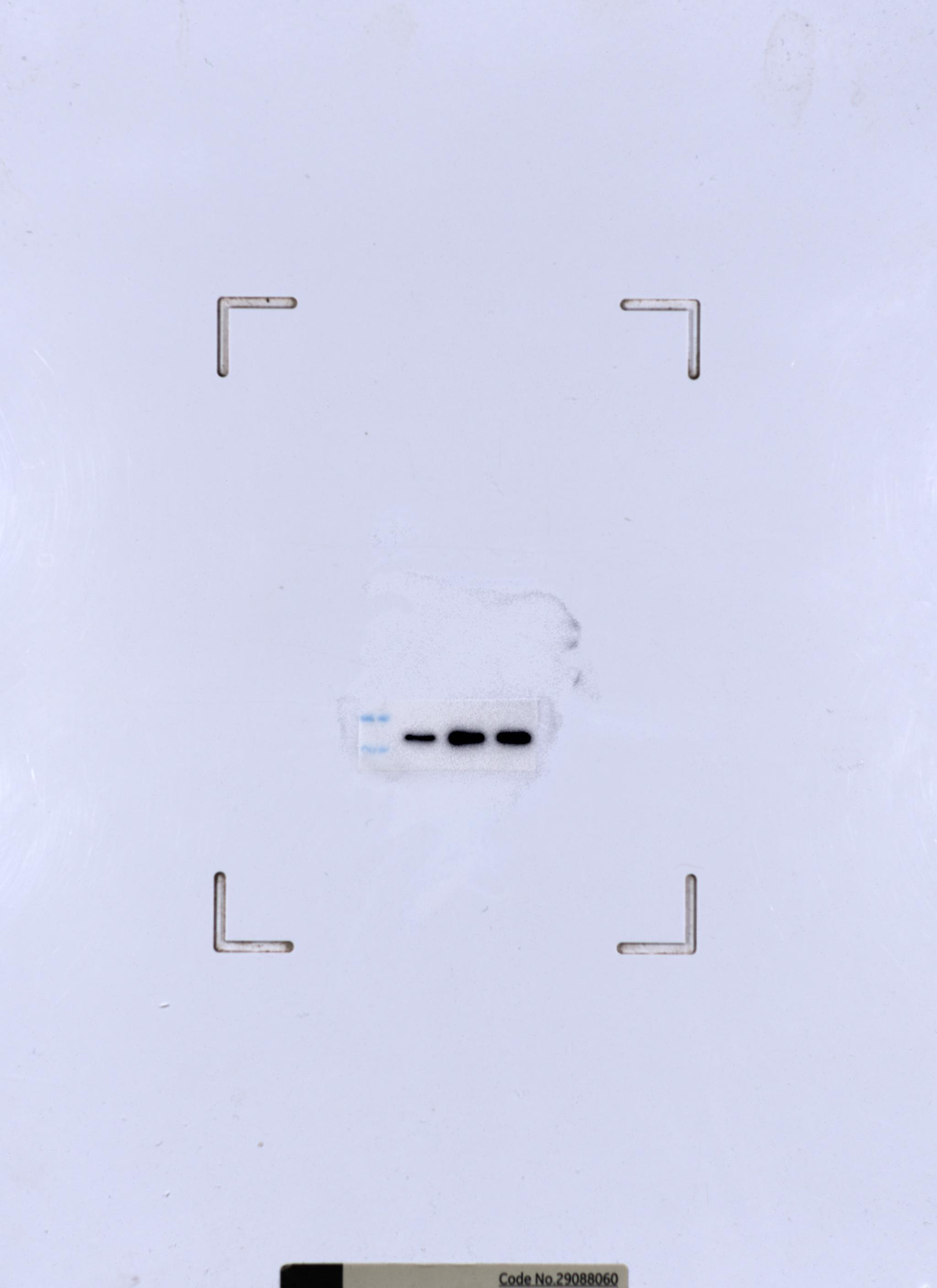

Supplement: Supplemental Information 4 [file peerj-10-12797-s004.zip › Figure 4/a┴-SMA/a┴-SMA2.jpg]

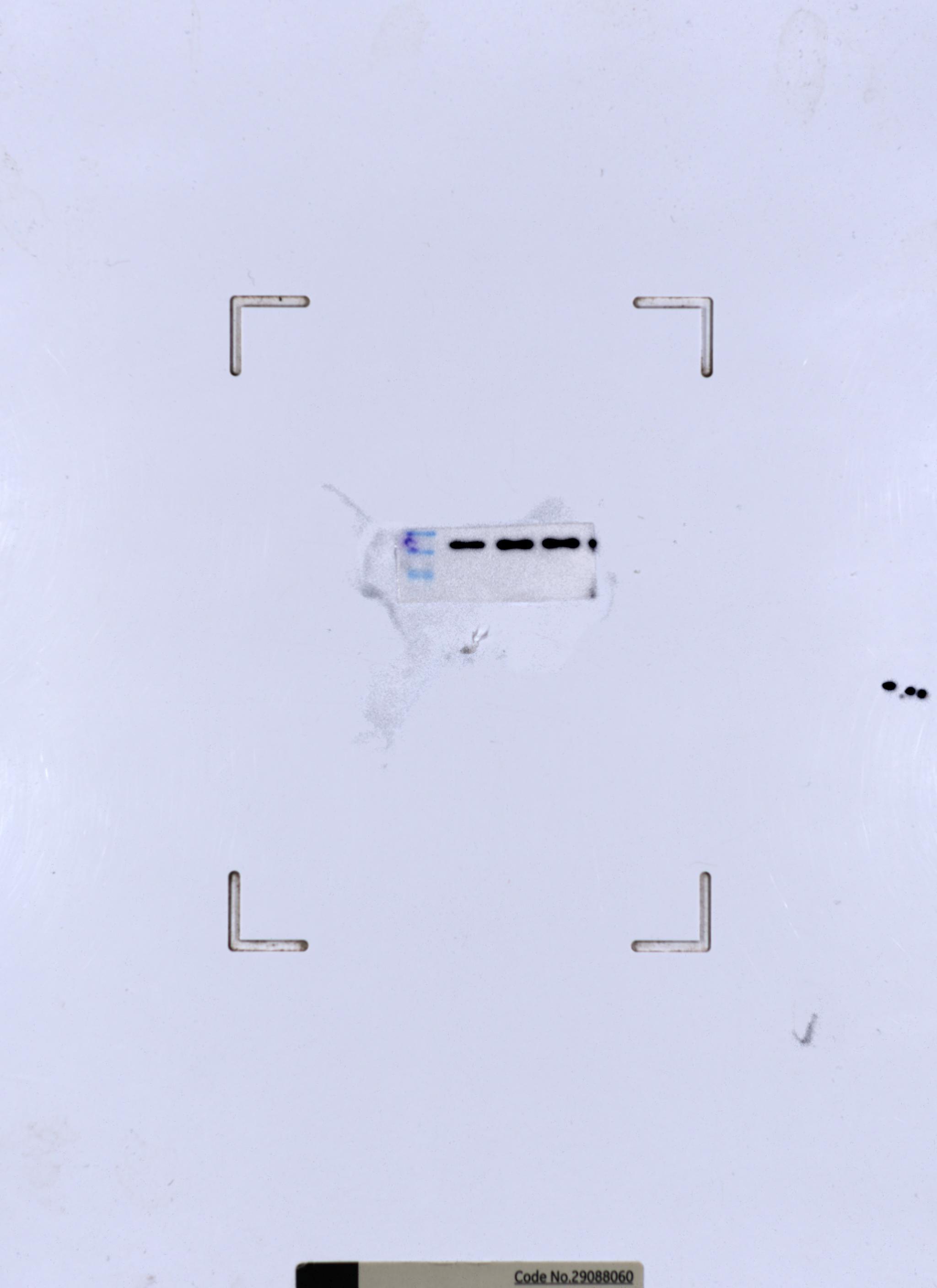

Supplement: Supplemental Information 4 [file peerj-10-12797-s004.zip › Figure 4/a┬-actin/a┬-actin#.jpg]

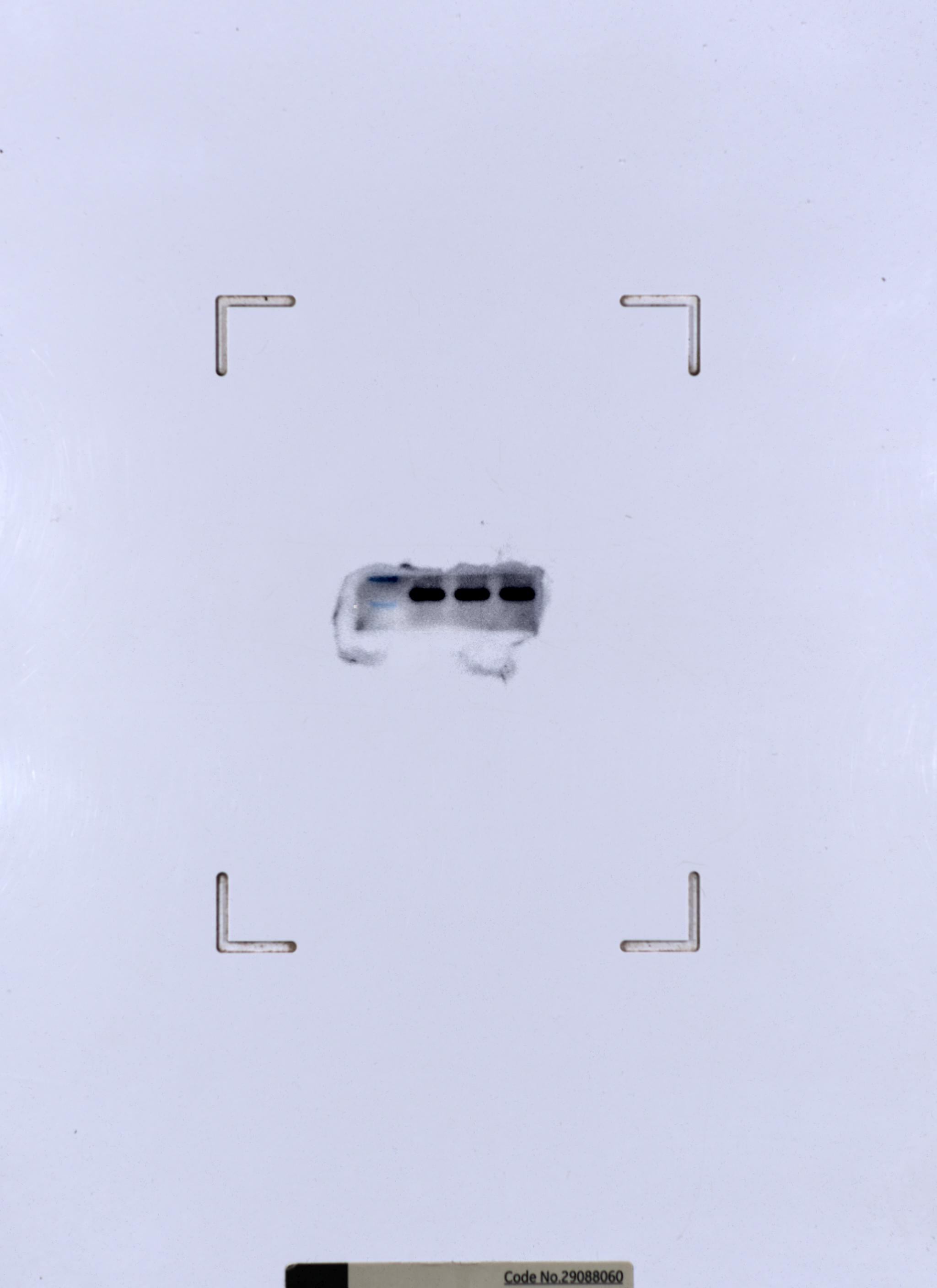

Supplement: Supplemental Information 4 [file peerj-10-12797-s004.zip › Figure 4/a┬-actin/a┬-actin1.jpg]

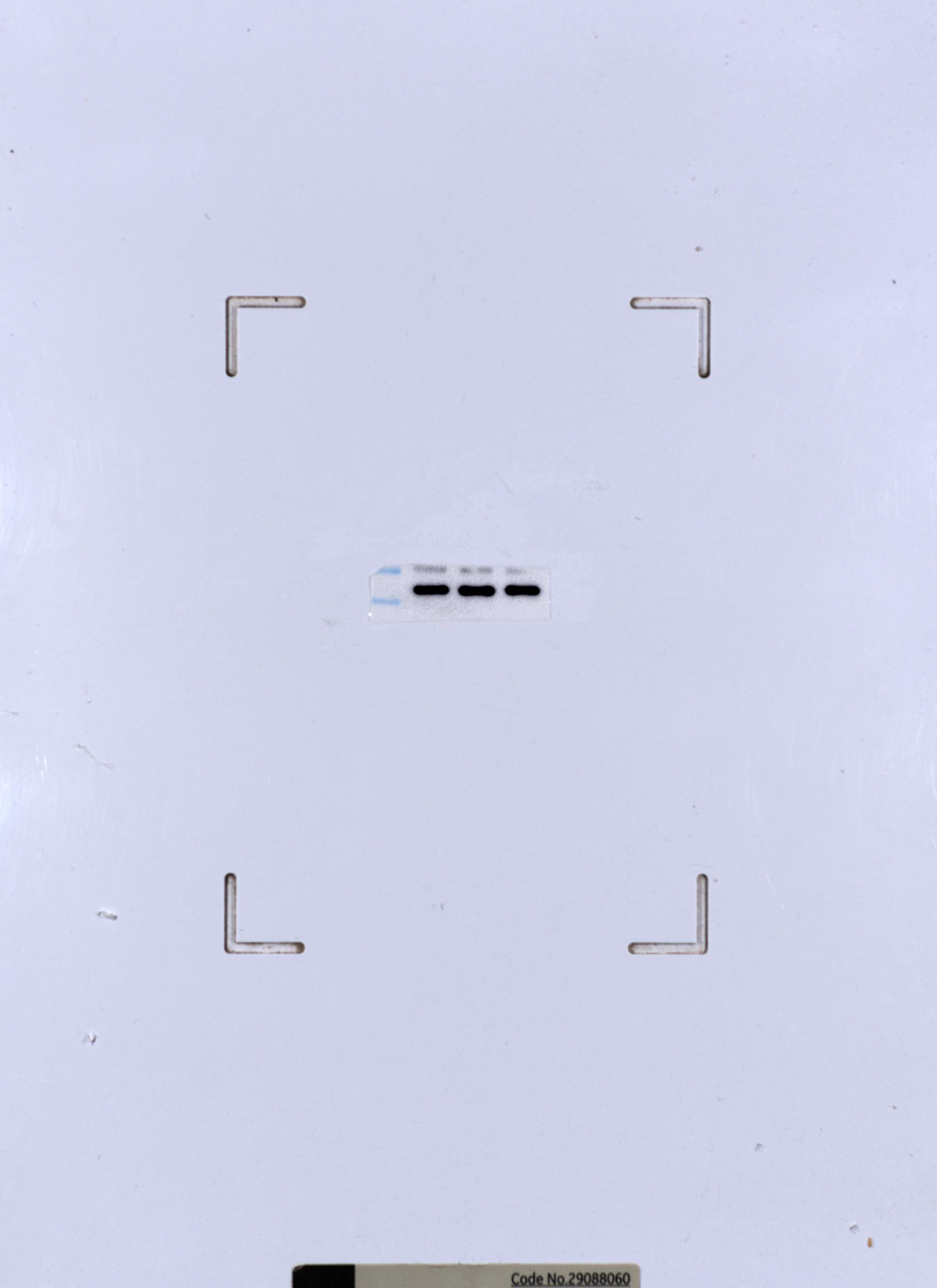

Supplement: Supplemental Information 4 [file peerj-10-12797-s004.zip › Figure 4/a┬-actin/a┬-actin2.jpg]

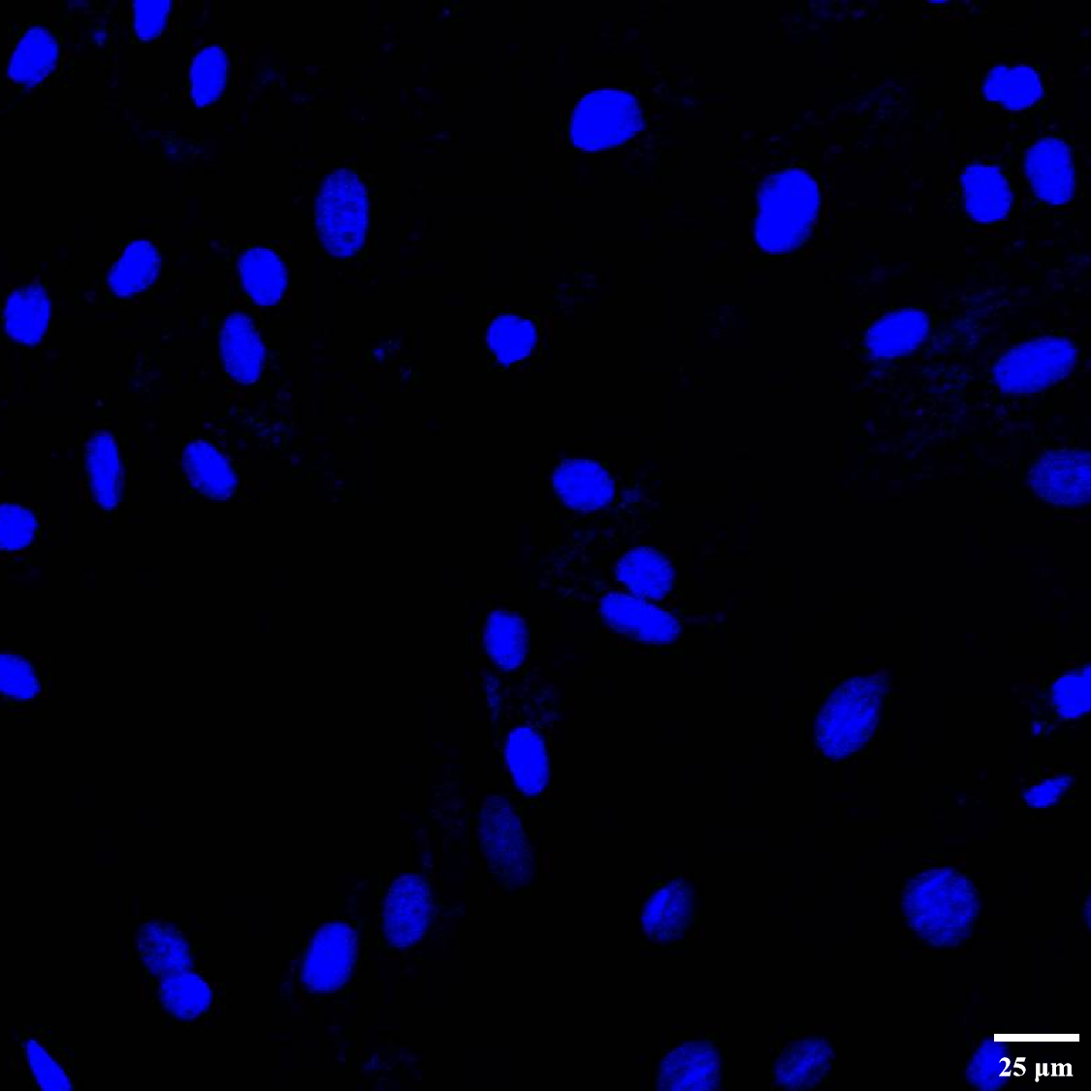

Supplement: Supplemental Information 5 [file peerj-10-12797-s005.zip › Fig-5/H9C2 FN/CON1-1.tif]

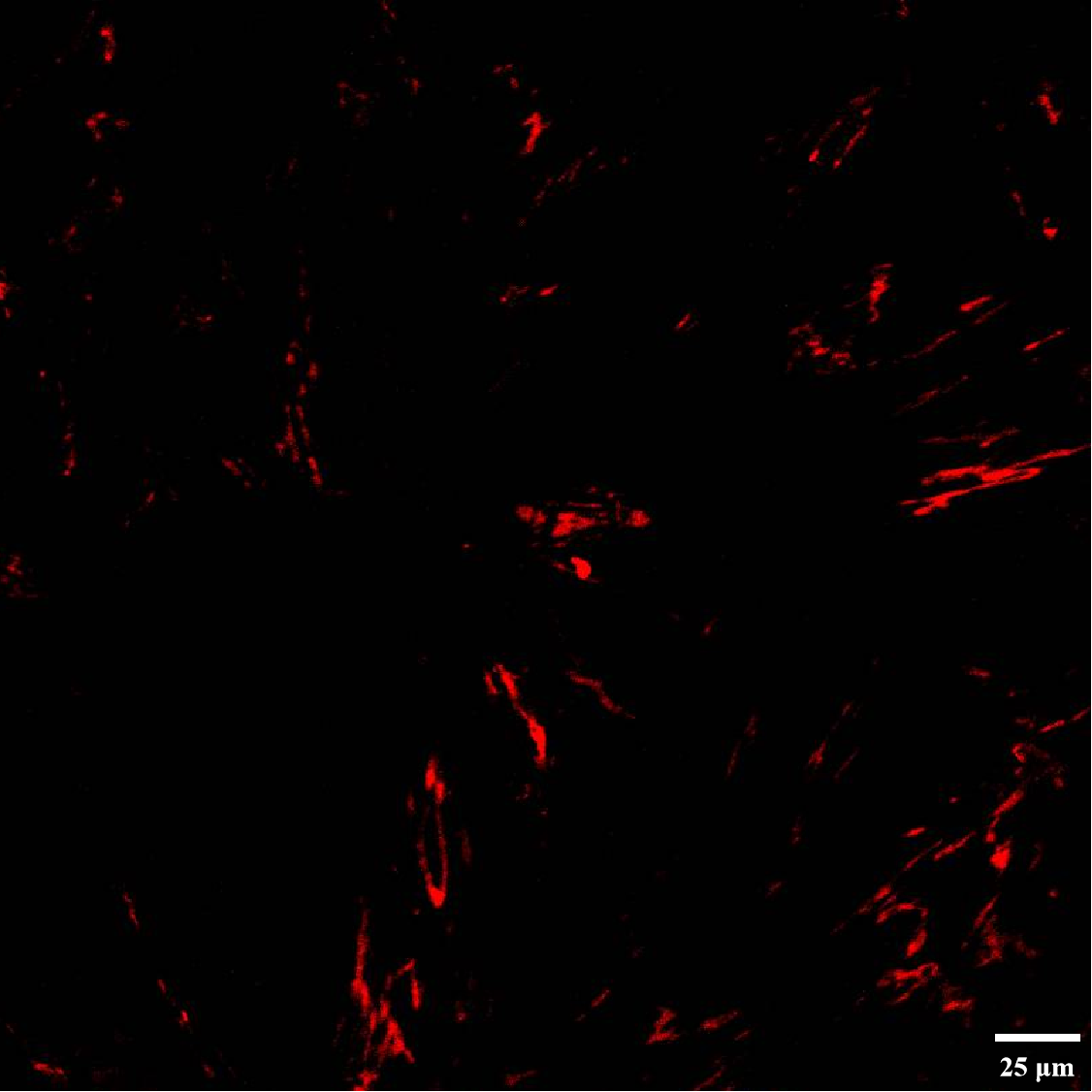

Supplement: Supplemental Information 5 [file peerj-10-12797-s005.zip › Fig-5/H9C2 FN/CON1-2.tif]

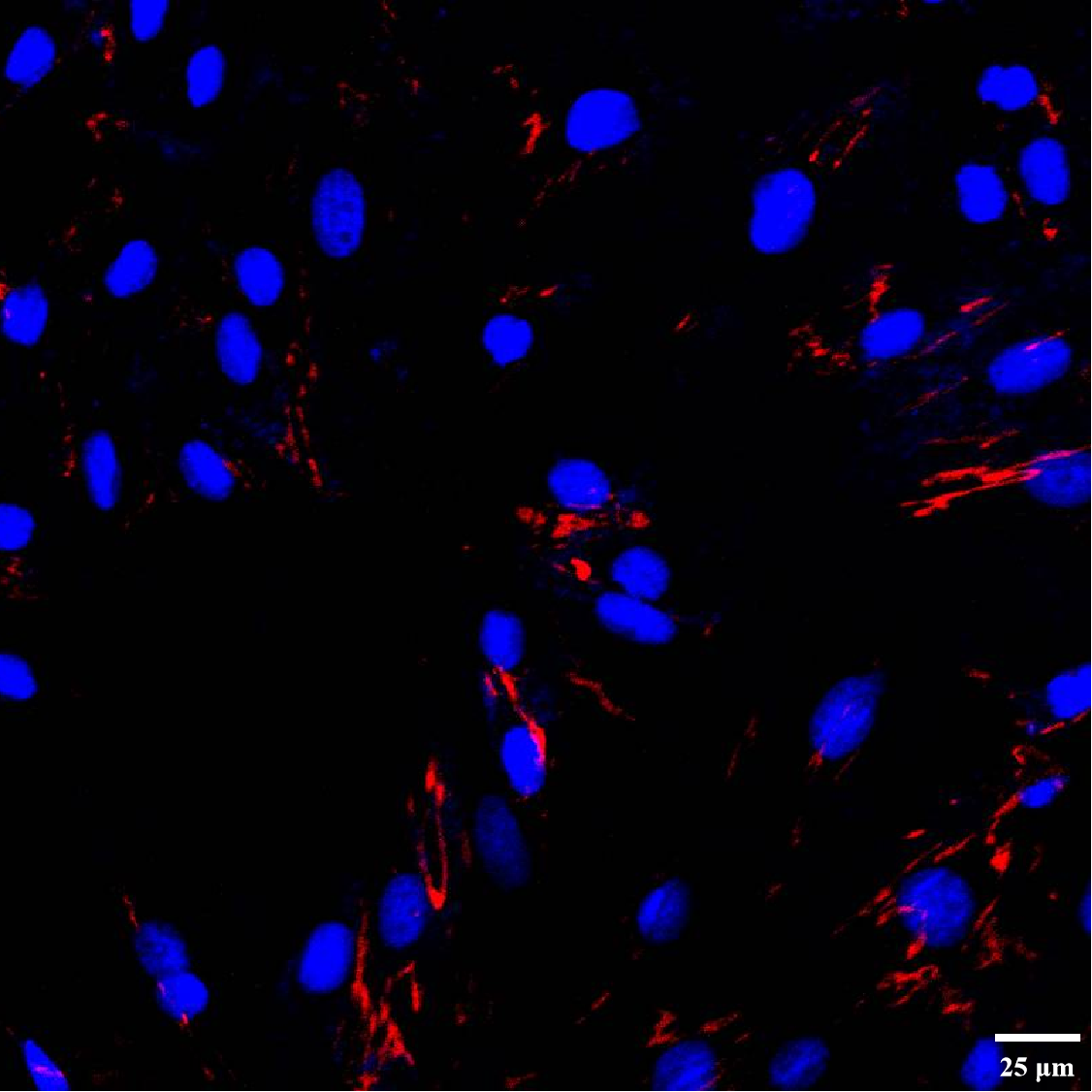

Supplement: Supplemental Information 5 [file peerj-10-12797-s005.zip › Fig-5/H9C2 FN/CON1.tif]

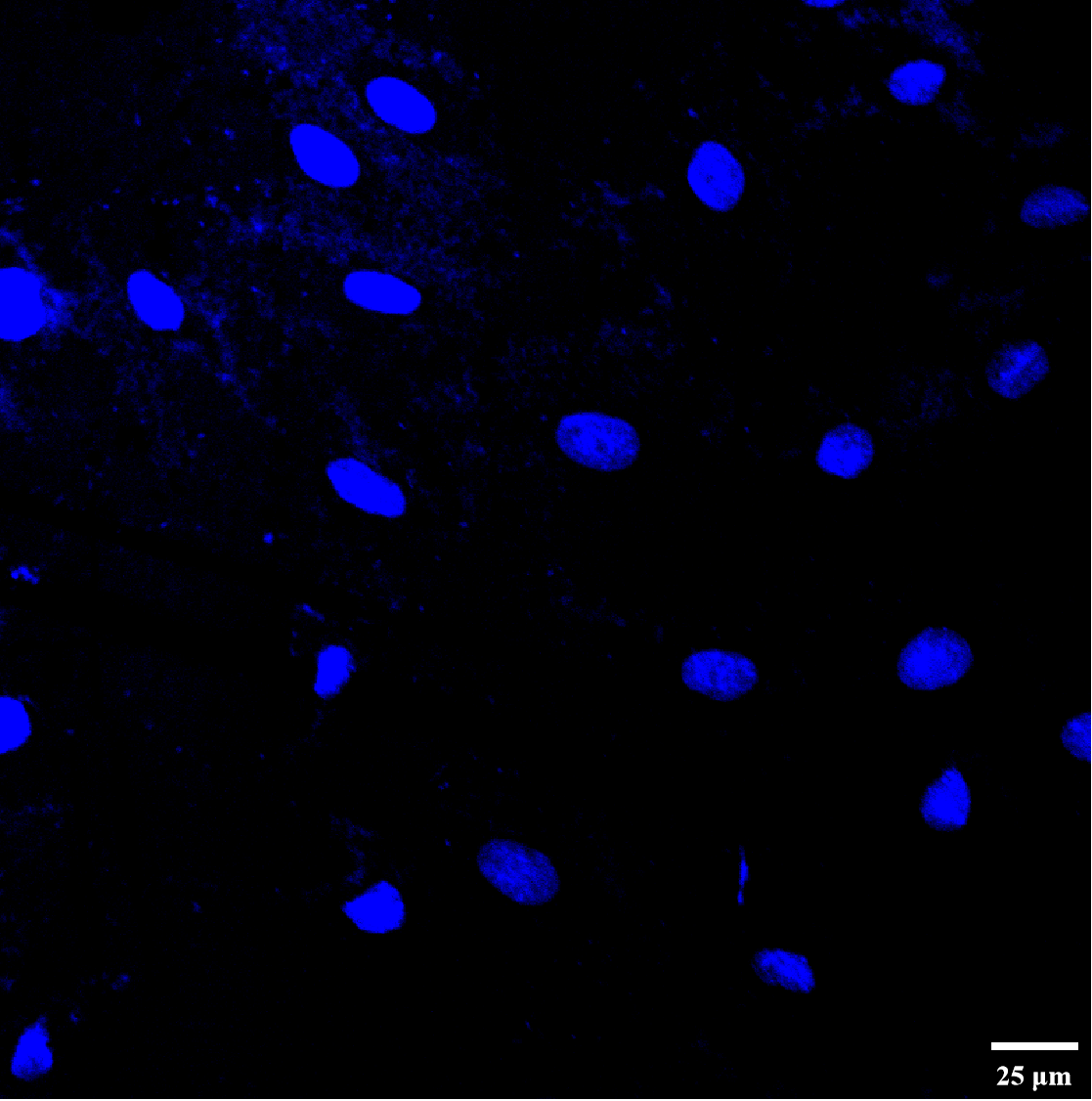

Supplement: Supplemental Information 5 [file peerj-10-12797-s005.zip › Fig-5/H9C2 FN/LAP1-1.tif]

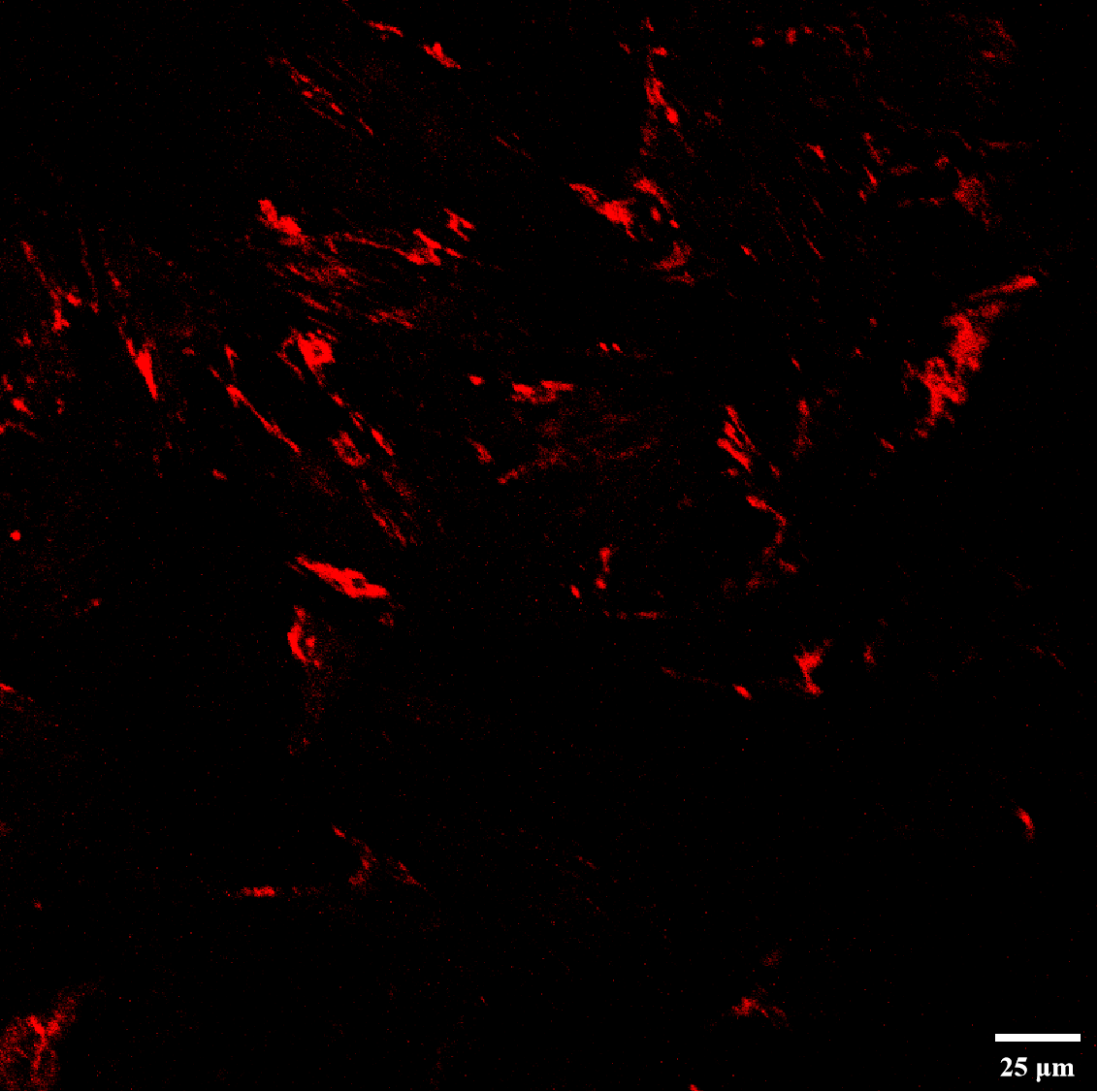

Supplement: Supplemental Information 5 [file peerj-10-12797-s005.zip › Fig-5/H9C2 FN/LAP1-2.tif]

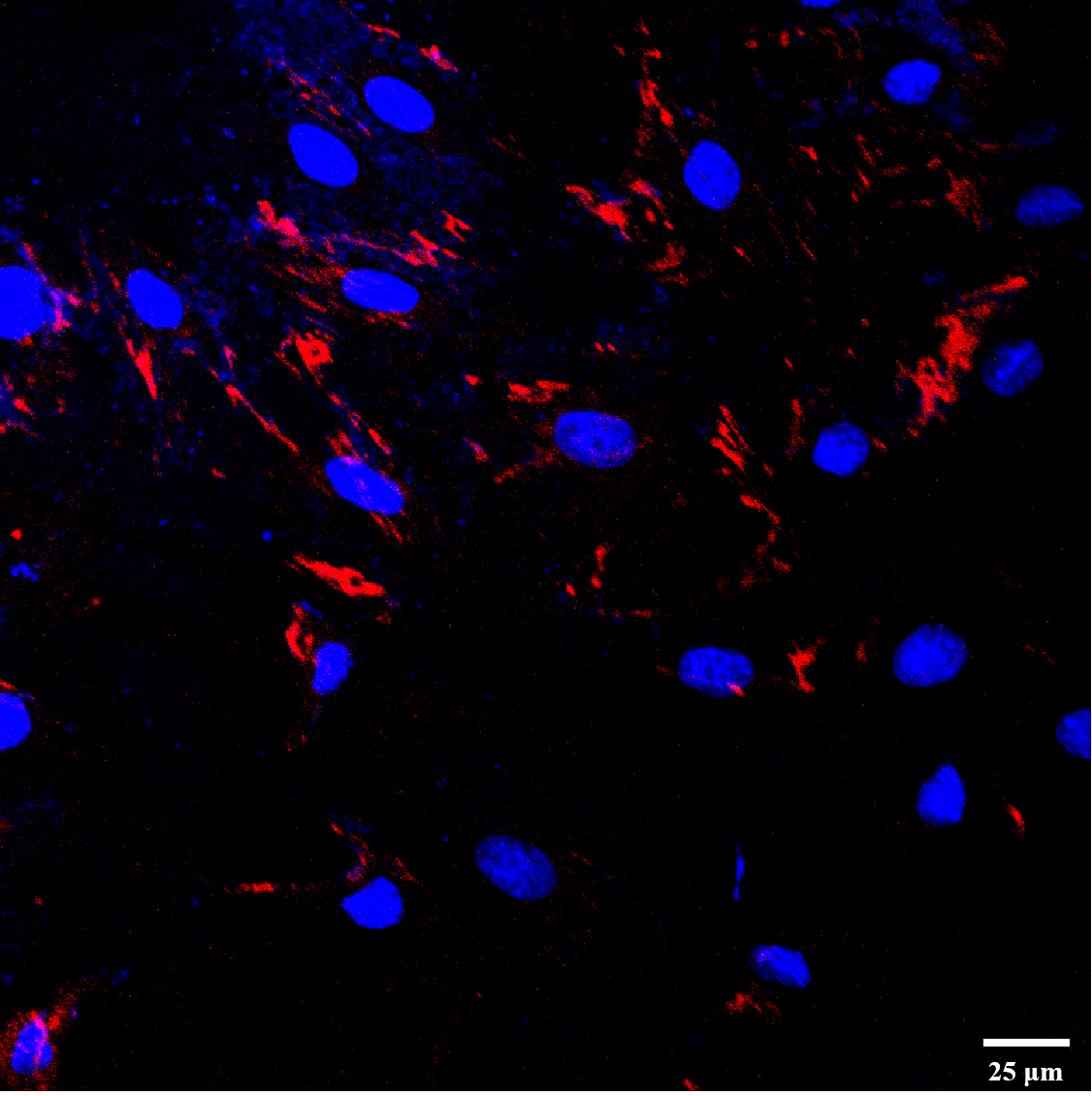

Supplement: Supplemental Information 5 [file peerj-10-12797-s005.zip › Fig-5/H9C2 FN/LAP1.tif]

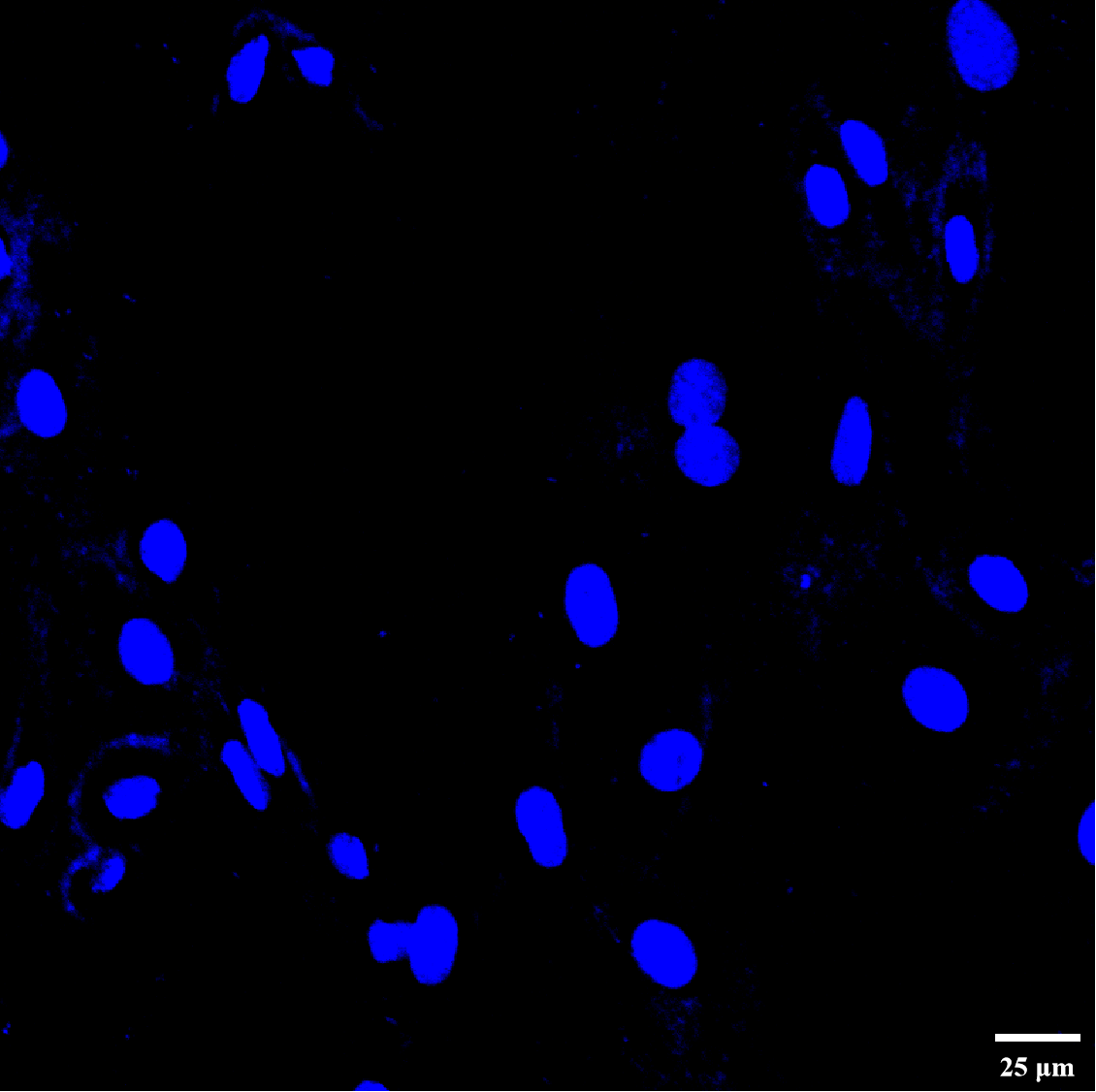

Supplement: Supplemental Information 5 [file peerj-10-12797-s005.zip › Fig-5/H9C2 FN/TGF1-1.tif]

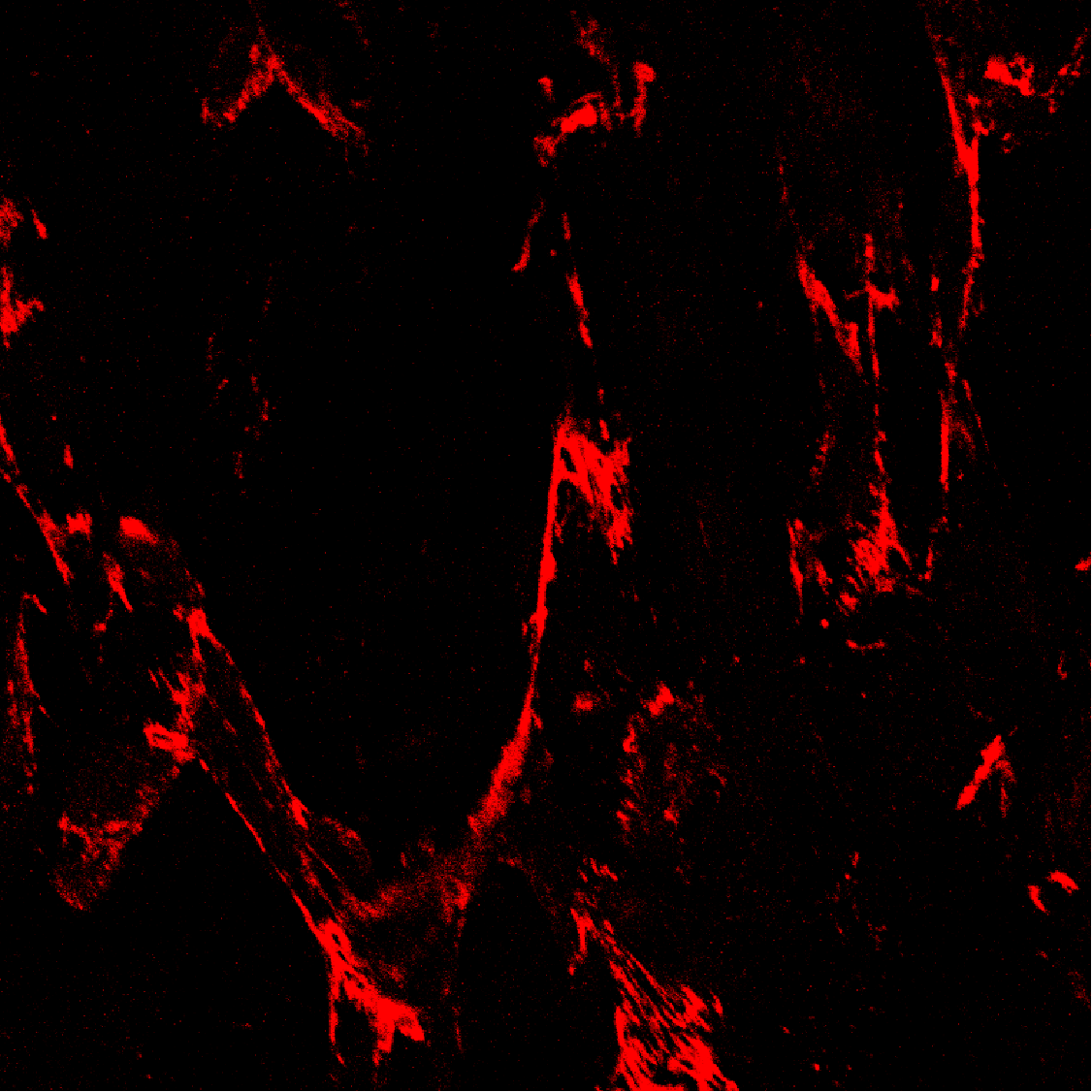

Supplement: Supplemental Information 5 [file peerj-10-12797-s005.zip › Fig-5/H9C2 FN/TGF1-2.tif]

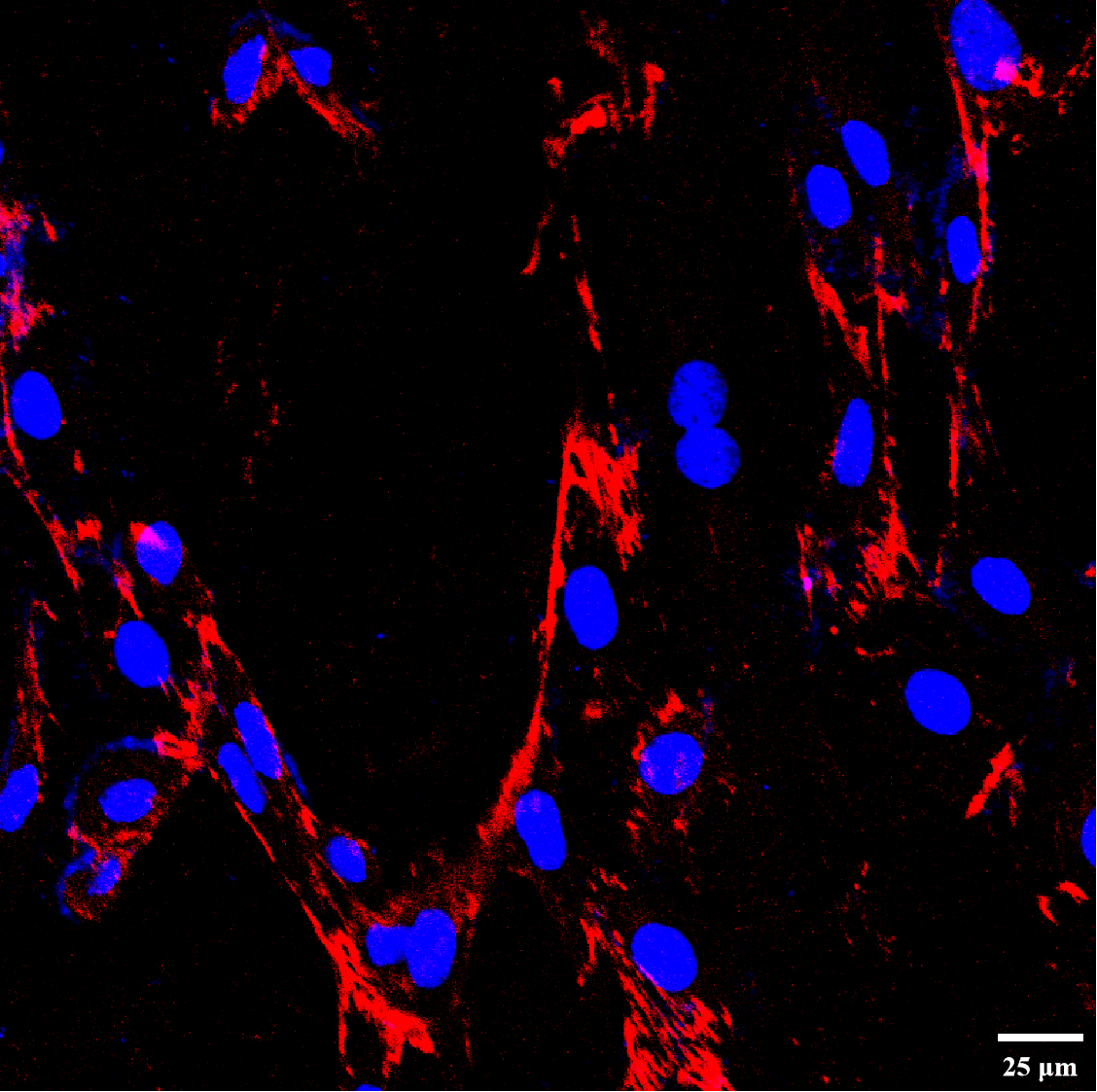

Supplement: Supplemental Information 5 [file peerj-10-12797-s005.zip › Fig-5/H9C2 FN/TGF1.tif]

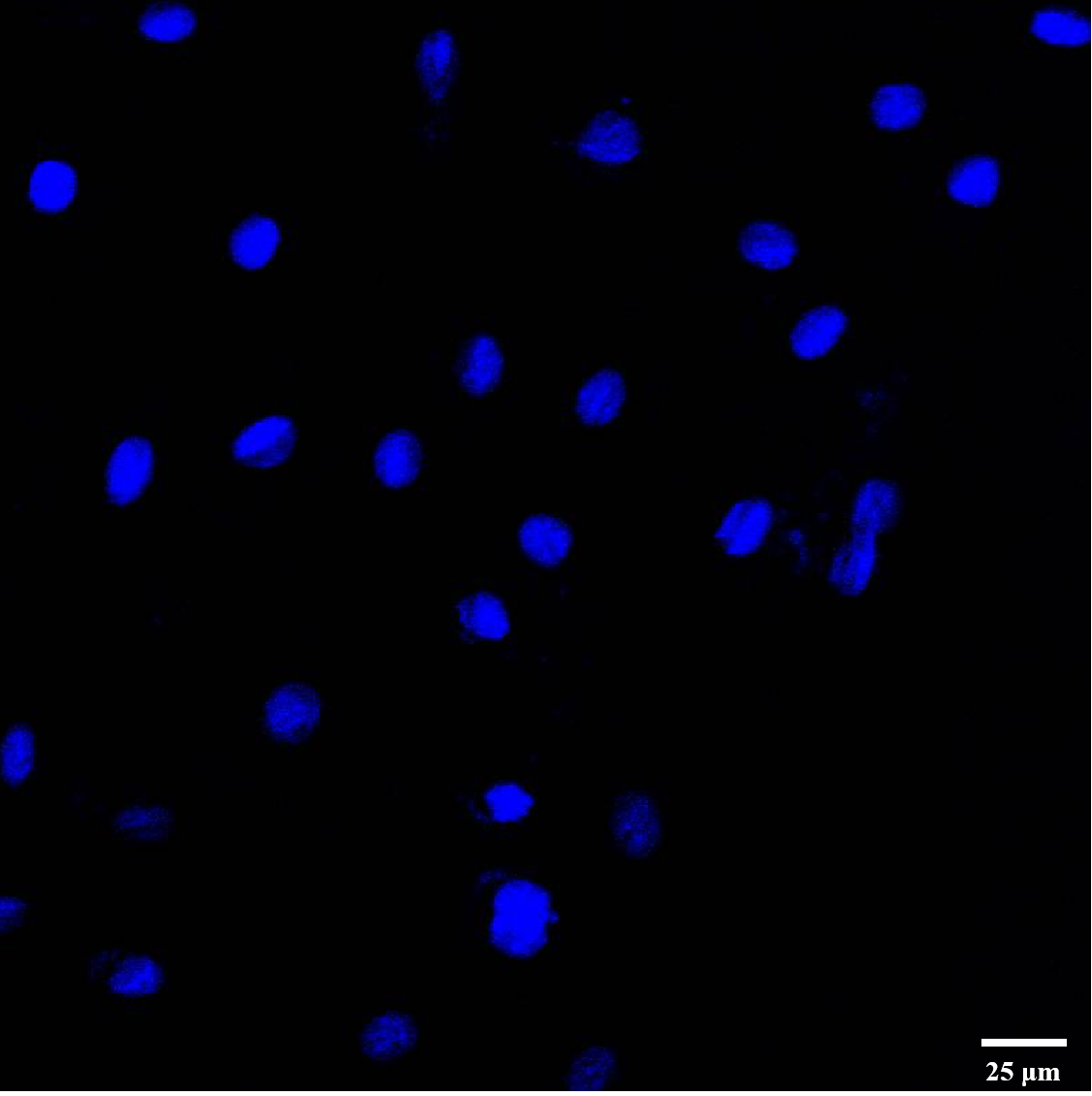

Supplement: Supplemental Information 5 [file peerj-10-12797-s005.zip › Fig-5/H9C2 SMA/CON1-1.tif]

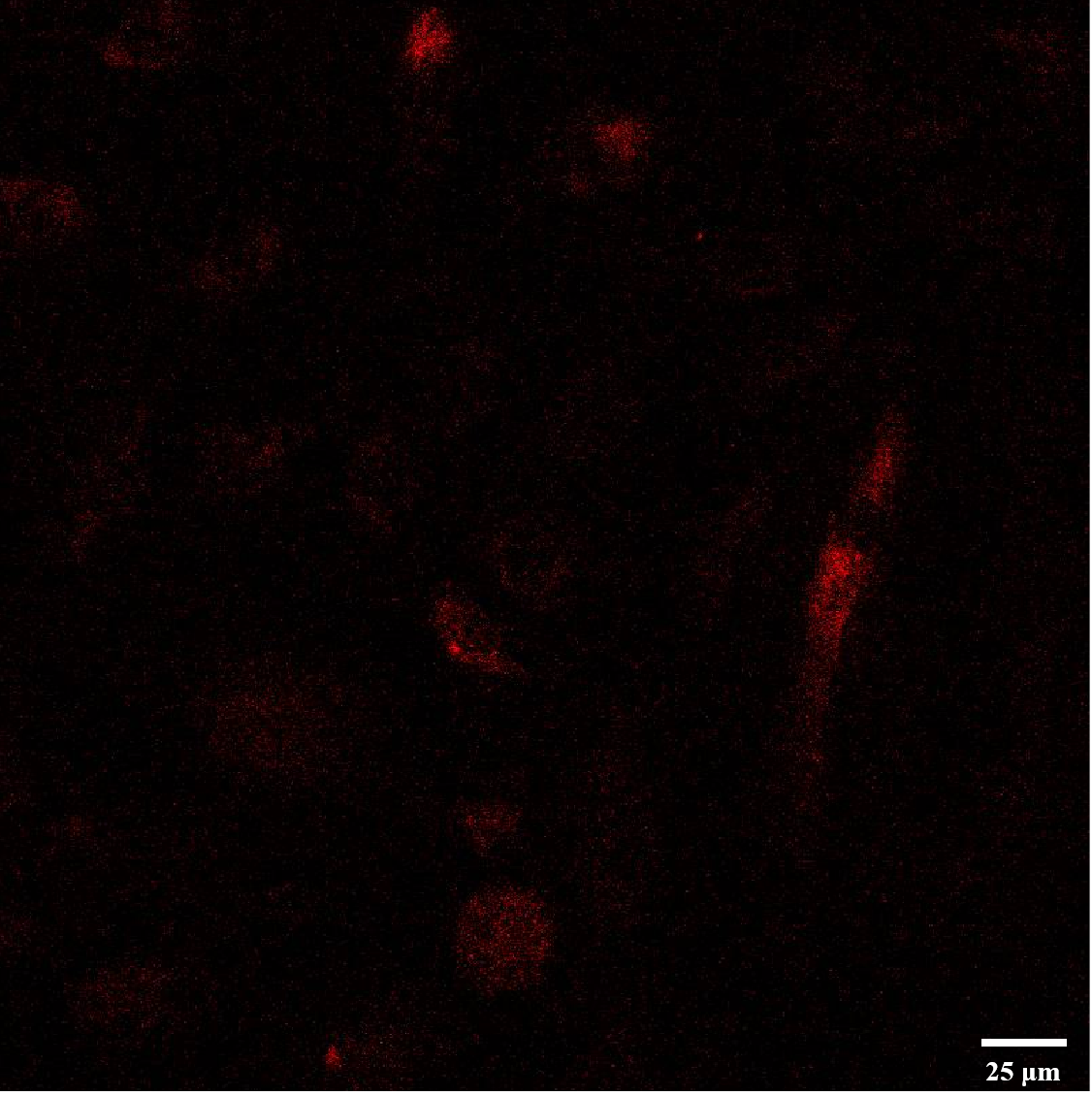

Supplement: Supplemental Information 5 [file peerj-10-12797-s005.zip › Fig-5/H9C2 SMA/CON1-2.tif]

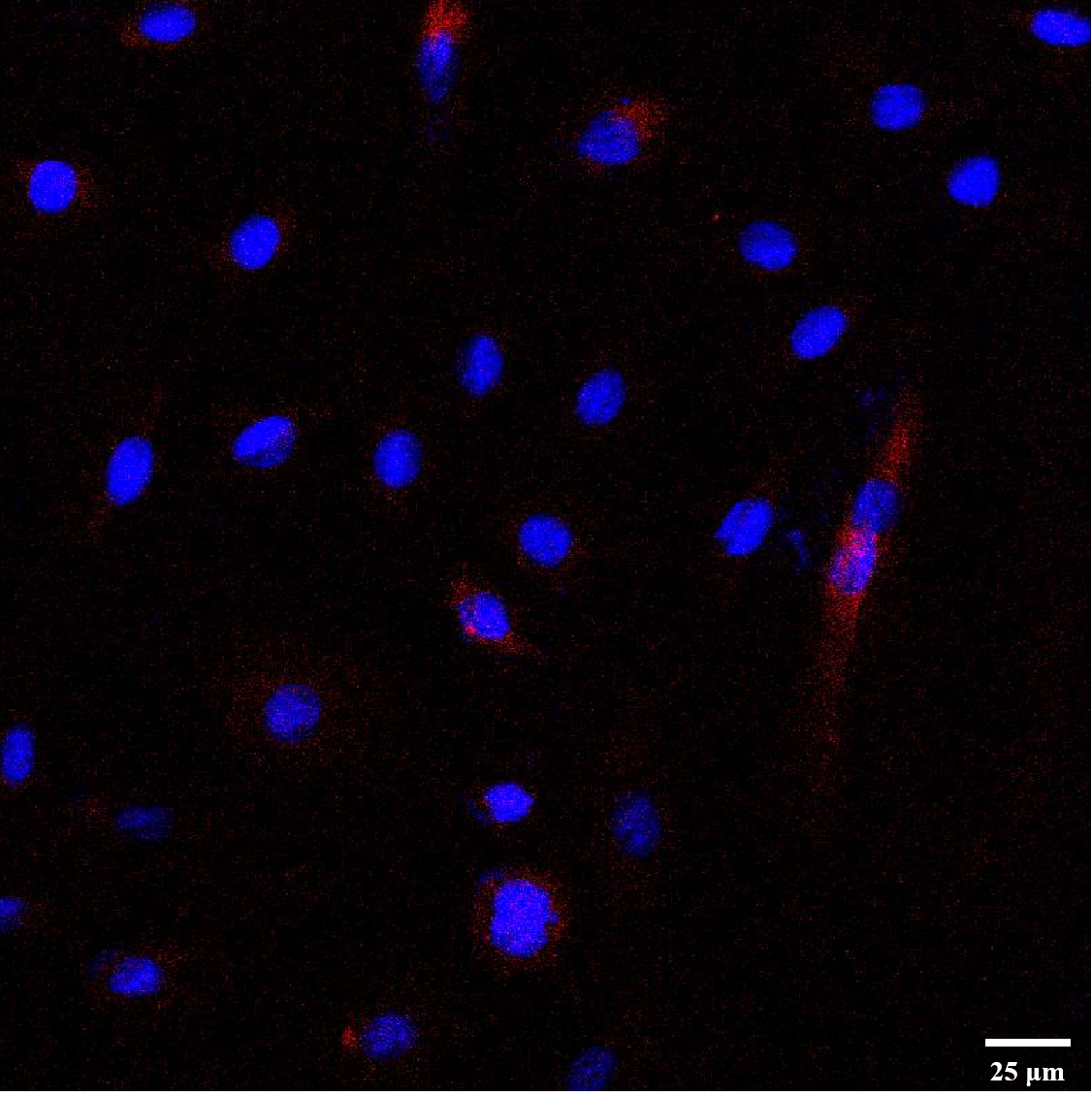

Supplement: Supplemental Information 5 [file peerj-10-12797-s005.zip › Fig-5/H9C2 SMA/CON1.tif]

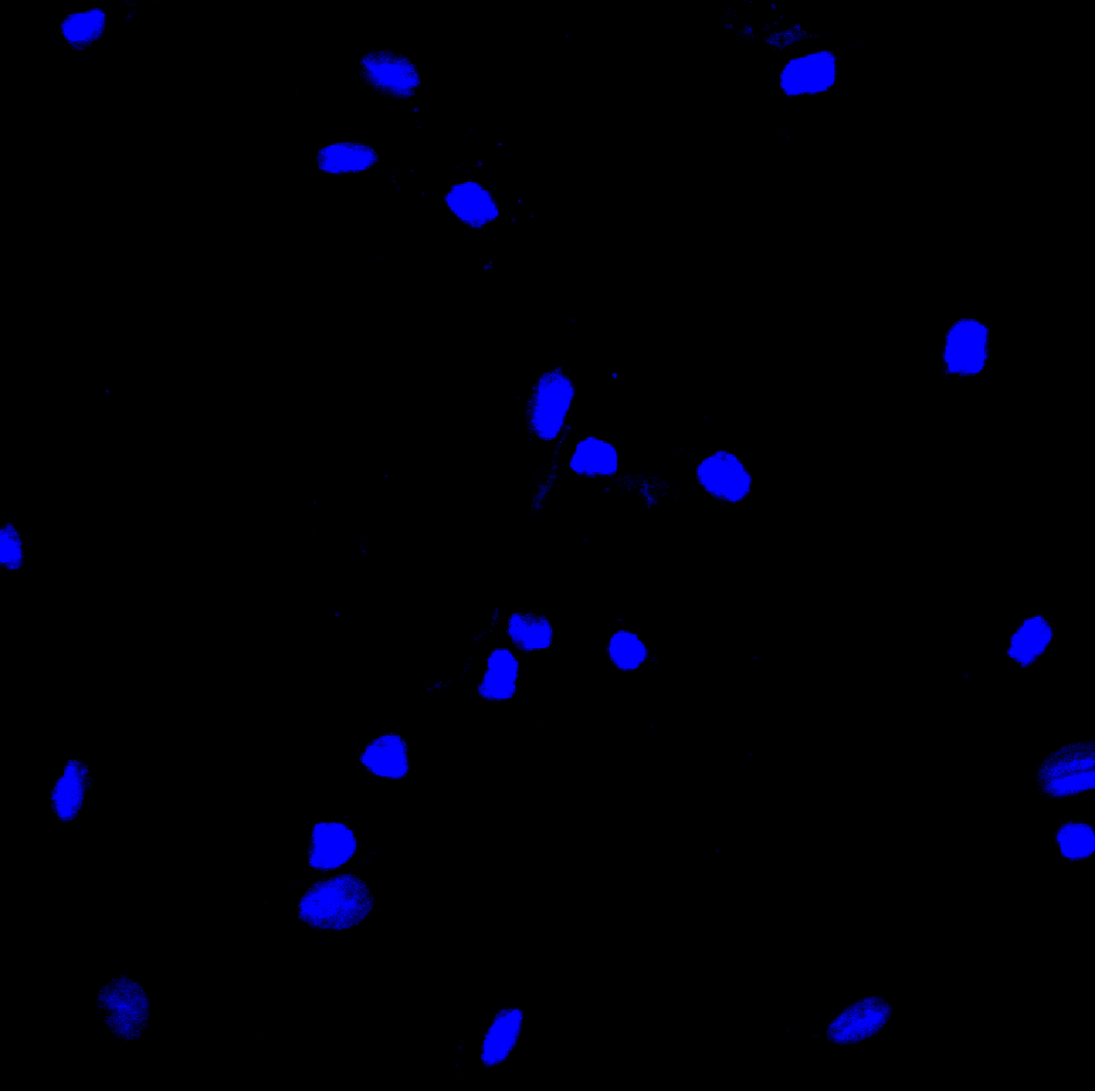

Supplement: Supplemental Information 5 [file peerj-10-12797-s005.zip › Fig-5/H9C2 SMA/LAP1-1.tif]

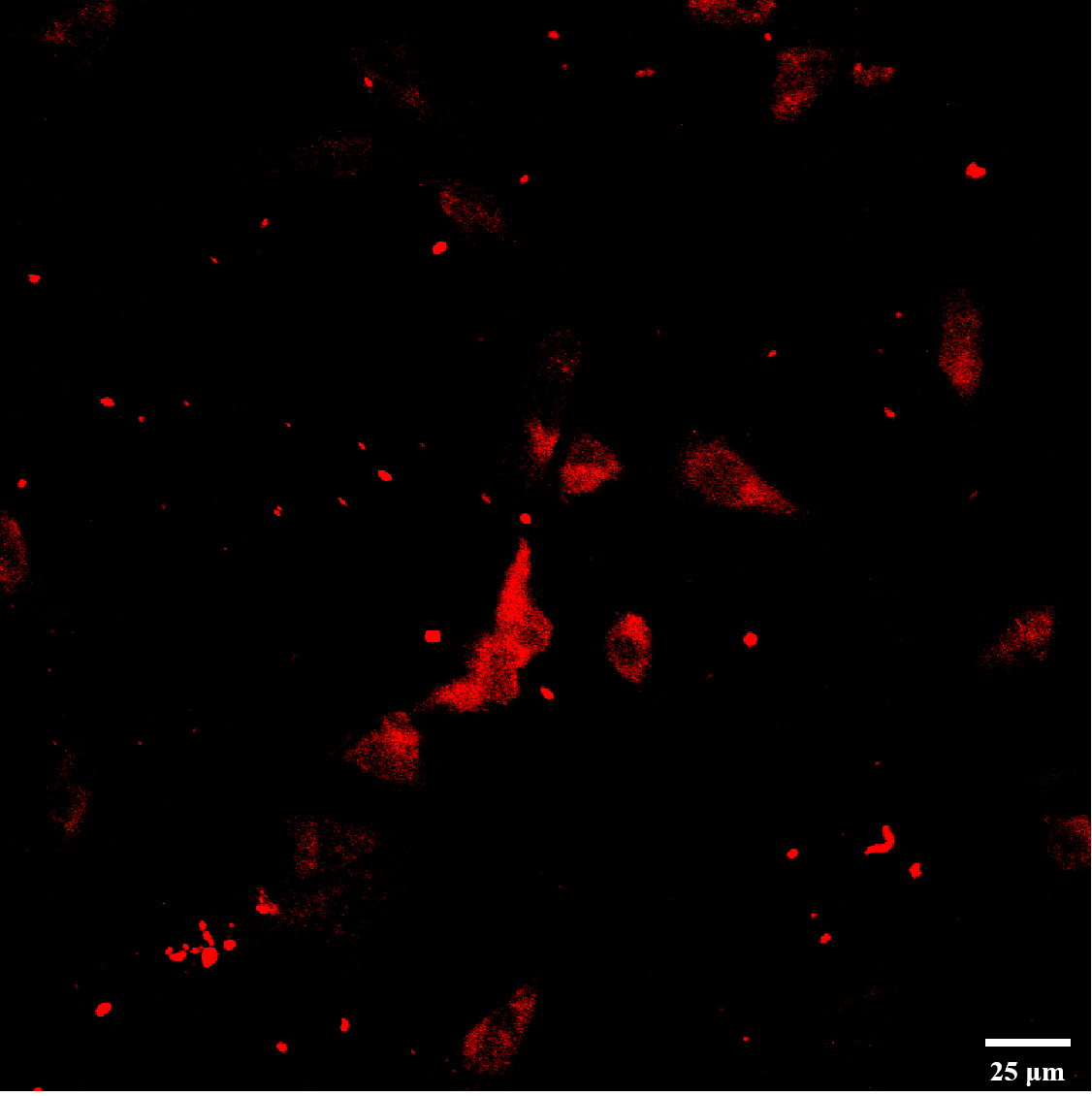

Supplement: Supplemental Information 5 [file peerj-10-12797-s005.zip › Fig-5/H9C2 SMA/LAP1-2.tif]

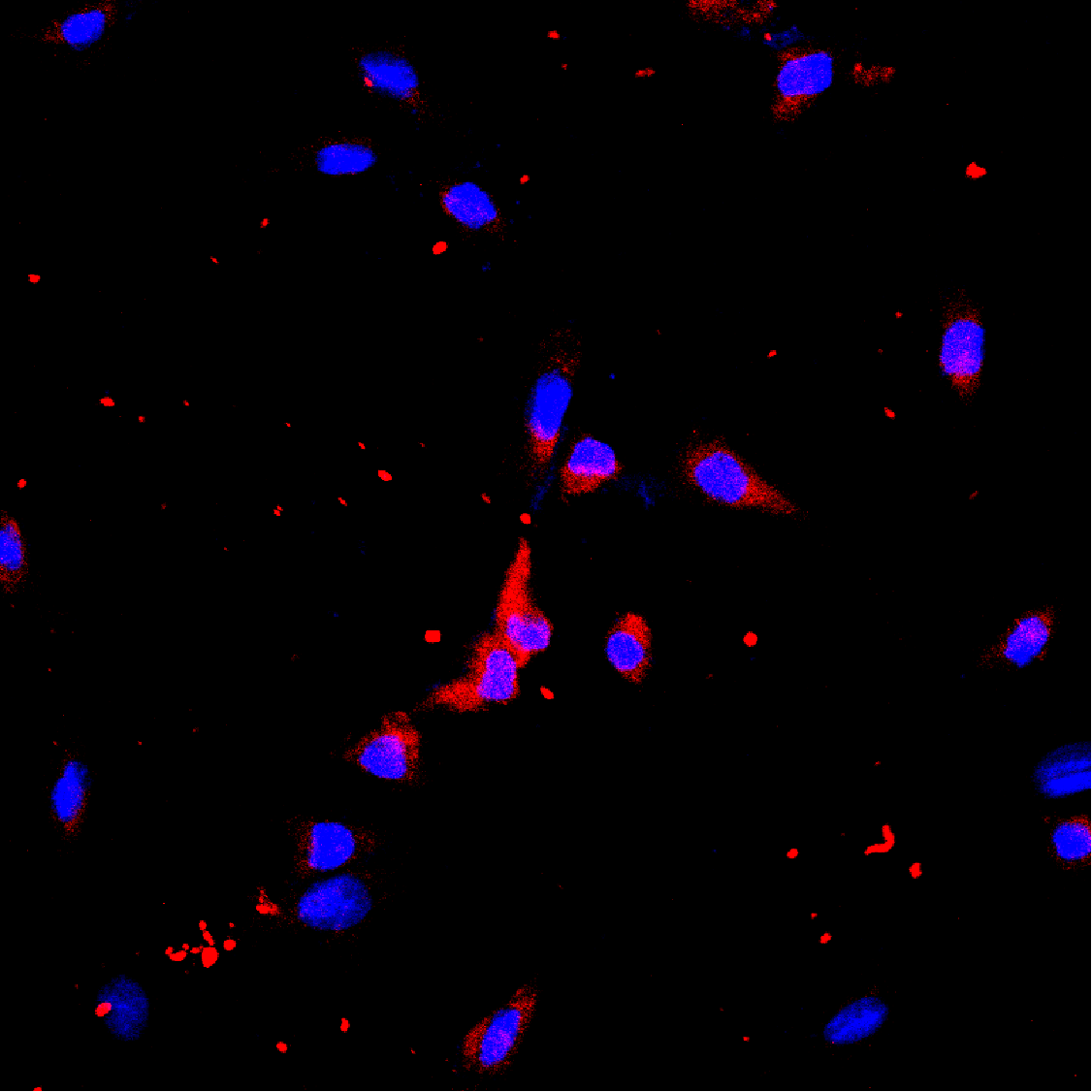

Supplement: Supplemental Information 5 [file peerj-10-12797-s005.zip › Fig-5/H9C2 SMA/LAP1.tif]

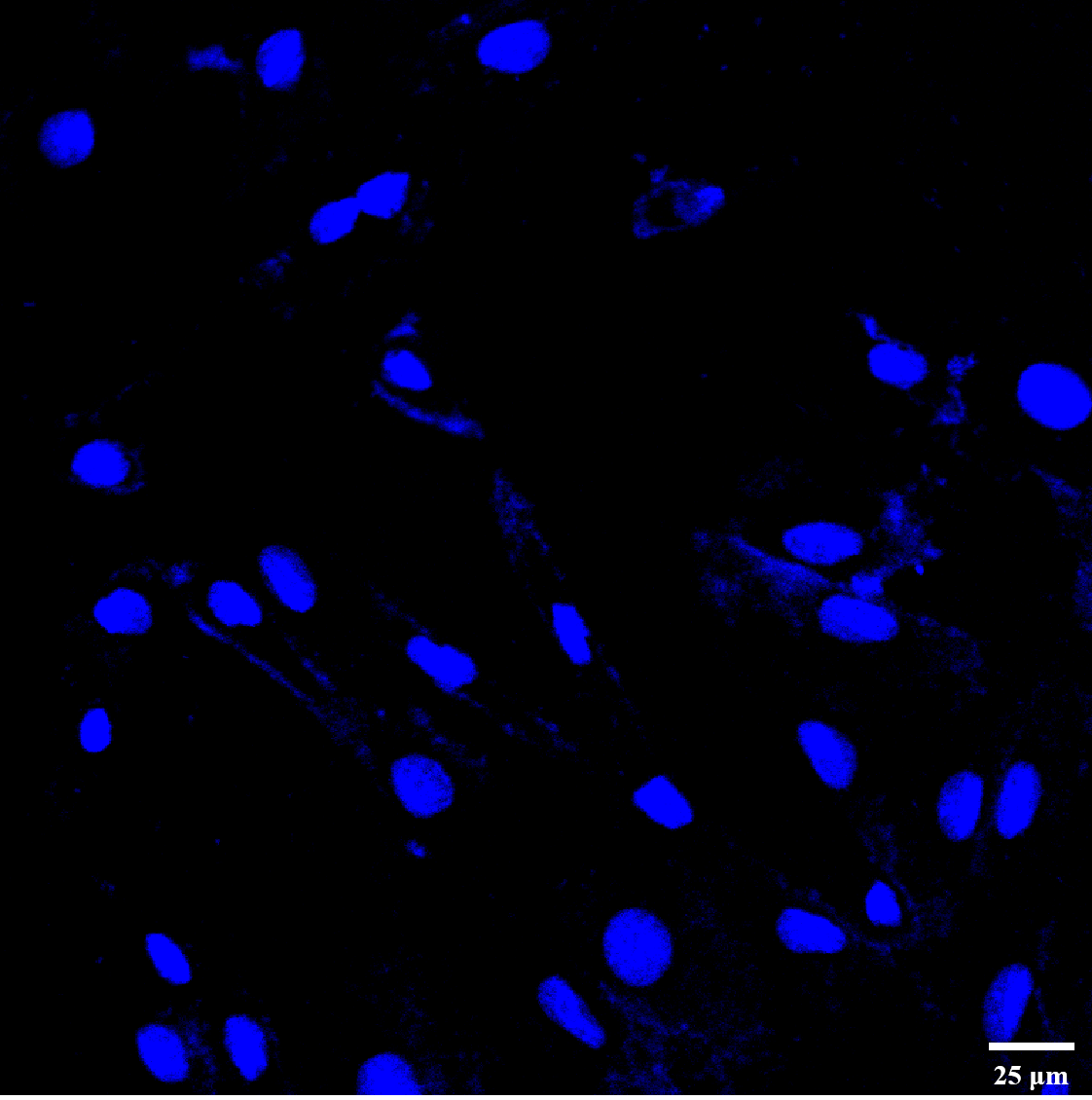

Supplement: Supplemental Information 5 [file peerj-10-12797-s005.zip › Fig-5/H9C2 SMA/TGF1-1.tif]

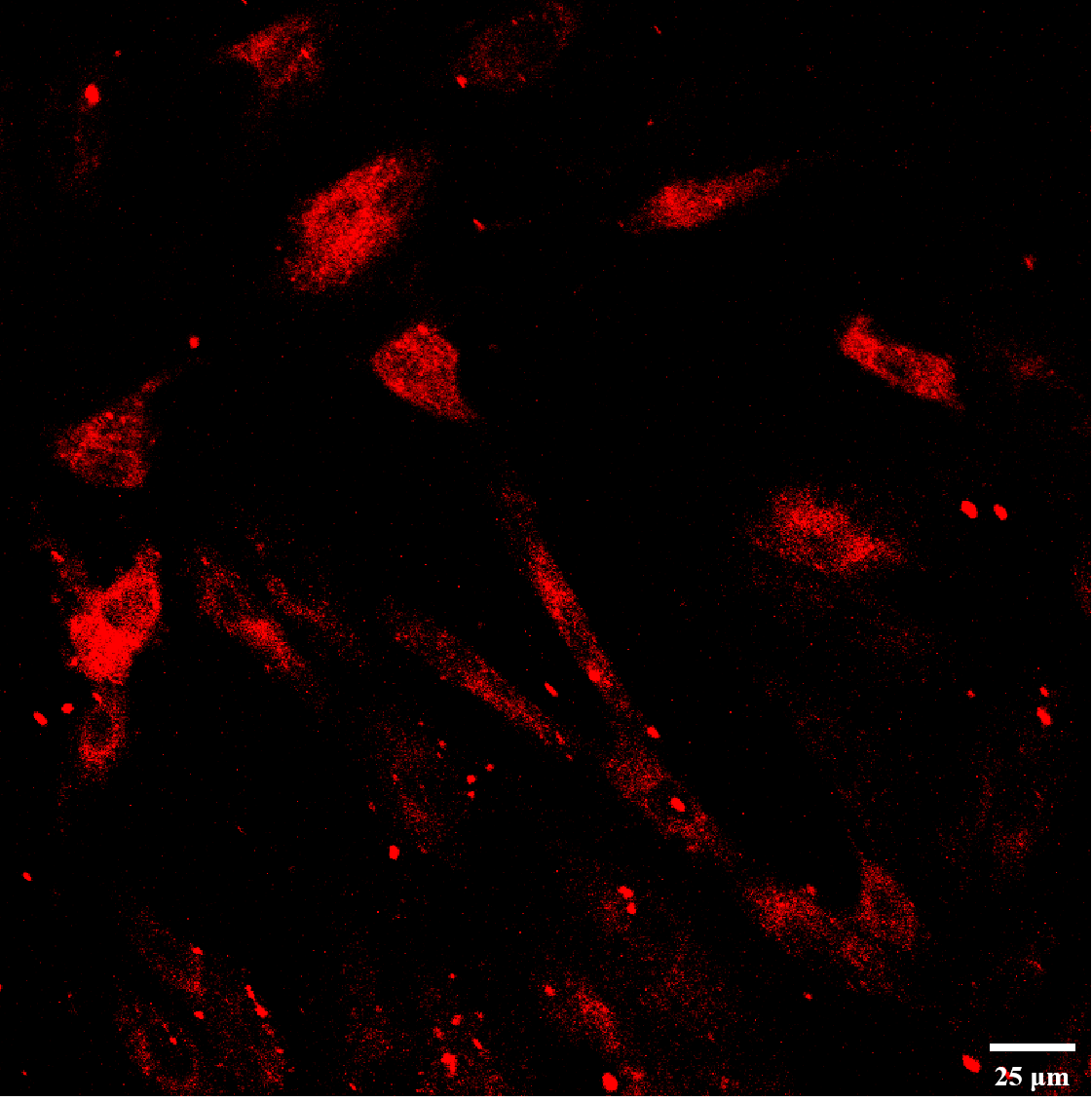

Supplement: Supplemental Information 5 [file peerj-10-12797-s005.zip › Fig-5/H9C2 SMA/TGF1-2.tif]

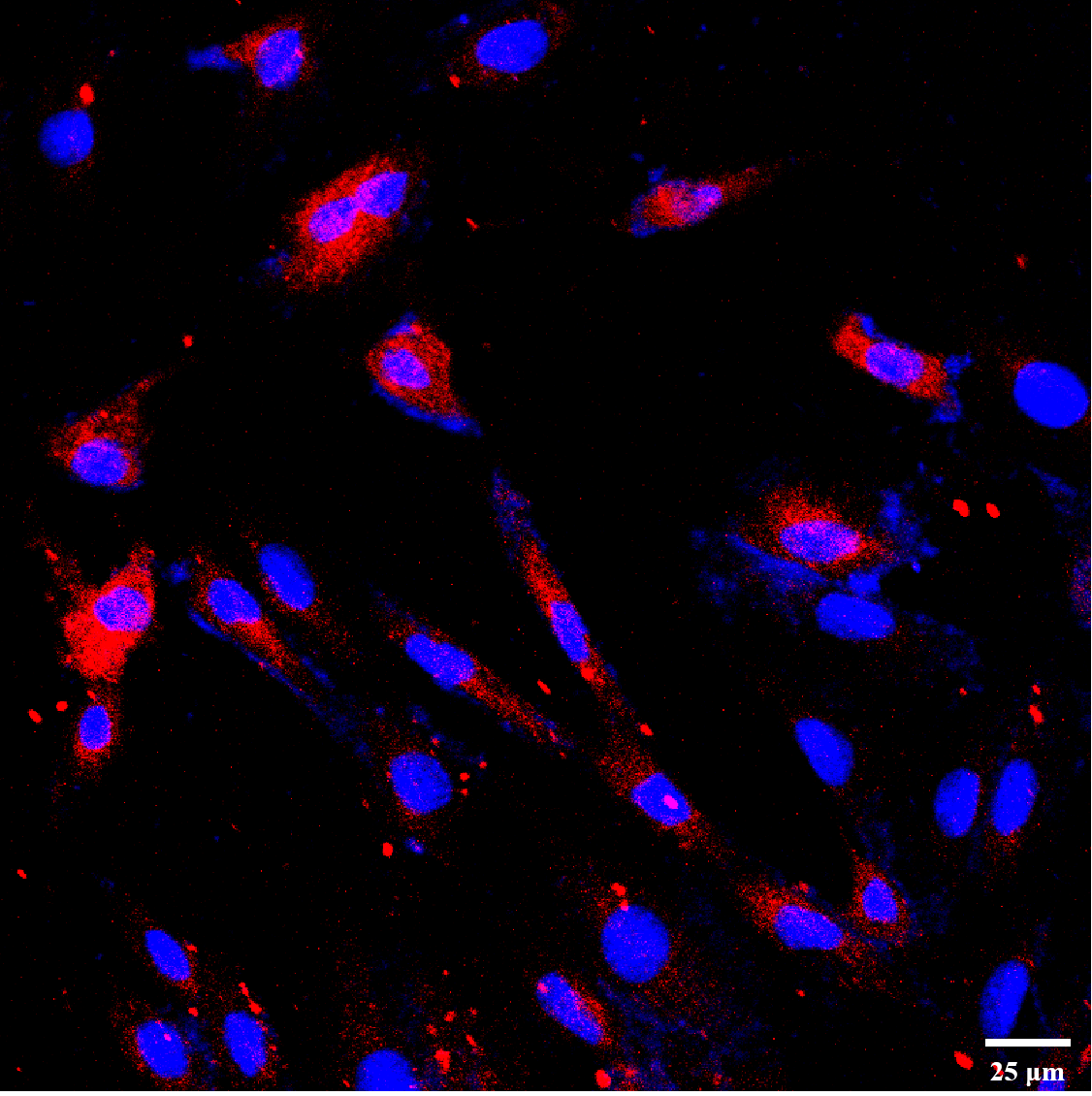

Supplement: Supplemental Information 5 [file peerj-10-12797-s005.zip › Fig-5/H9C2 SMA/TGF1.tif]

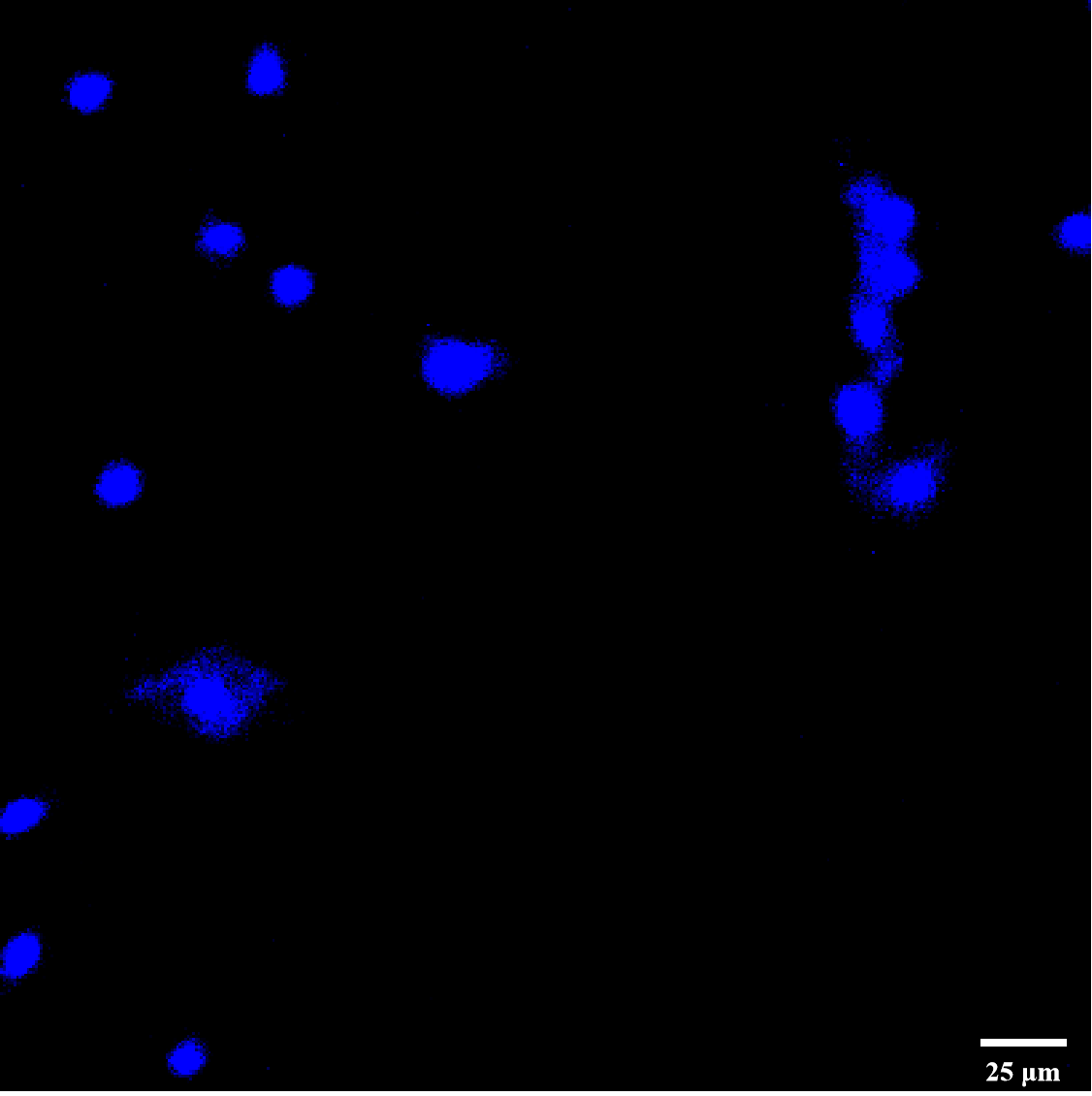

Supplement: Supplemental Information 5 [file peerj-10-12797-s005.zip › Fig-5/H9C2 col1/LAP1-1.tif]

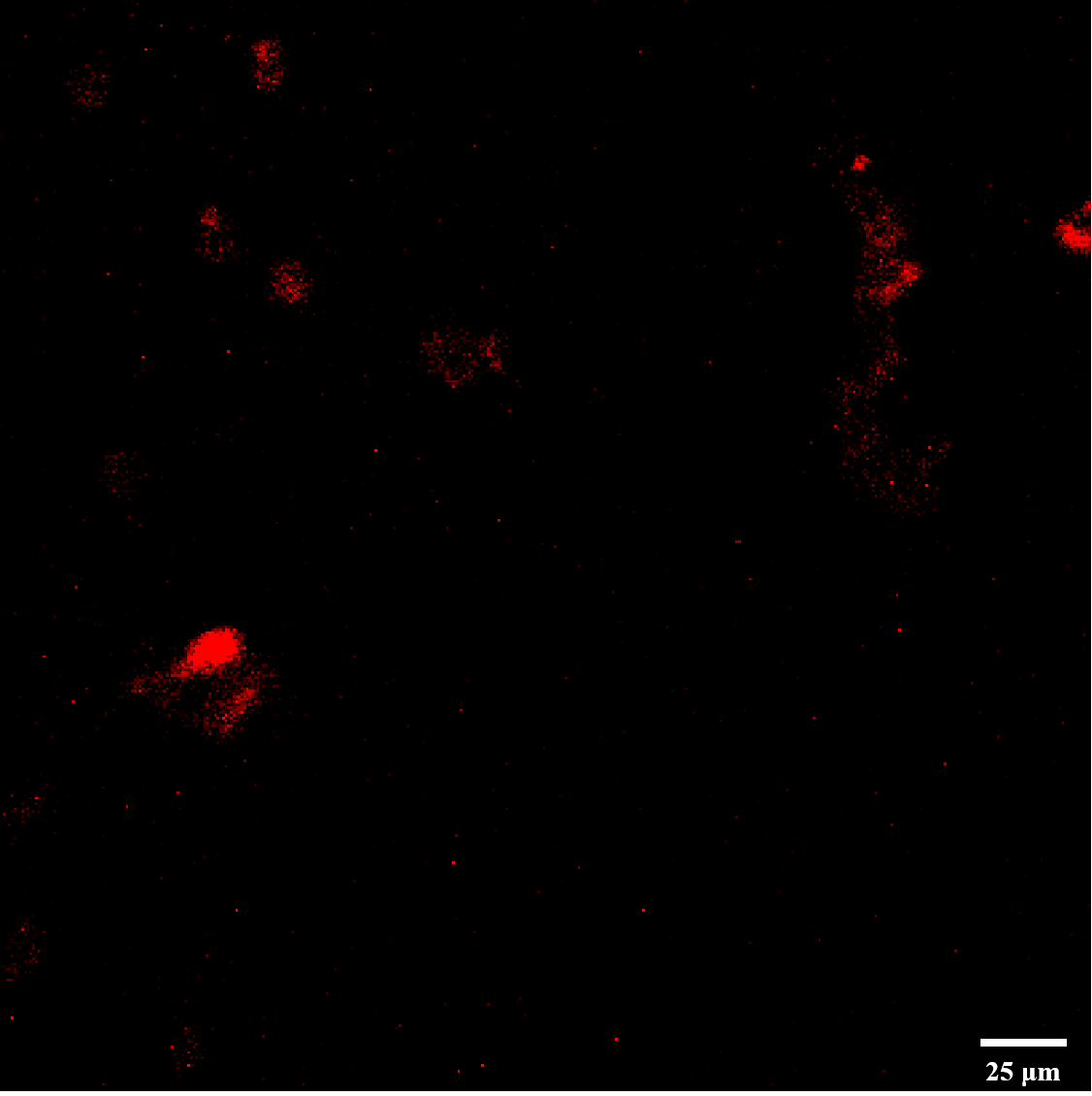

Supplement: Supplemental Information 5 [file peerj-10-12797-s005.zip › Fig-5/H9C2 col1/LAP1-2.tif]

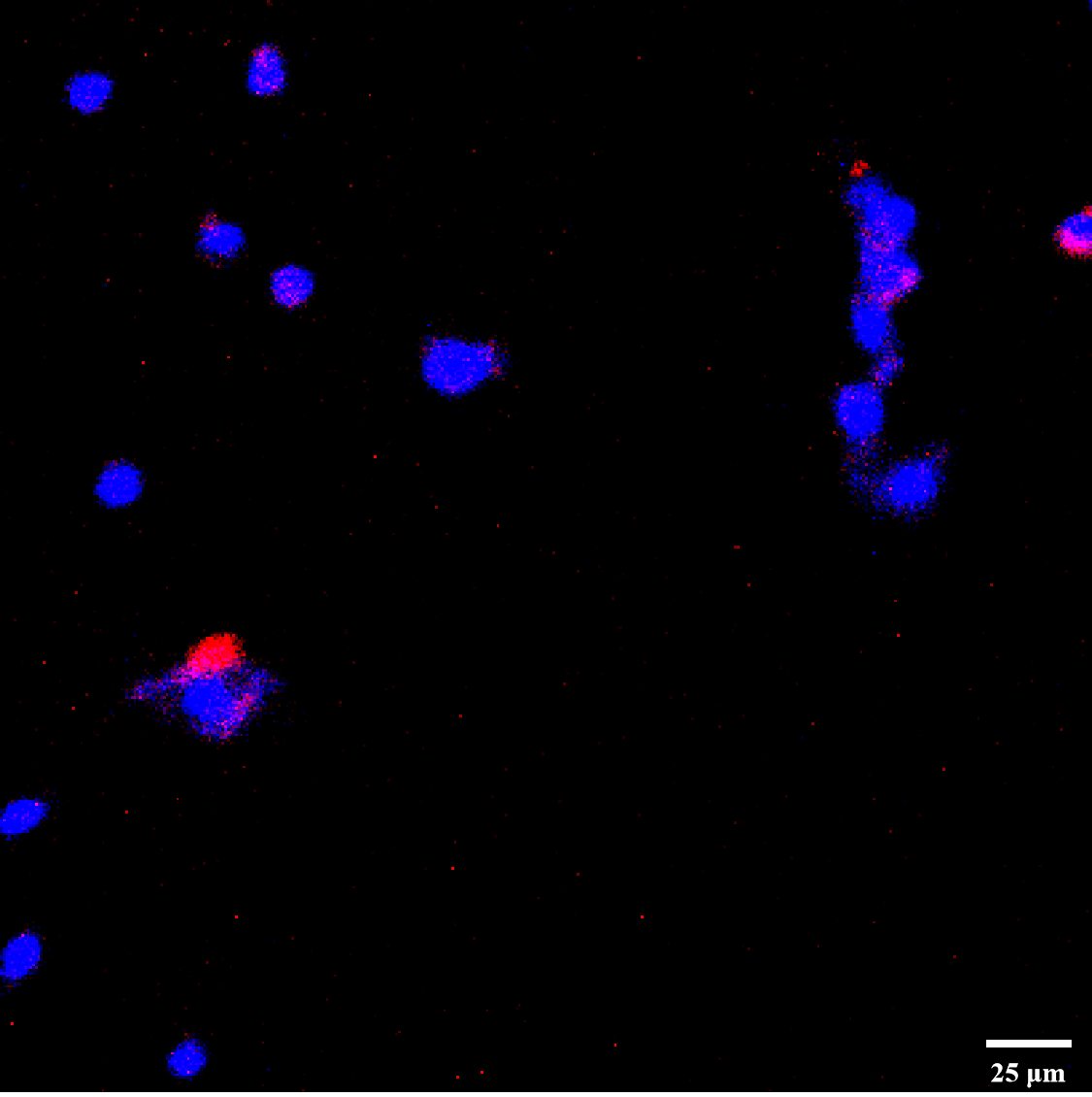

Supplement: Supplemental Information 5 [file peerj-10-12797-s005.zip › Fig-5/H9C2 col1/LAP1.tif]

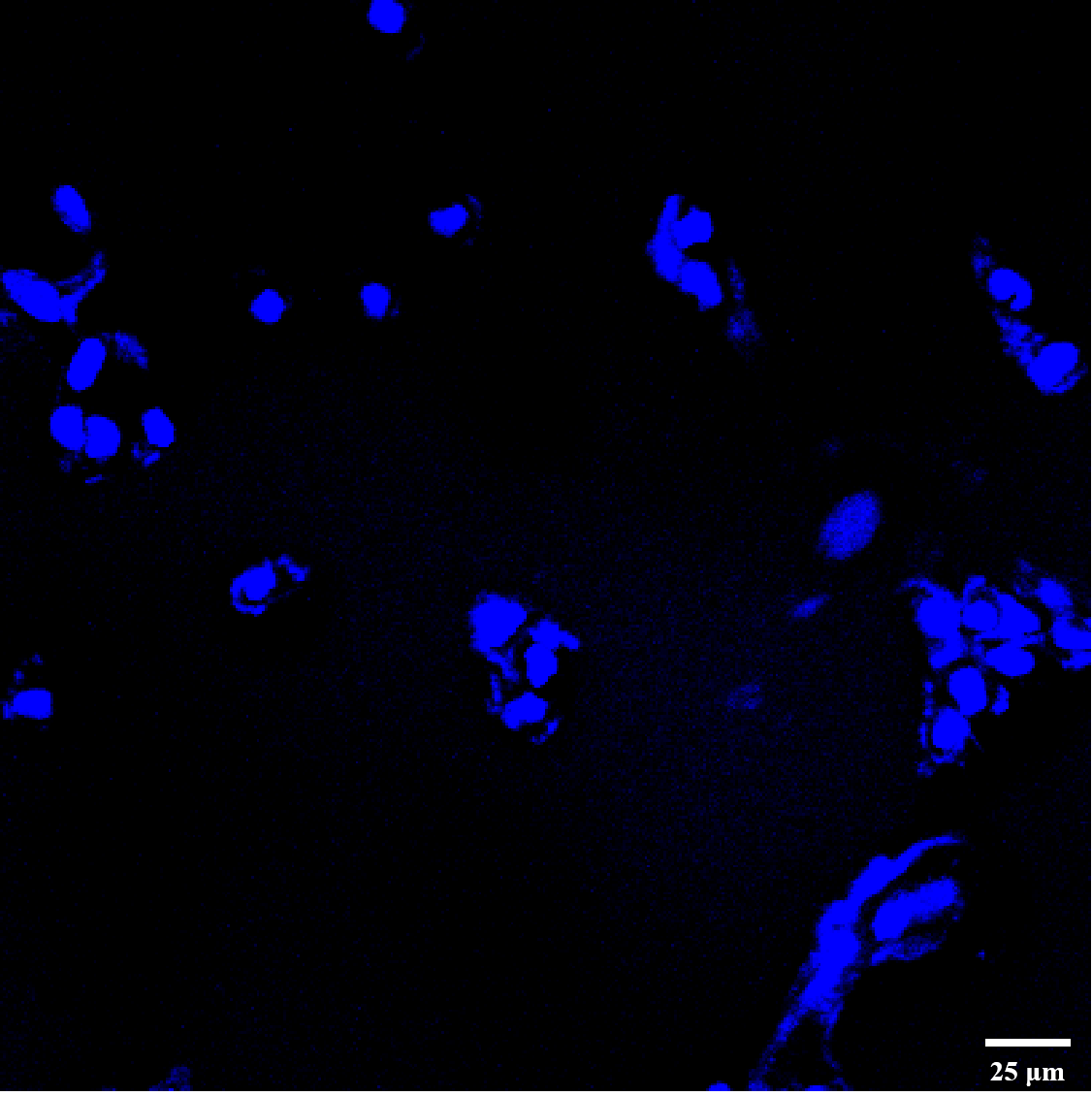

Supplement: Supplemental Information 5 [file peerj-10-12797-s005.zip › Fig-5/H9C2 col1/TGF1-1.tif]

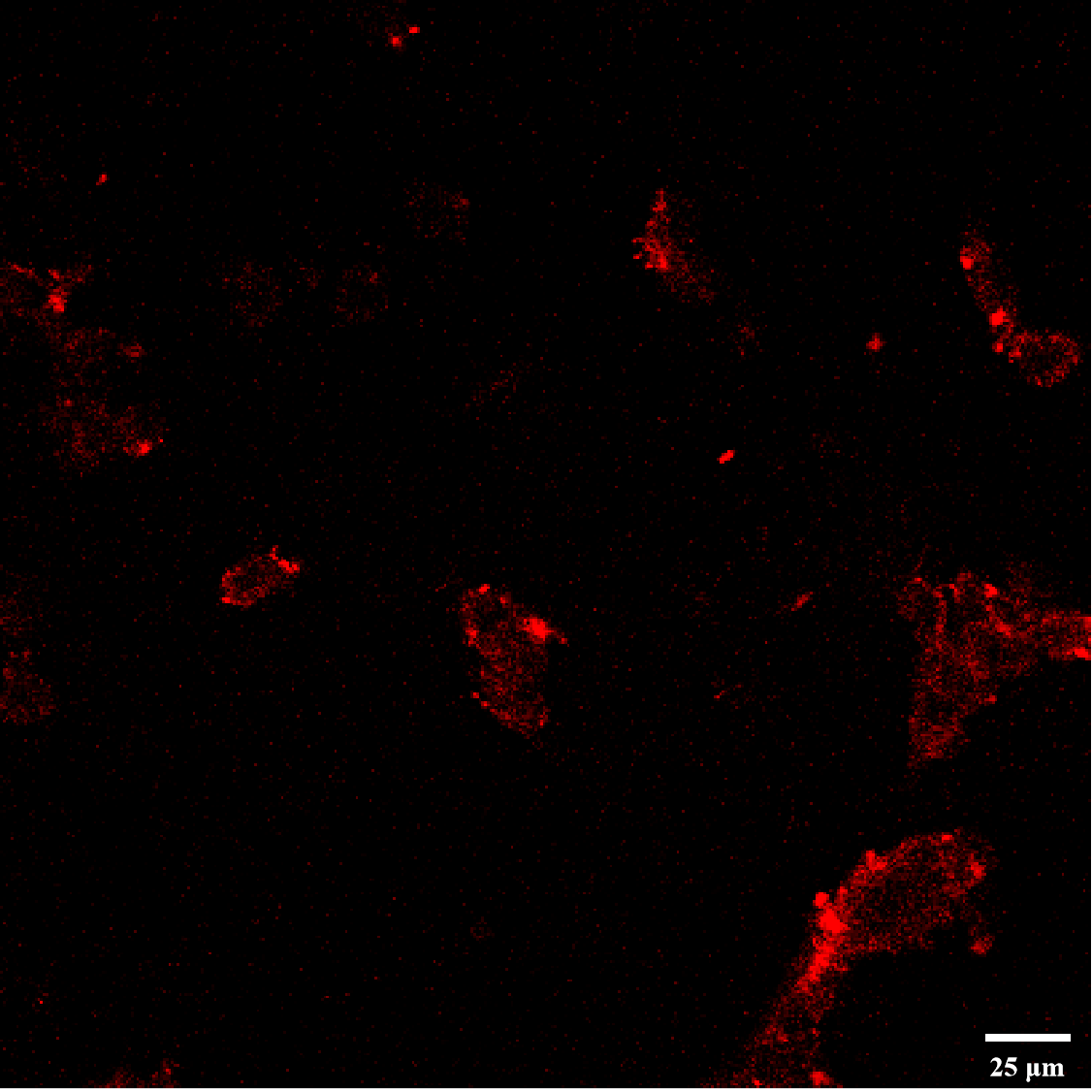

Supplement: Supplemental Information 5 [file peerj-10-12797-s005.zip › Fig-5/H9C2 col1/TGF1-2.tif]

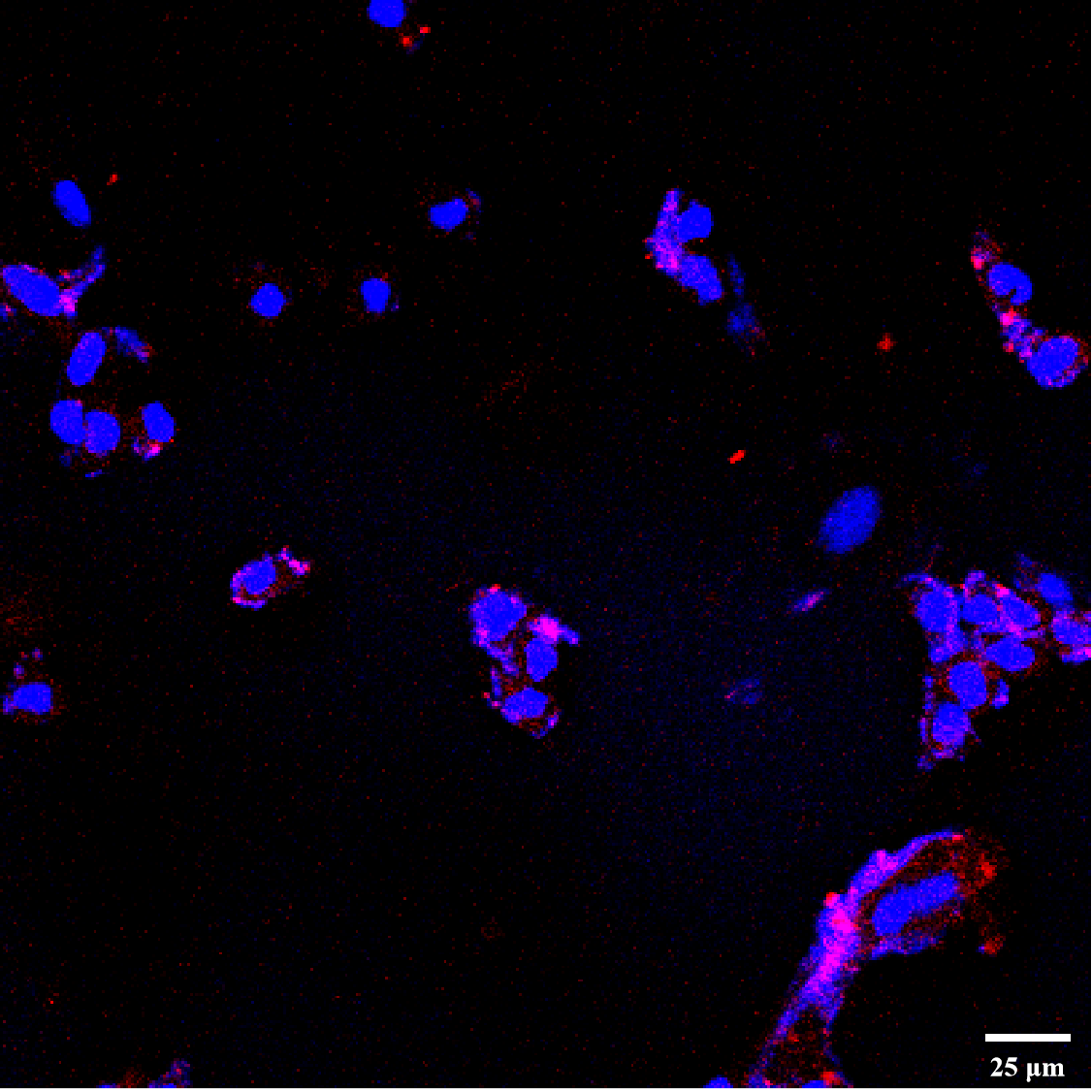

Supplement: Supplemental Information 5 [file peerj-10-12797-s005.zip › Fig-5/H9C2 col1/TGF1.tif]

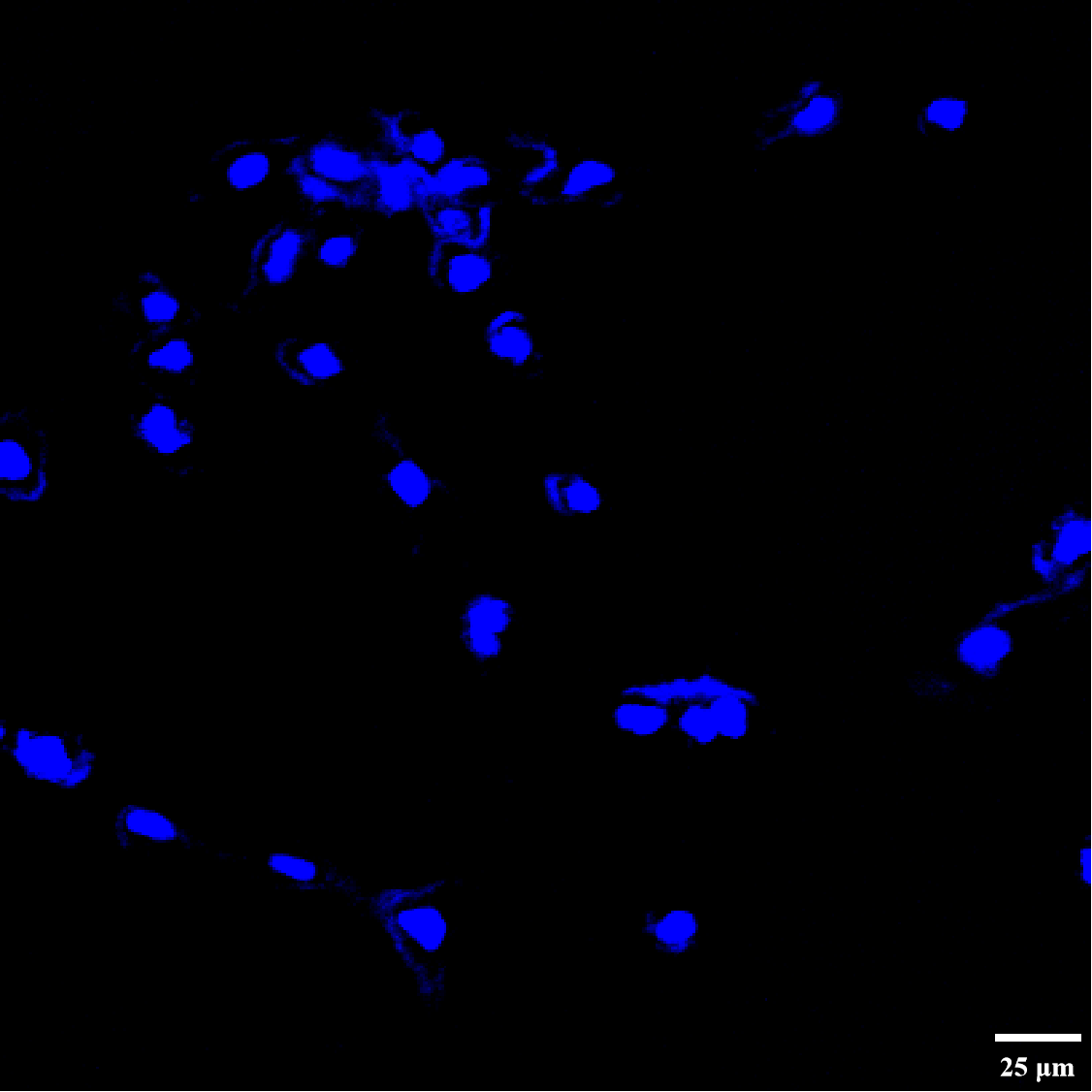

Supplement: Supplemental Information 5 [file peerj-10-12797-s005.zip › Fig-5/H9C2 col1/con1-1.tif]

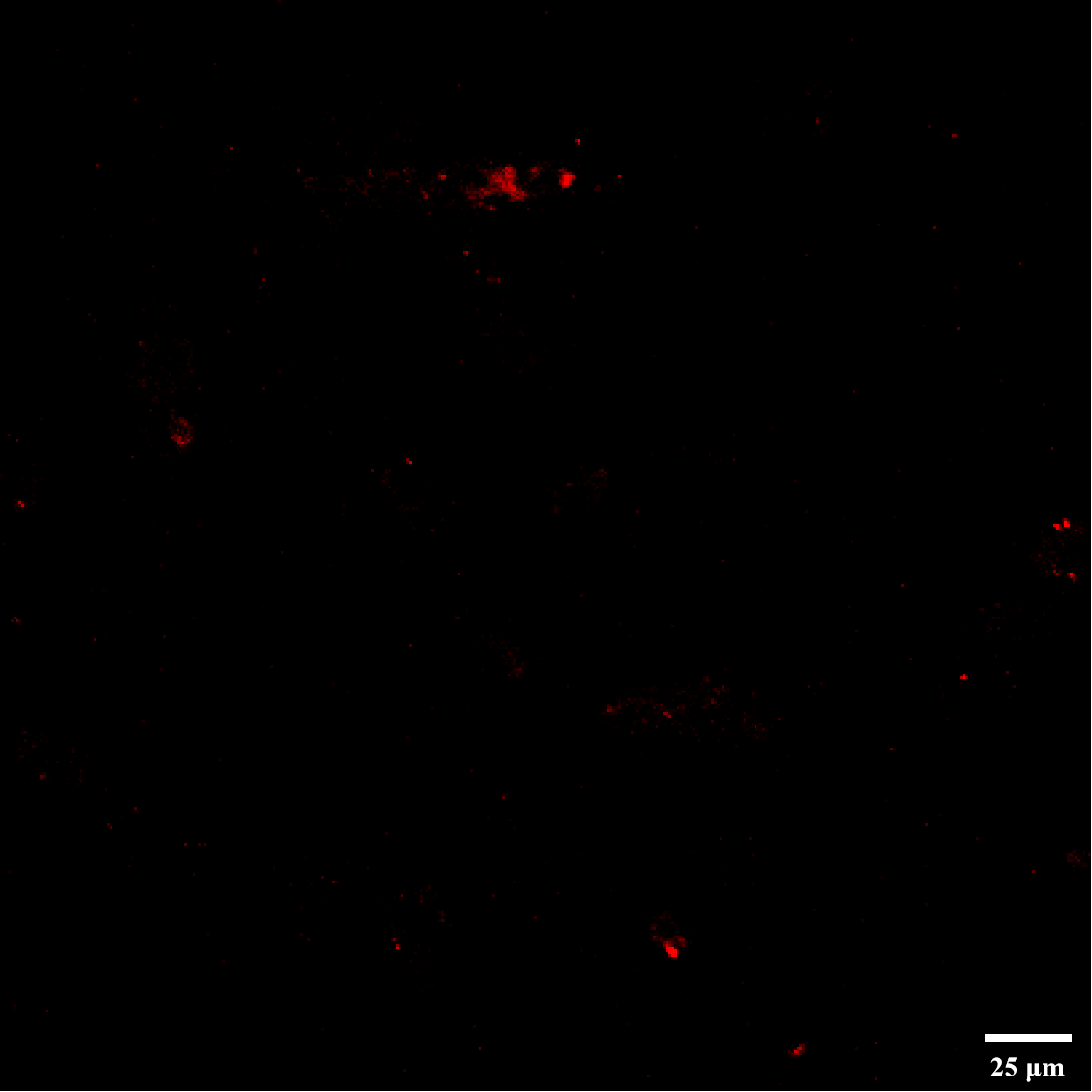

Supplement: Supplemental Information 5 [file peerj-10-12797-s005.zip › Fig-5/H9C2 col1/con1-2.tif]

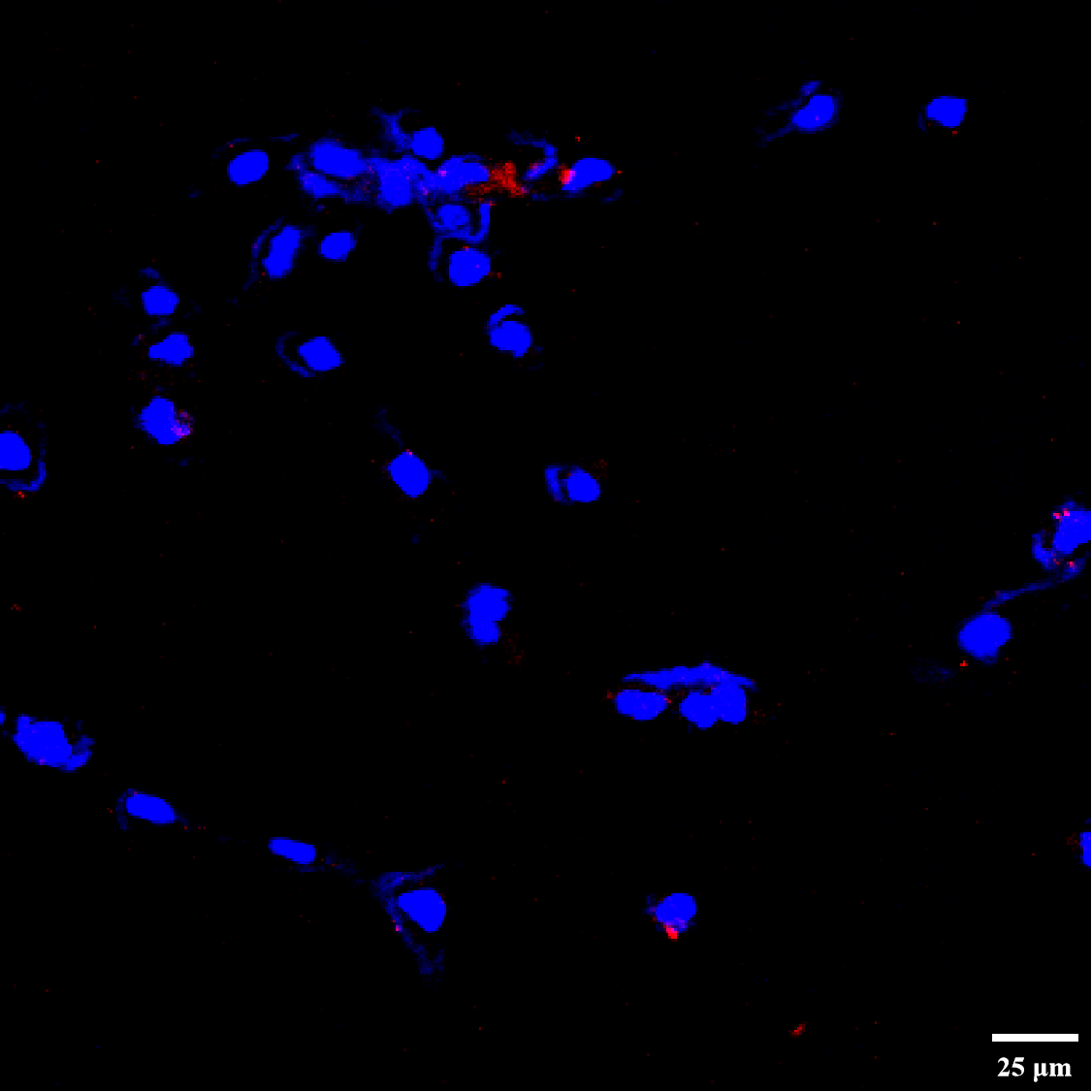

Supplement: Supplemental Information 5 [file peerj-10-12797-s005.zip › Fig-5/H9C2 col1/con1.tif]

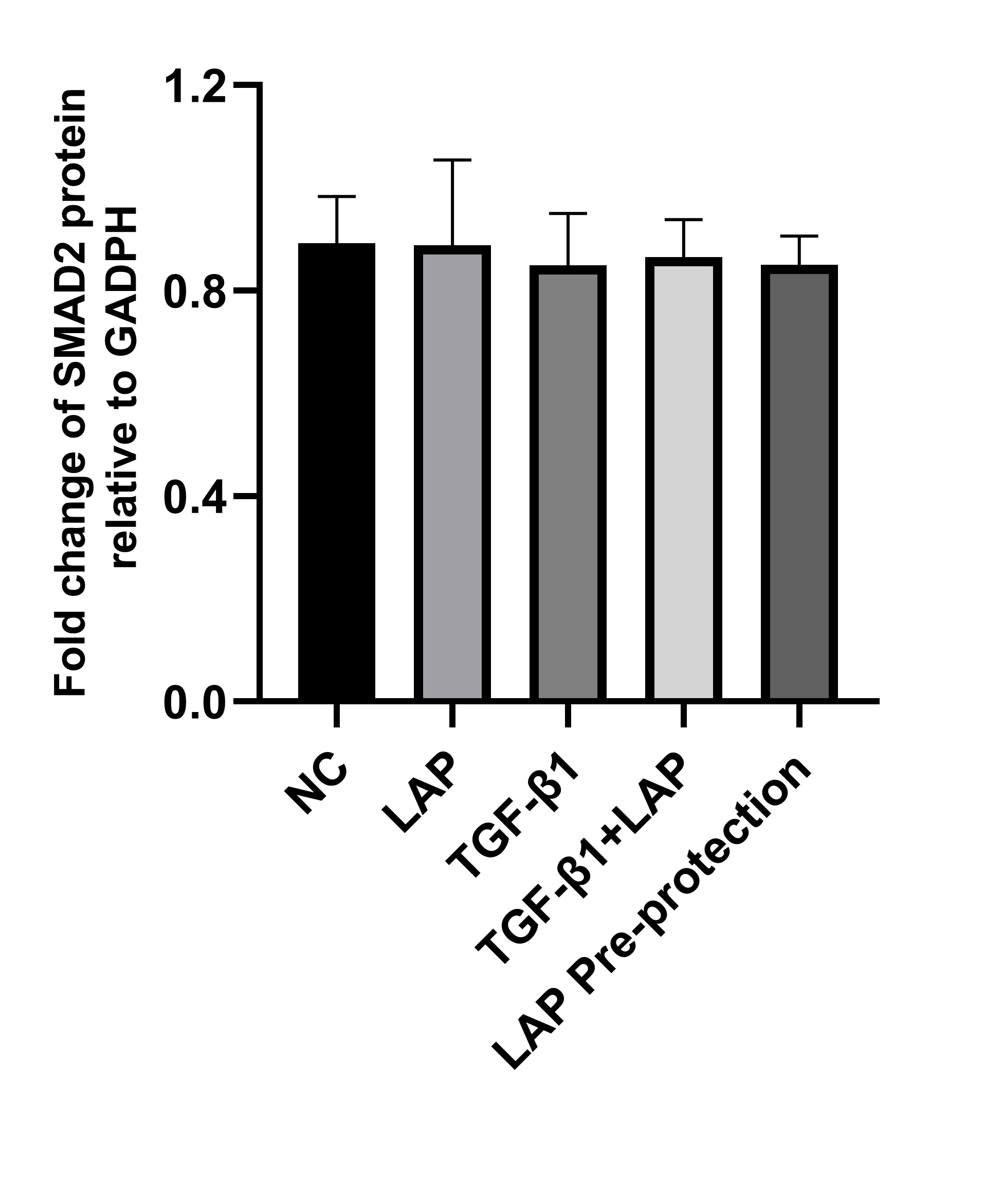

Supplement: Supplemental Information 6 [file peerj-10-12797-s006.zip › Fig 6 Data analysis statistics/Fig 6/Fig 6 smad2.tif]

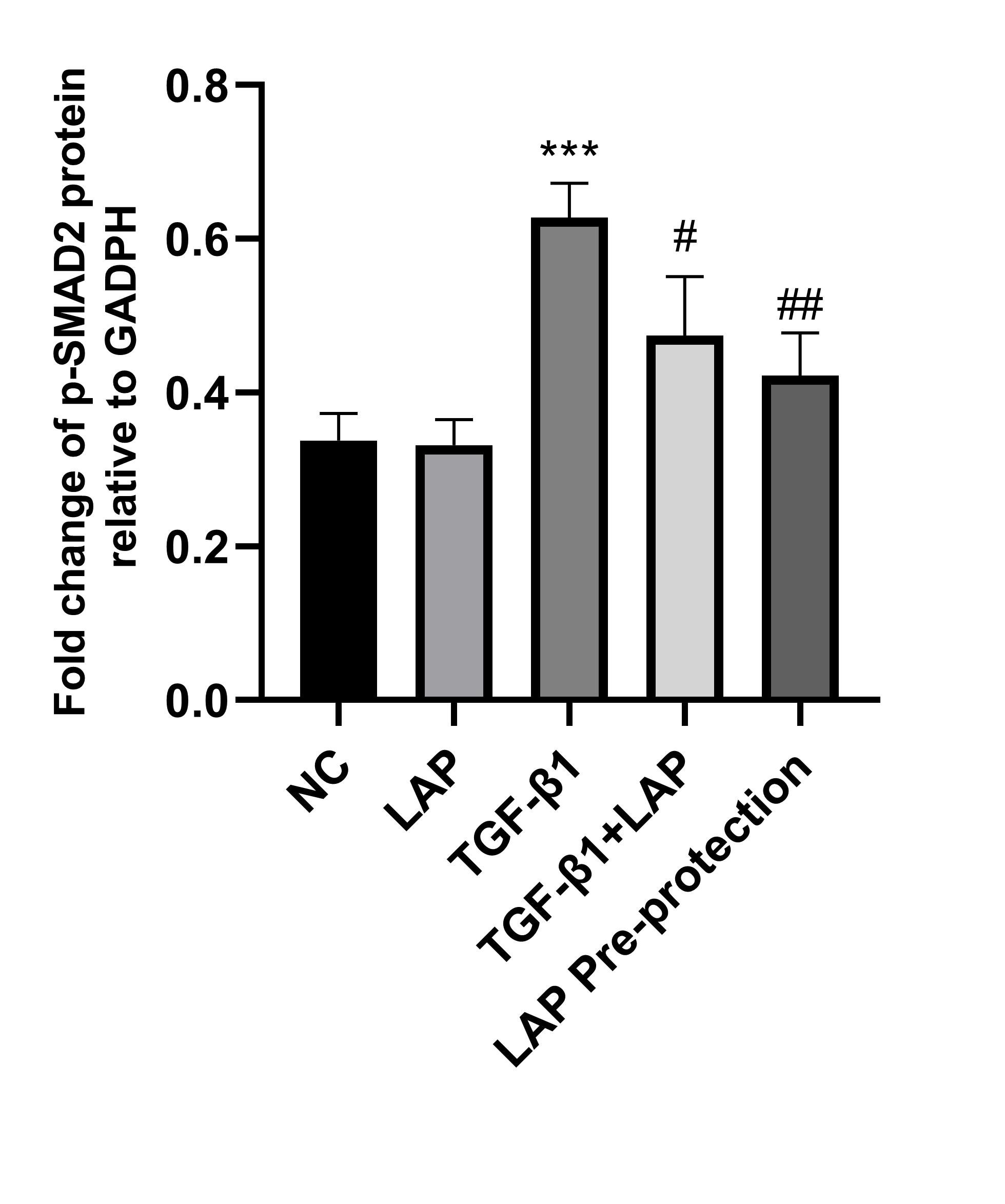

Supplement: Supplemental Information 6 [file peerj-10-12797-s006.zip › Fig 6 Data analysis statistics/Fig 6/Fig6 p-smad2.tif]

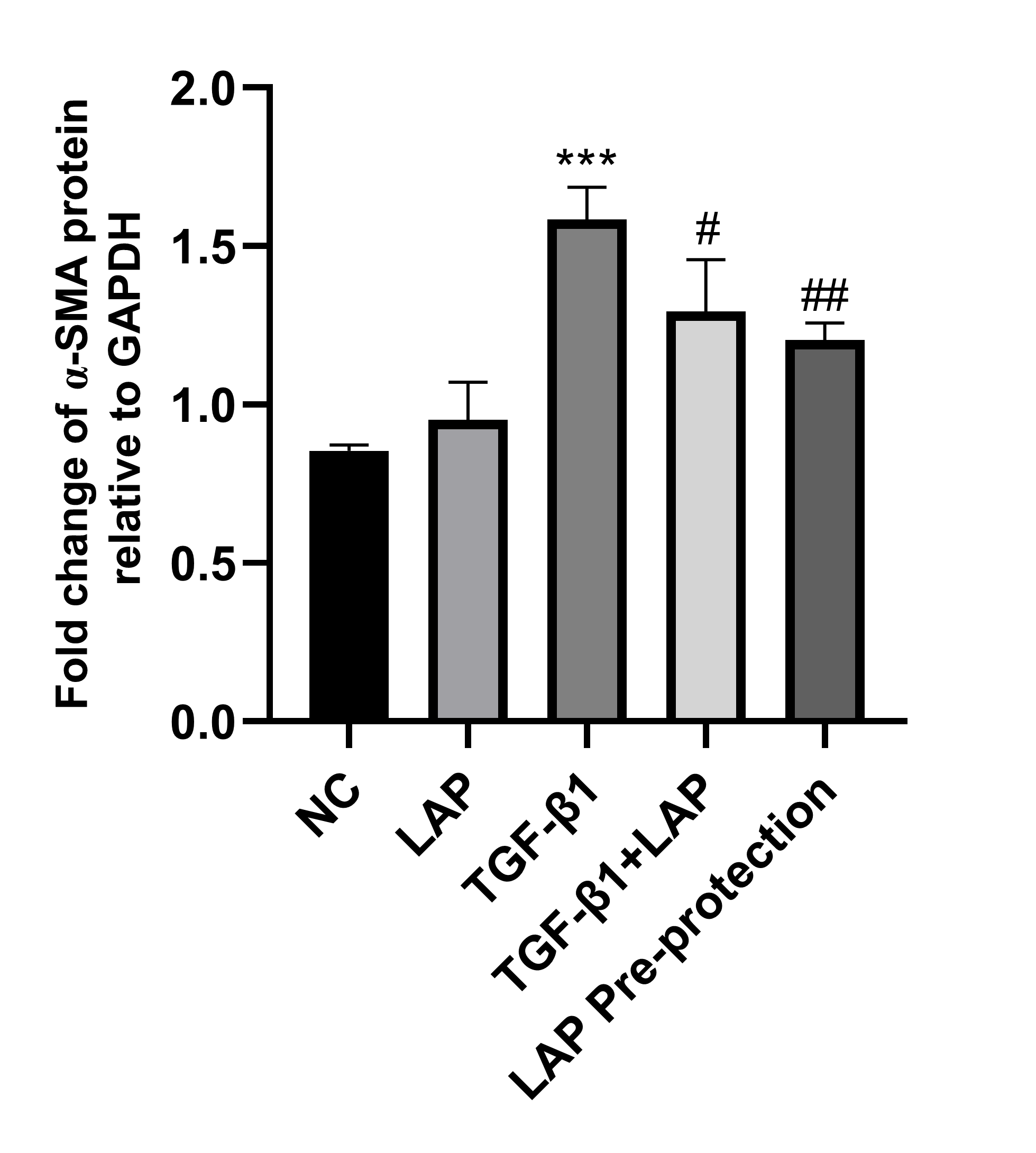

Supplement: Supplemental Information 6 [file peerj-10-12797-s006.zip › Fig 6 Data analysis statistics/Fig 6/Fig6 a┴-SMA.tif]

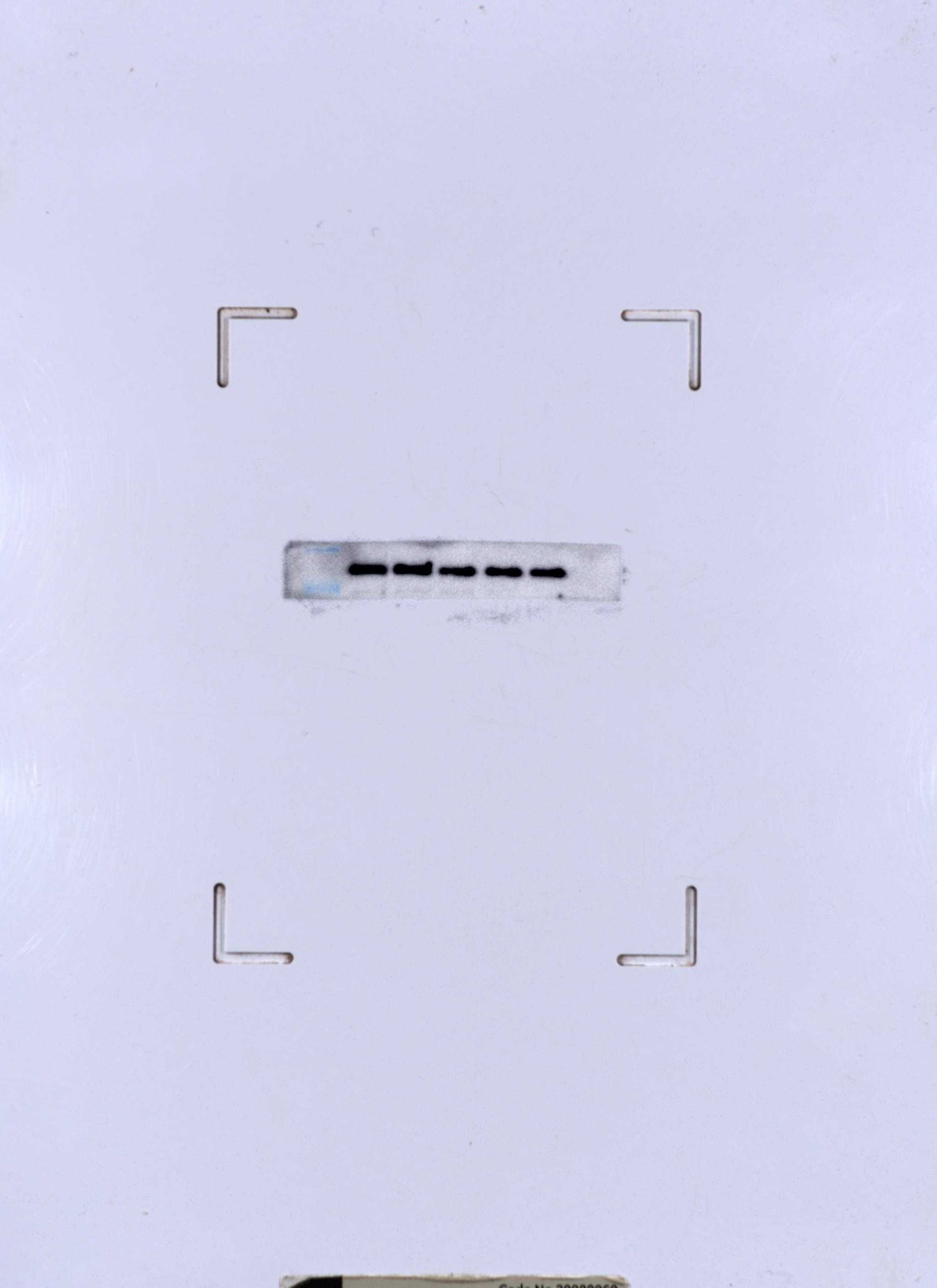

Supplement: Supplemental Information 6 [file peerj-10-12797-s006.zip › Fig 6 Data analysis statistics/Fig 6/GAPDH/GAPDH1 Ch+Marker.jpg]

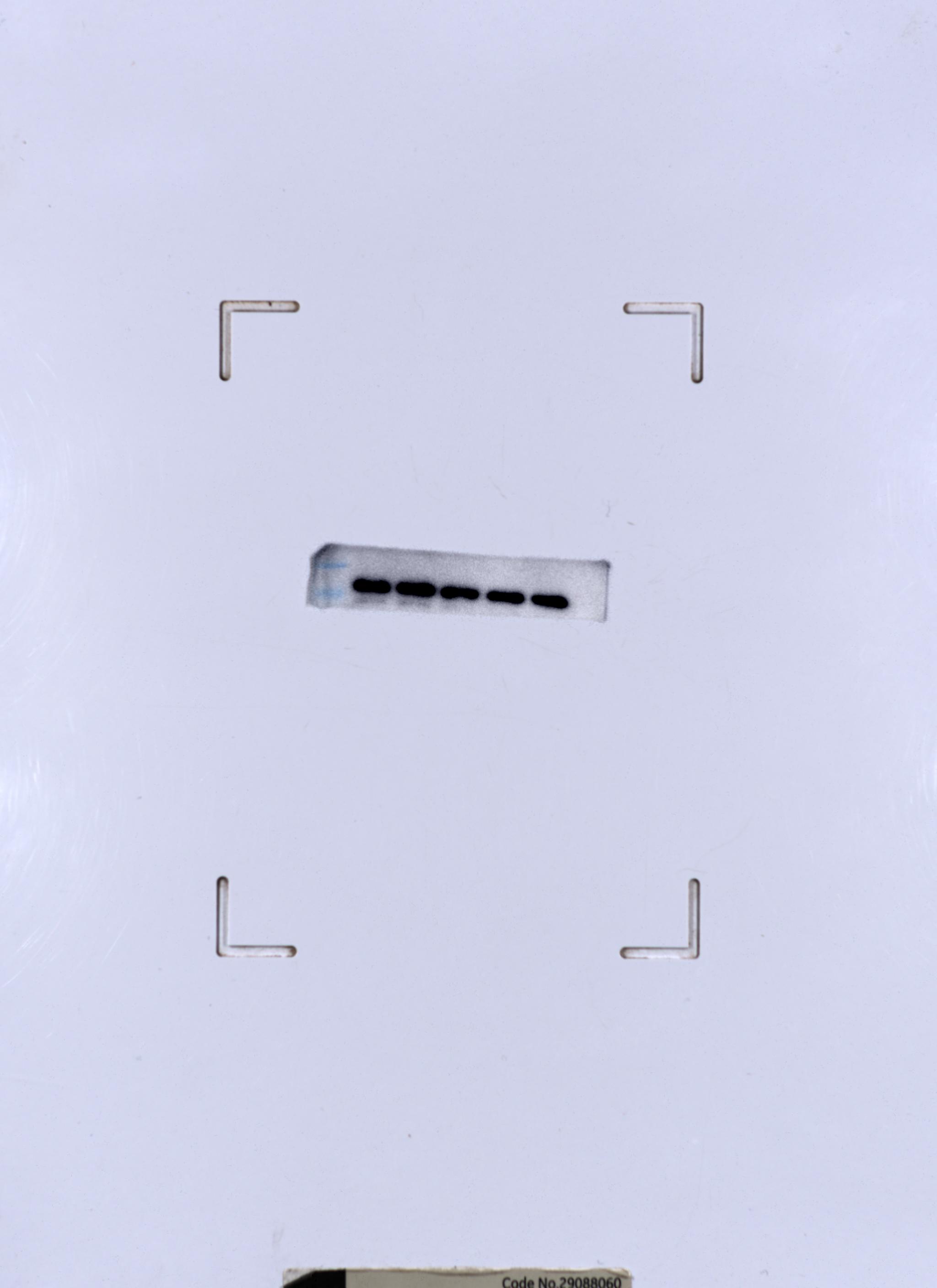

Supplement: Supplemental Information 6 [file peerj-10-12797-s006.zip › Fig 6 Data analysis statistics/Fig 6/GAPDH/GAPDH2 Ch+Marker.jpg]

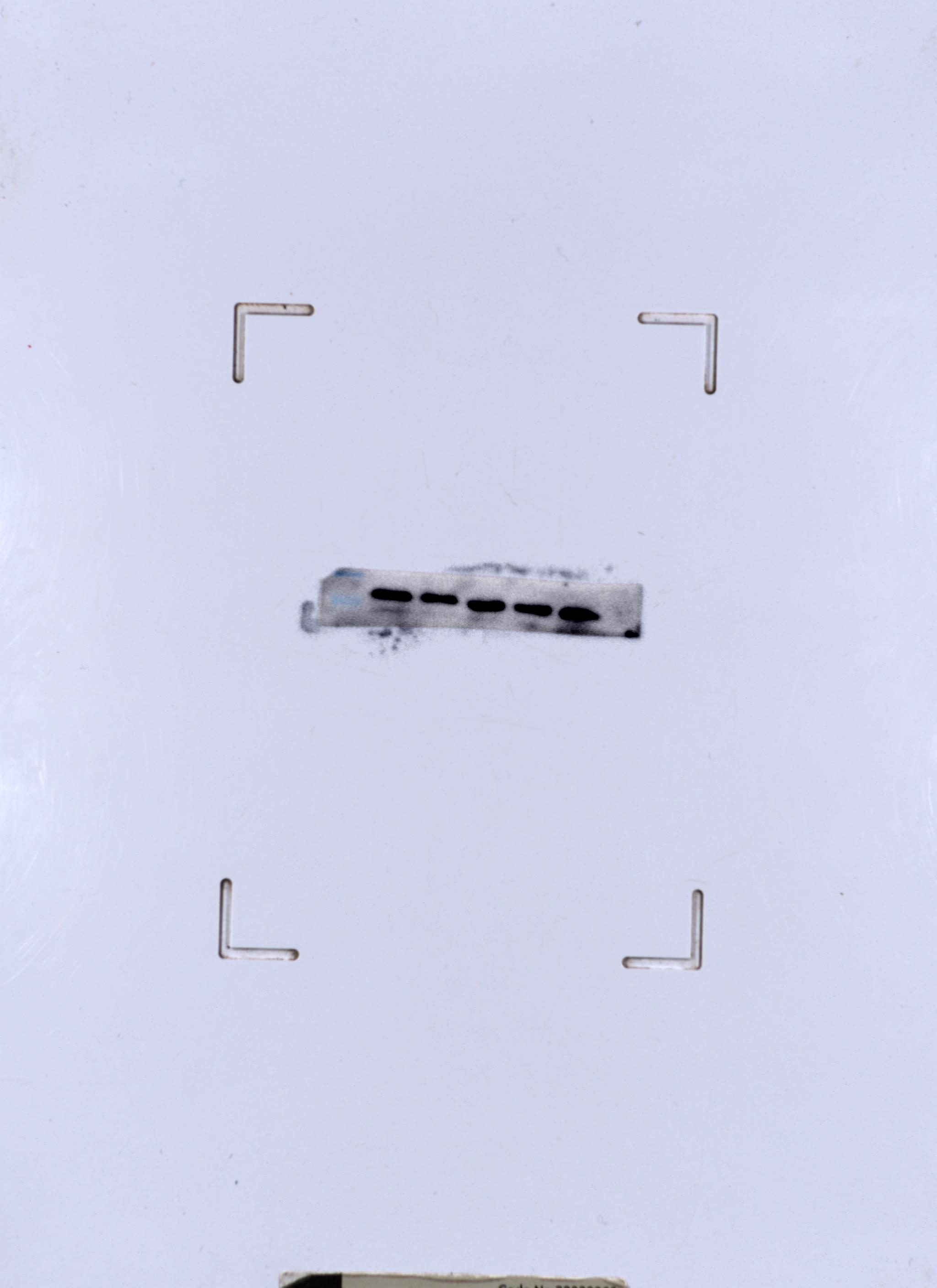

Supplement: Supplemental Information 6 [file peerj-10-12797-s006.zip › Fig 6 Data analysis statistics/Fig 6/GAPDH/GAPDH3 Ch+Marker.jpg]

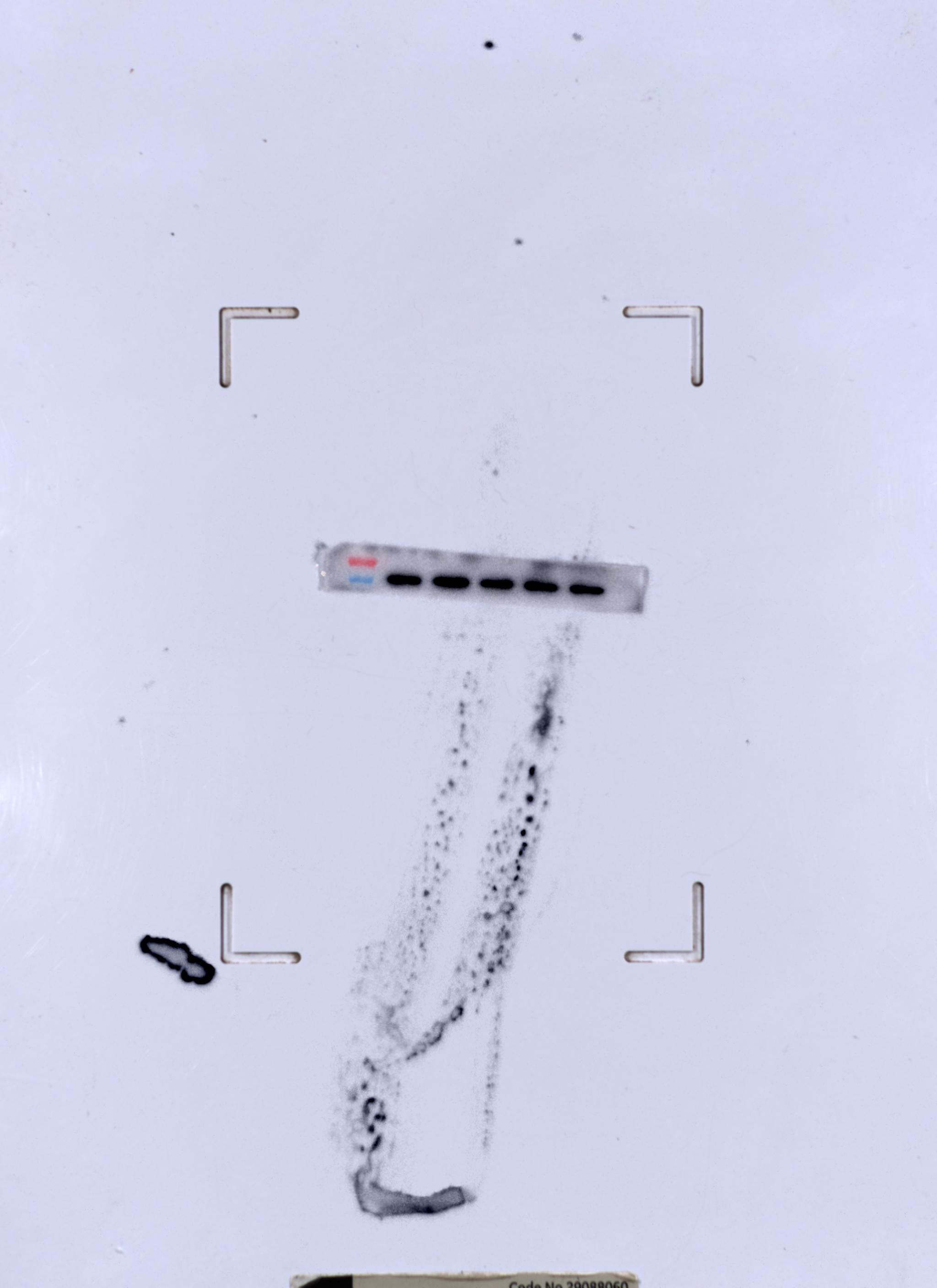

Supplement: Supplemental Information 6 [file peerj-10-12797-s006.zip › Fig 6 Data analysis statistics/Fig 6/SMAD2/SAMD2 1 Ch+Marker.jpg]

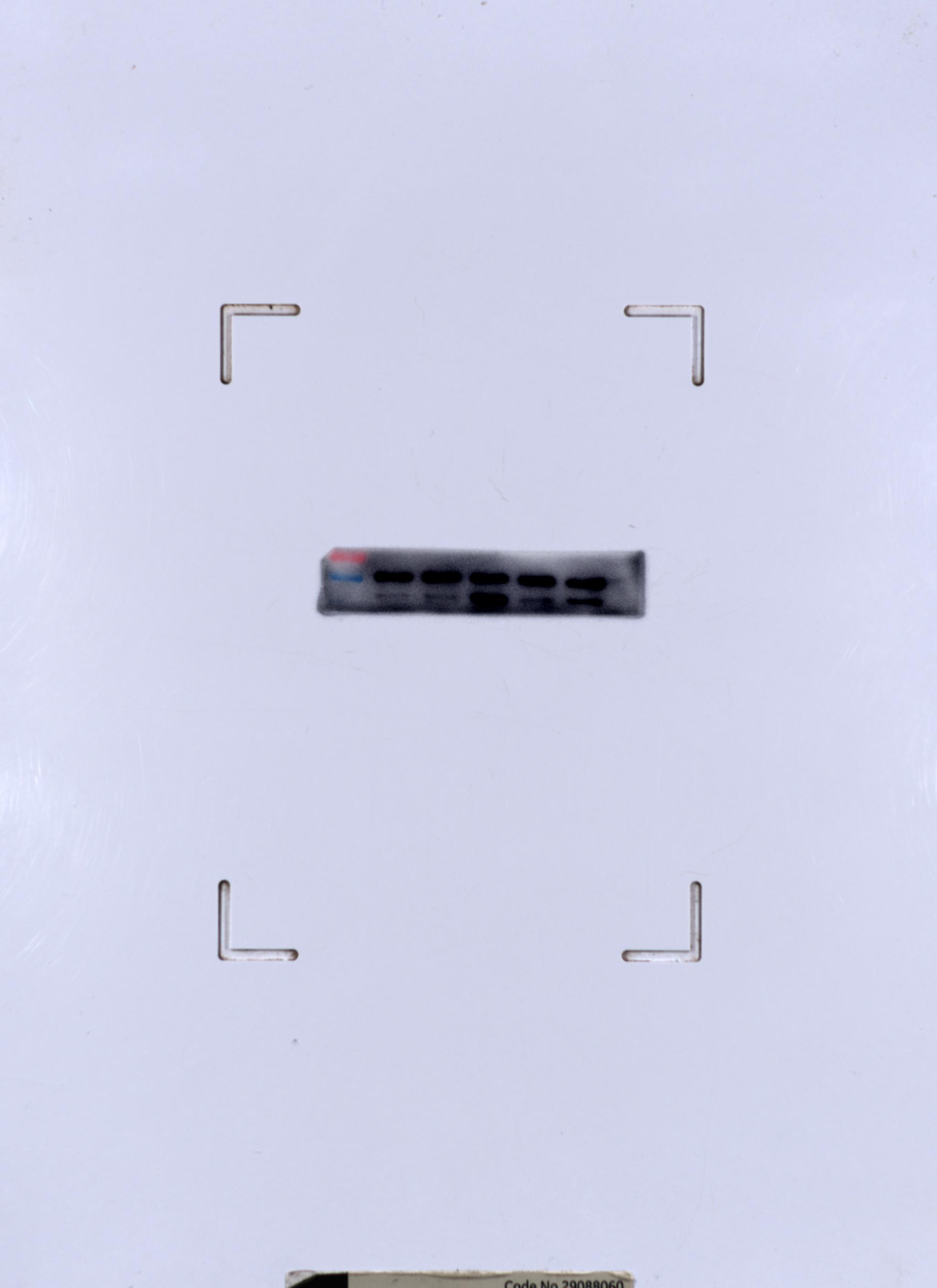

Supplement: Supplemental Information 6 [file peerj-10-12797-s006.zip › Fig 6 Data analysis statistics/Fig 6/SMAD2/SMAD2 2 Ch+Marker.jpg]

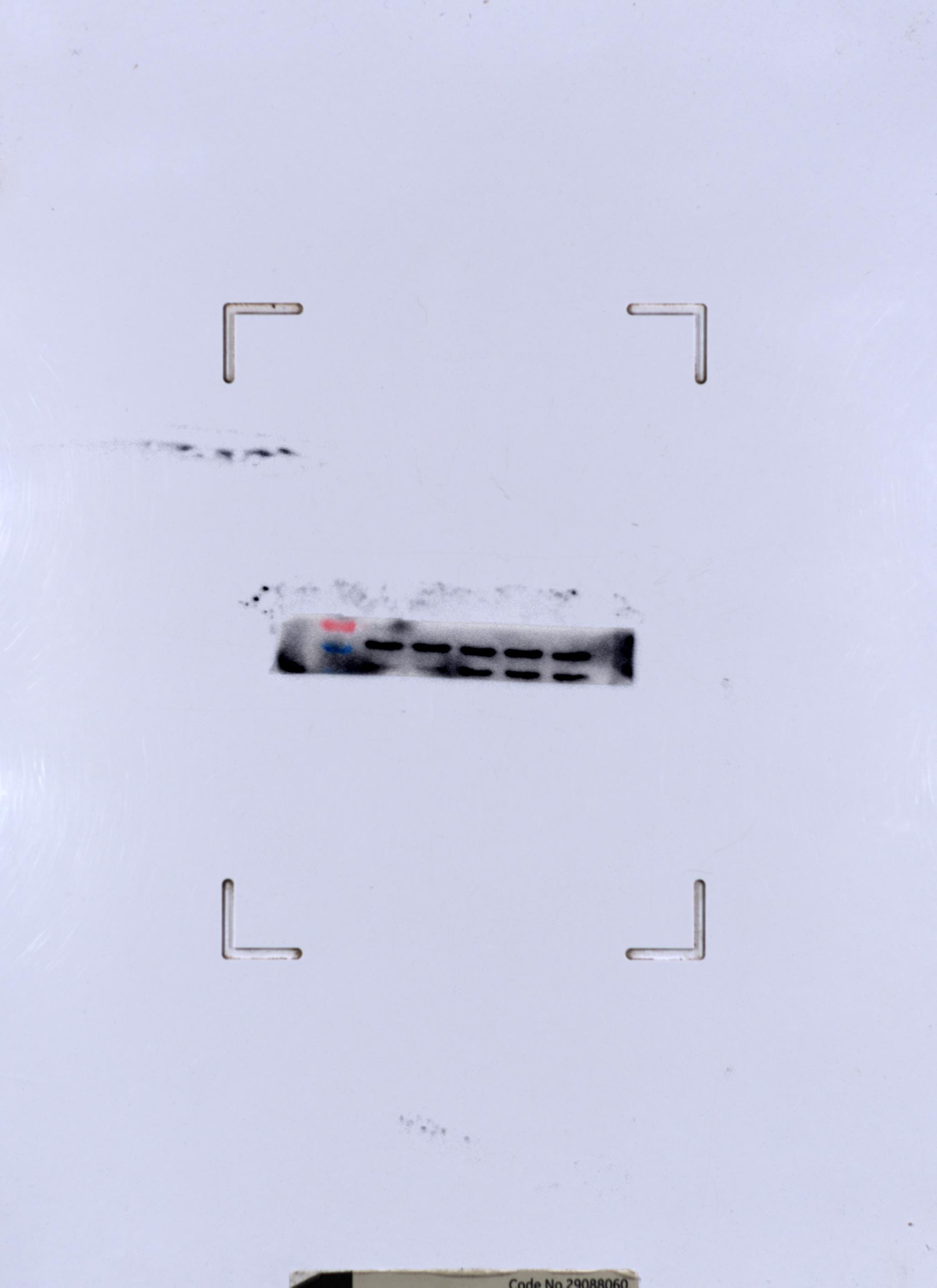

Supplement: Supplemental Information 6 [file peerj-10-12797-s006.zip › Fig 6 Data analysis statistics/Fig 6/SMAD2/SMAD2 3 Ch+Marker.jpg]

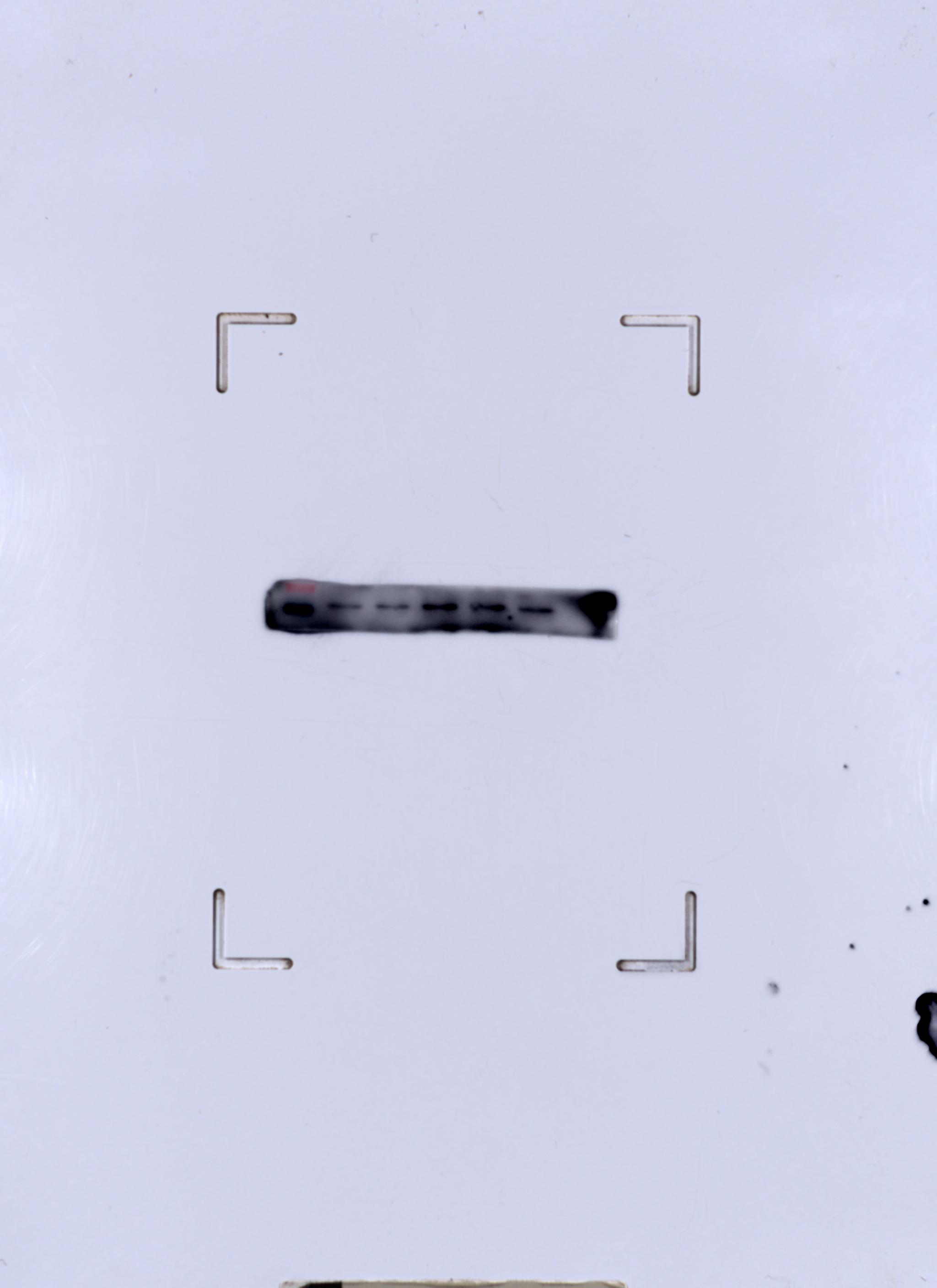

Supplement: Supplemental Information 6 [file peerj-10-12797-s006.zip › Fig 6 Data analysis statistics/Fig 6/p-smad2/P-SMAD2 1 Ch+Marker.jpg]

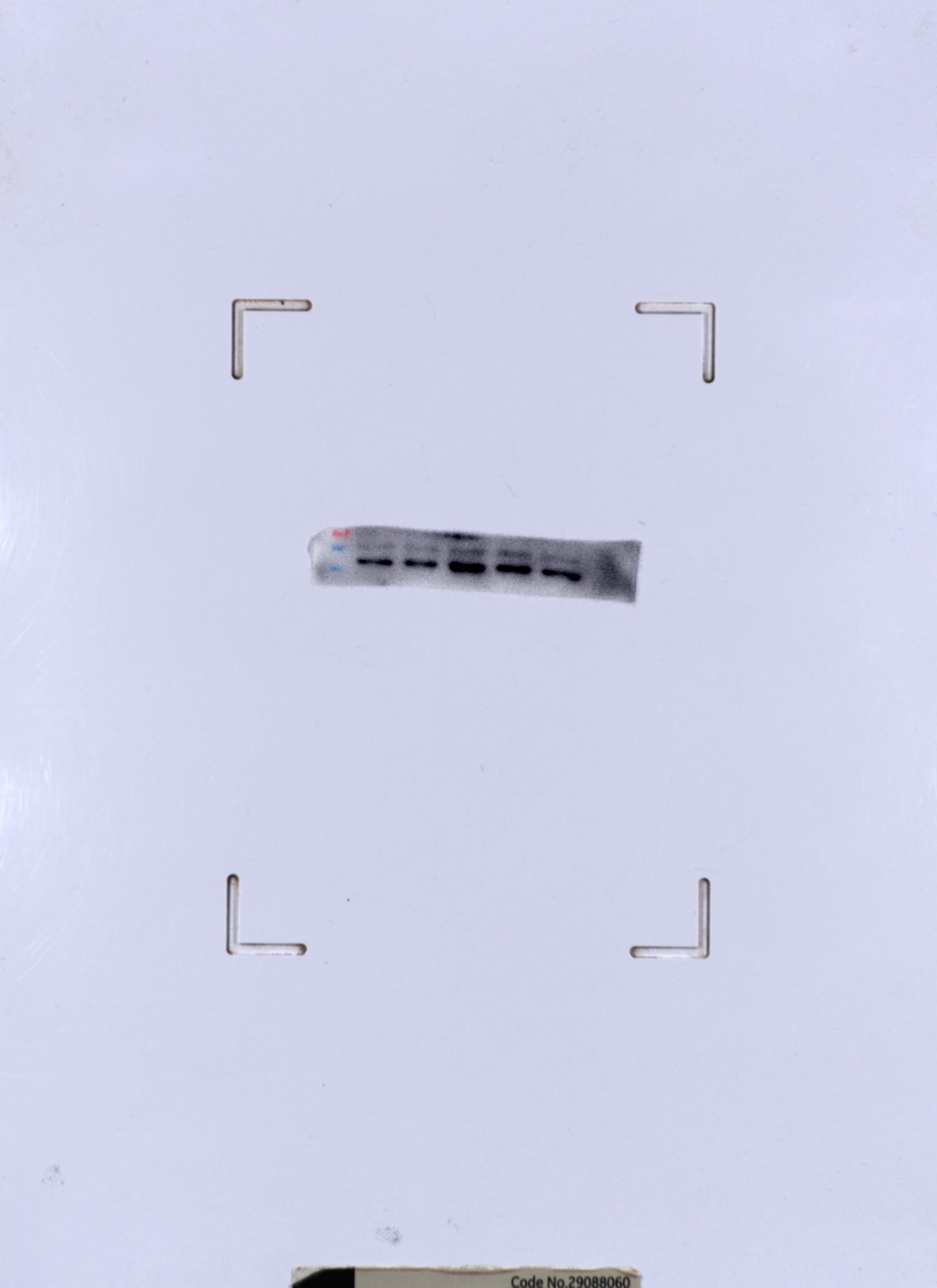

Supplement: Supplemental Information 6 [file peerj-10-12797-s006.zip › Fig 6 Data analysis statistics/Fig 6/p-smad2/P-SMAD2 2 Ch+Marker.jpg]

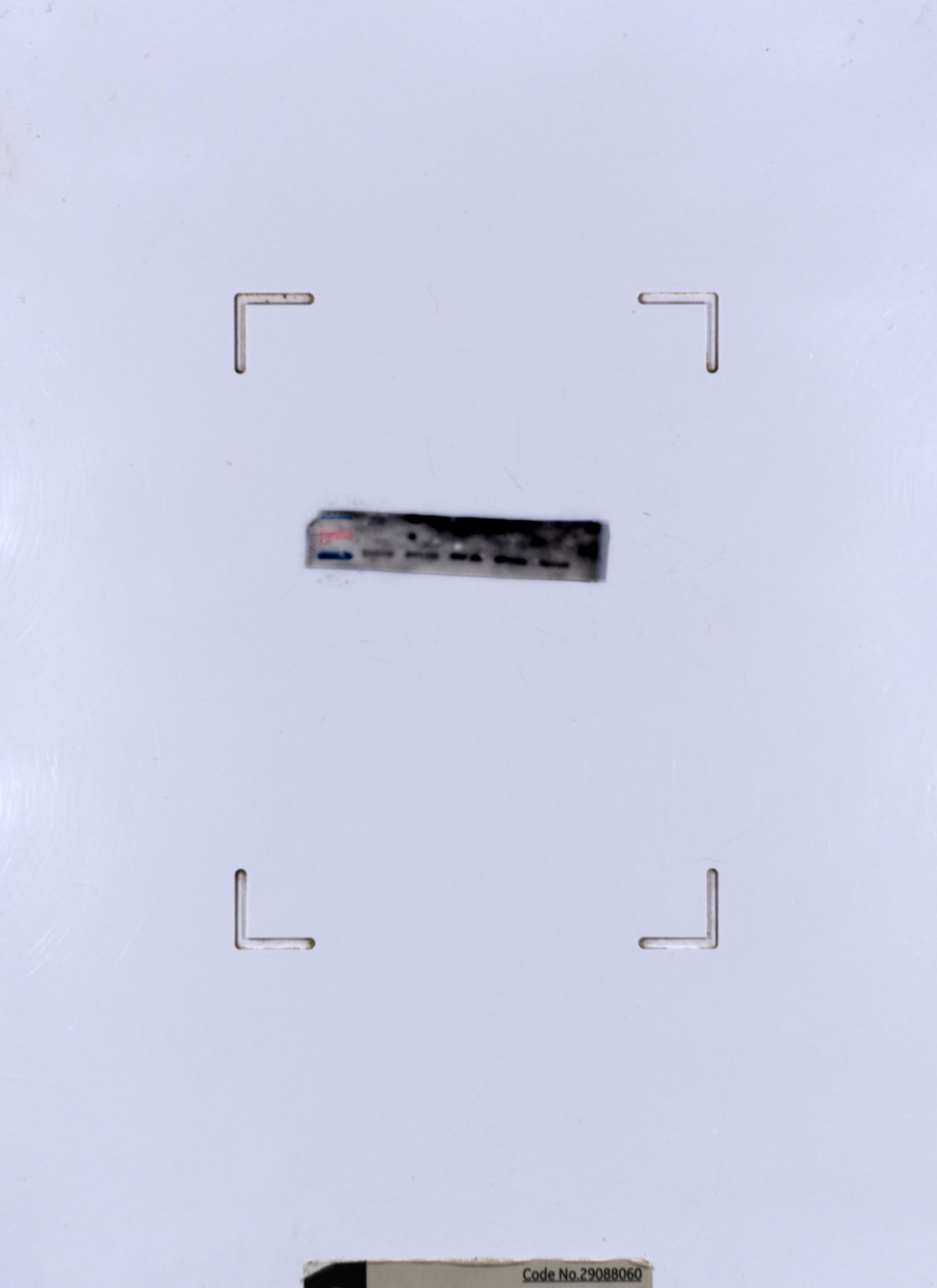

Supplement: Supplemental Information 6 [file peerj-10-12797-s006.zip › Fig 6 Data analysis statistics/Fig 6/p-smad2/P-SMAD2 3 Ch+Marker.jpg]

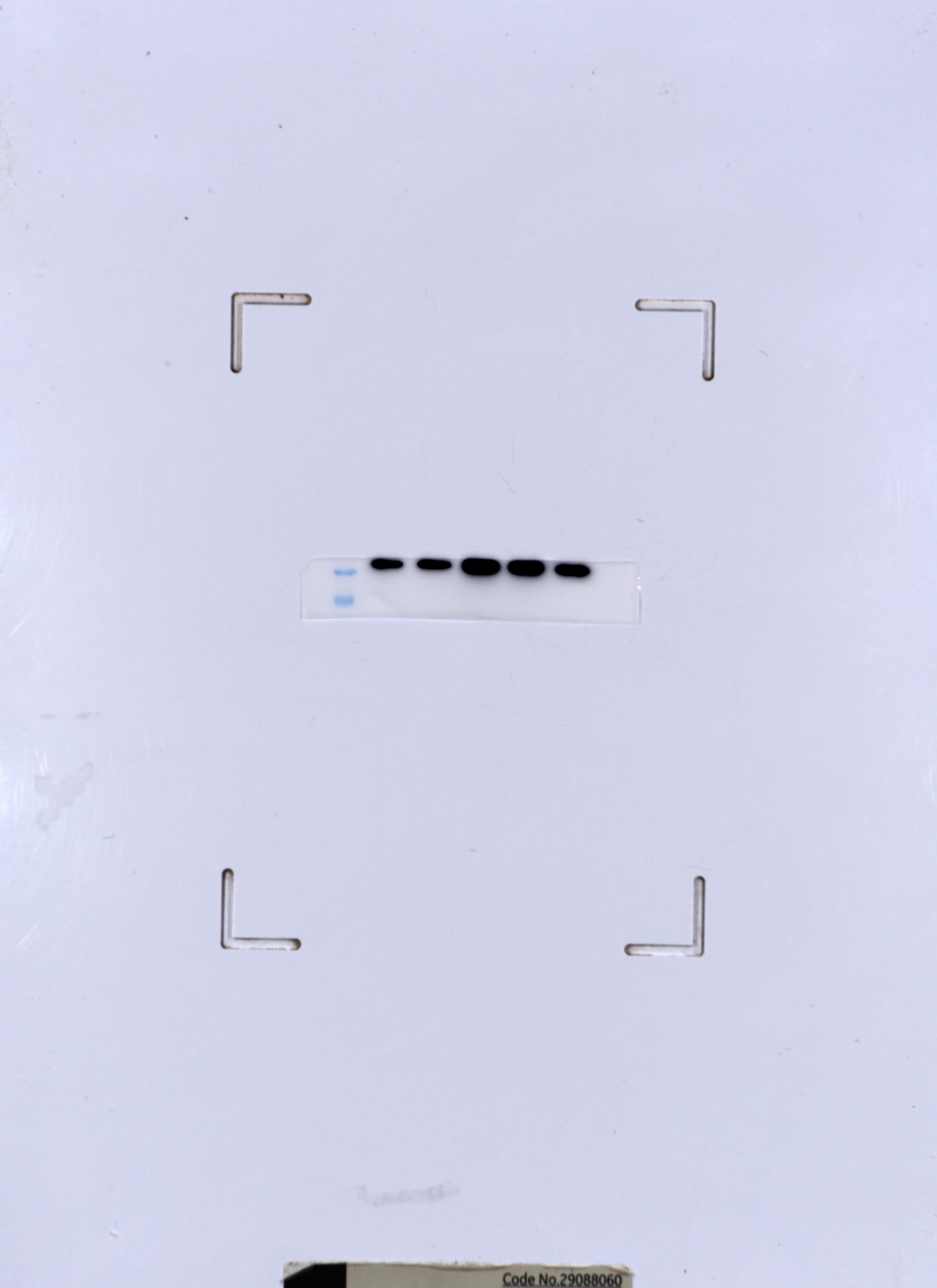

Supplement: Supplemental Information 6 [file peerj-10-12797-s006.zip › Fig 6 Data analysis statistics/Fig 6/a┴-SMA/SMA 1 Ch+Marker.jpg]

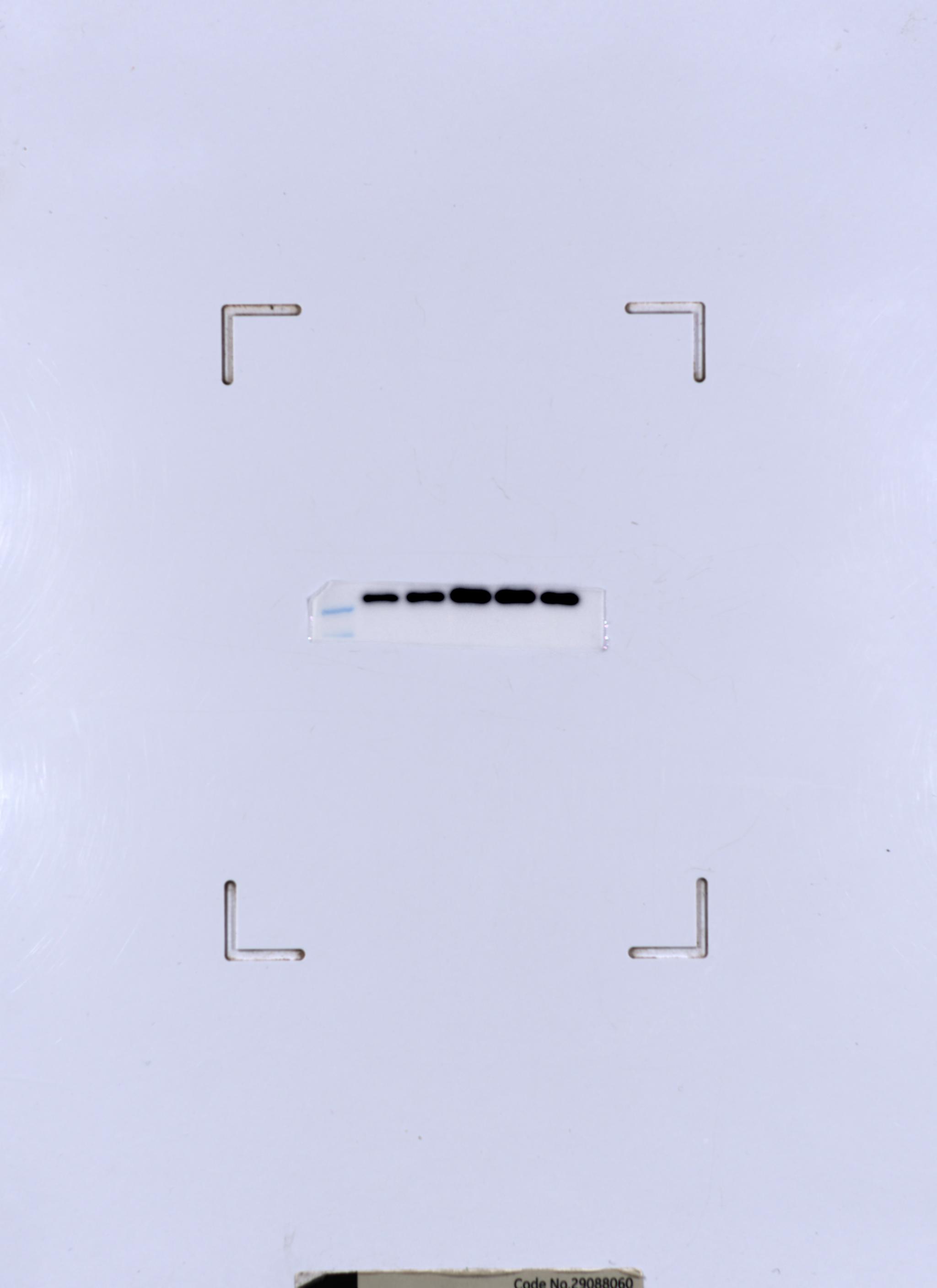

Supplement: Supplemental Information 6 [file peerj-10-12797-s006.zip › Fig 6 Data analysis statistics/Fig 6/a┴-SMA/SMA 2 Ch+Marker.jpg]

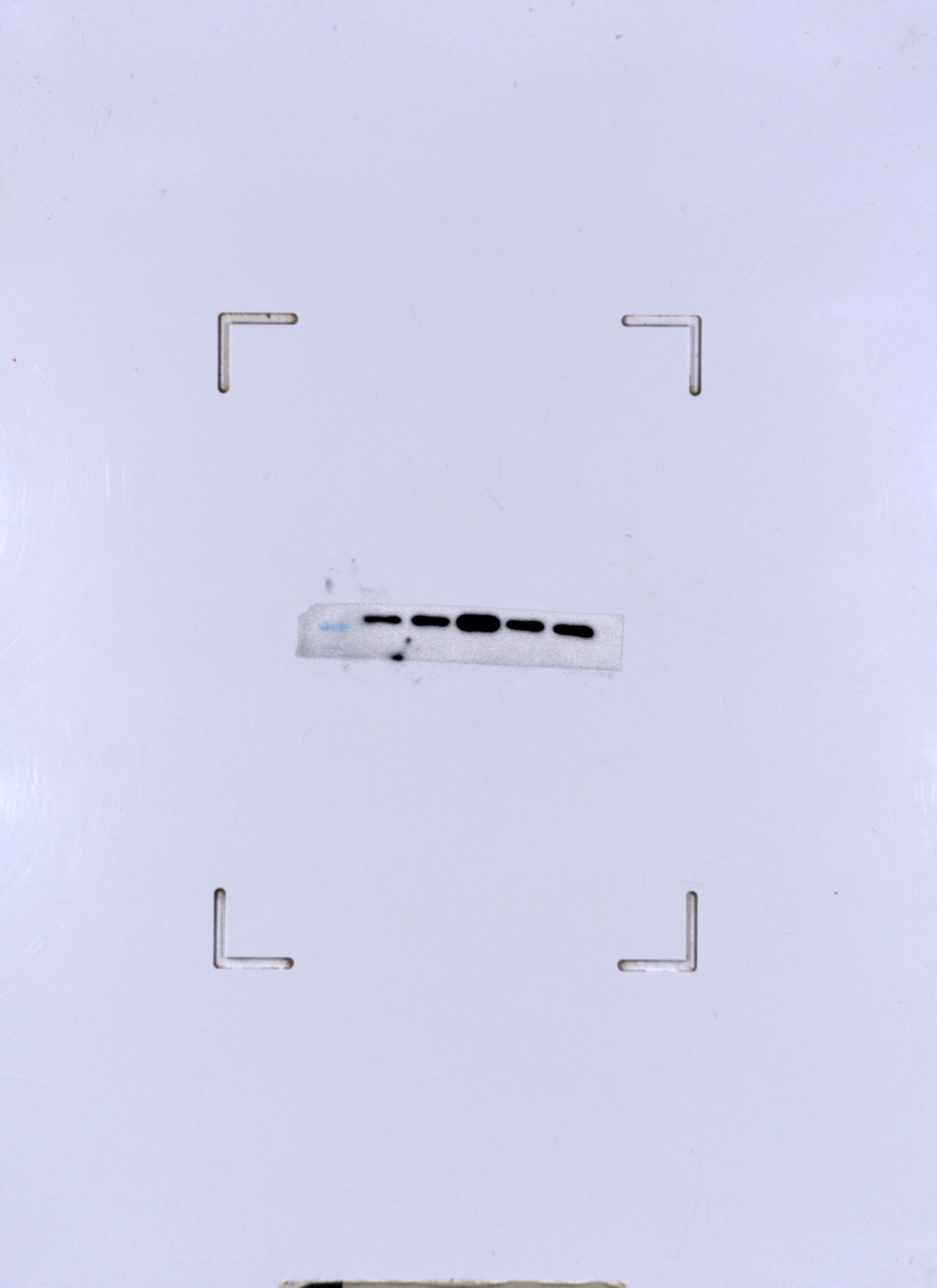

Supplement: Supplemental Information 6 [file peerj-10-12797-s006.zip › Fig 6 Data analysis statistics/Fig 6/a┴-SMA/SMA 3 Ch+Marker.jpg]
